# Geographic sources of ozone air pollution and mortality burden in Europe

**DOI:** 10.1038/s41591-024-02976-x

**Published:** 2024-06-03

**Authors:** Hicham Achebak, Roger Garatachea, María Teresa Pay, Oriol Jorba, Marc Guevara, Carlos Pérez García-Pando, Joan Ballester

**Affiliations:** 1 Inserm, France Cohortes, Paris, France; 2 ISGlobal, Barcelona, Spain; 3 Barcelona Supercomputing Center (BSC), Barcelona, Spain; 4 Department of Genetics, Microbiology and Statistics, University of Barcelona (UB), Barcelona, Spain; 5 Catalan Institution for Research and Advanced Studies (ICREA), Barcelona, Spain

**Keywords:** Risk factors, Policy

## Abstract

Ground-level ozone (O_3_) is a harmful air pollutant formed in the atmosphere by the interaction between sunlight and precursor gases. Exposure to current O_3_ levels in Europe is a major source of premature mortality from air pollution. However, mitigation actions have been mainly designed and implemented at the national and regional scales, lacking a comprehensive assessment of the geographic sources of O_3_ pollution and its associated health impacts. Here we quantify both national and imported contributions to O_3_ and their related mortality burden across 813 contiguous regions in 35 European countries, representing about 530 million people. Imported O_3_ contributed to 88.3% of all O_3_-attributable deaths (intercountry range 83–100%). The greatest share of imported O_3_ had its origins outside the study domain (that is, hemispheric sources), which was responsible for 56.7% of total O_3_-attributable mortality (range 42.5–87.2%). It was concluded that achieving the air-quality guidelines set out by the World Health Organization and avoiding the health impacts of O_3_ require not only the implementation of national or coordinated pan-European actions but also global strategies.

## Abstract (web summary)

A health impact assessment found that 88% of ozone-related mortality in 35 European countries is attributable to transboundary transported ozone.

## Main

Ground-level ozone (O_3_) is a harmful air pollutant formed in the troposphere by the interaction between sunlight and precursor gases, mainly nitrogen oxides (NO_x_) and volatile organic compounds (VOCs), from natural and anthropogenic sources. High ambient O_3_ levels are observed especially during the warm season, and these are associated with a range of adverse respiratory health outcomes such as aggravation of asthma, chronic obstructive pulmonary disease, lower lung function and infections, leading in the most severe cases to hospitalization and death^1–3^.

Exposure to current O_3_ levels in Europe is a major source of premature mortality from air pollution, especially in summer, and its impact has increased over time due to the effect of rising temperatures on O_3_ concentrations^4,5^. However, according to the European Environmental Agency (EEA)^4^, >95% of the population in Europe remains exposed to O_3_ levels that exceed the air-quality guidelines (daily maximum 8-h average of 100 µg m^−3^) set out by the World Health Organization (WHO)^6^, within the context of accelerated urbanization and demographic aging that increases the background health risks of exposed populations.

The health impacts of O_3_, and generally of any air pollutant, are, however, far from being a local issue^7,8^. O_3_ concentrations in a given location greatly depend on the tropospheric transport of the pollutant itself, or that of its precursors, from far-distant sources^9,10^. For instance, a study^11^ in south-western Europe showed that imported O_3_ is the largest contributor to ground-level O_3_ concentrations, accounting for 46–68% of daily surface levels. This emphasizes the need for coordinated actions among countries to reduce O_3_ concentrations and health impacts, given that no effective strategy may be developed without international agreement on air pollution reduction.

Several studies have estimated the mortality burden attributable to tropospheric O_3_ in different settings^2,12–14^, but a continent-wide assessment of the contribution of O_3_ pollution by geographical source is still lacking. In the present study we quantify the health impacts of transboundary-transported O_3_ (daily maximum 8-h average) in Europe. Our focus is on the transboundary health effects of O_3_ due to its ability to persist over long distances in the free troposphere during transport, in contrast to its precursors such as NO_2_ that have shorter lifetimes. This health impact assessment integrates air-quality modeling as a source-apportionment method and exposure–response associations characterizing the effects of O_3_ on human mortality as a starting point for the development of a coordinated agenda to effectively minimize the health effects of air pollution over the continent.

## Results

### Descriptive statistics

**Fig. 1:**
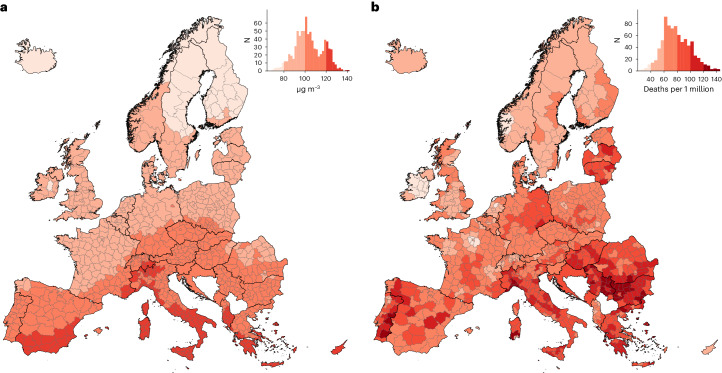
O_3_ levels and associated mortality during the warm season (May–September), 2015–2017. **a**, Average daily mean 8-h maximum O_3_ (µg m^−^^3^). **b**, Mortality (annual deaths per 1 million population) attributable to O_3_. **a**,**b**, Histograms depict both the color legend and the number of regions for each value.

The results of this Europe-wide study are presented at both the national (35 countries) and subnational (813 regions) level. The average concentration of O_3_ across countries and the study period was 101.9 μg m^−^^3^, ranging from 76.7 μg m^−^^3^ in Finland to 130.1 μg m^−^^3^ in Malta (Supplementary Table 1). As expected, the spatial distribution of O_3_ is found to be latitudinally oriented, with concentrations decreasing northwards, given that the warmer temperatures in the south favor the formation of O_3_, especially in summer (Fig. 1a). The estimated number of deaths attributable to O_3_ over the entire European domain during the warm seasons of 2015–2017 was 114,447 (95% empirical confidence interval (eCI) 76,539–152,108), resulting in an attributable mortality rate of 72.0 (95% eCI 48.1–95.6) annual deaths per 1 million inhabitants. The highest mortality burdens are estimated for those countries with the largest populations (Germany, Italy, France, the UK, Spain and Poland; Supplementary Table 2) whereas the highest mortality rates were in the south-eastern countries (Bulgaria, Serbia, Croatia, Hungary, Greece and Romania; Fig. 1b and Supplementary Table 3).

### O_3_ levels and associated premature mortality

**Fig. 2:**
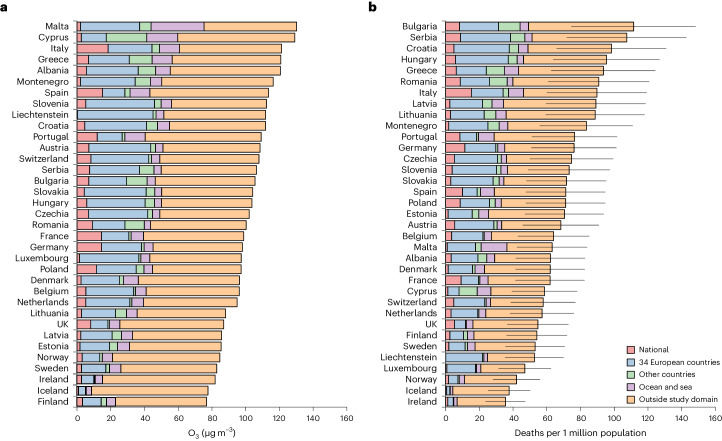
O_3_ levels and associated mortality according to O_3_ emission sources in 35 European countries, 2015–2017. **a**, Average daily mean 8-h maximum O_3_ (µg m^−^^3^). **b**, Mortality attributable to O_3_ (annual deaths per 1 million population). Horizontal bars represent 95% eCI of overall O_3_-attributable mortality (that is, the sum of the five contribution sources).

Figure 2 and Supplementary Tables 1 and 2 show the contribution to O_3_ concentrations and attributable deaths for each country analyzed according to the source of origin of O_3_ and its precursor emissions: (1) national, (2) the 34 other European countries, (3) other countries inside the study domain, (4) ocean and sea inside the study domain and (5) outside the study domain (that is, hemispheric sources). Imported (that is, non-national) O_3_ is associated with 88.3% of all attributable deaths, ranging from 83.0% in Italy to 100% in Liechtenstein. The greatest share of transported O_3_ had its origin outside the study domain, which was associated with 56.7% of total attributable mortality, ranging from 42.5% in Malta to 87.2% in Iceland. Moreover, imported O_3_ from the 34 other European countries also had a substantial attributable mortality impact (20.9%; intercountry range 5.1–40.0%) across the countries analyzed, whereas maritime transport contribution (7.2%; range 0–24.1%) was noticeable in smaller southern European countries such as Malta (24.1%) and Cyprus (14.0%). Results for the 813 regions are additionally provided in Supplementary Tables 4–6.

### Country-to-country O_3_-attributable mortality contribution

**Fig. 3:**
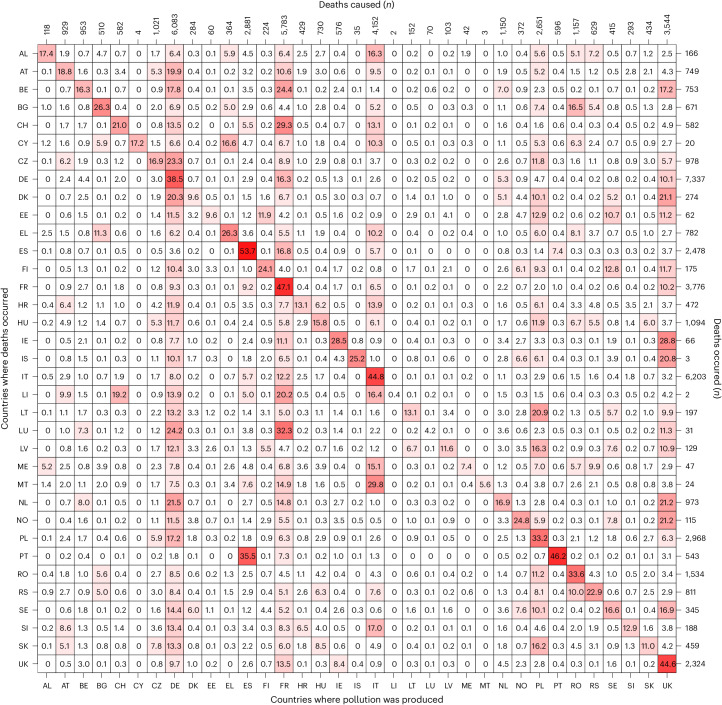
Country-to-country O_3_-attributable mortality contribution in Europe, 2015–2017. Each grid cell of the matrix shows the AF (%) in a country (row) due to O_3_ produced in another country (column). The diagonal represents mortality caused by national (that is, non-imported) O_3_. The number of attributable deaths that occurred in each country is shown on the right, and thus the sum of the grid cells in a row is always equal to 100% while the number of attributable deaths caused by each county is shown at the top. Country abbreviations used here are given in Methods.

The matrix of attributable mortality due to imported–exported O_3_ among the 35 European countries included in the analysis is depicted in Fig. 3. The most industrialized and populated countries are the major contributors to mortality attributable to transported transboundary O_3_, especially France (4,003 deaths during the warm season (May–September) of 2015–2017) and Germany (3,260 deaths). For instance, O_3_ originating from France has a substantial impact on mortality in neighboring countries including Luxembourg (32.3% of O_3_-attributable deaths), Switzerland (29.3%), Belgium (24.4%), Liechtenstein (20.2%), Spain (16.8%) and Germany (16.3%). O_3_ originating from Germany also substantially impacts mortality in neighboring countries including Luxembourg (24.2% of deaths), the Czech Republic (Czechia, 23.3%), the Netherlands (21.5%), Denmark (20.3%), Austria (19.9%), Belgium (17.8%) and Poland (17.2%).

**Fig. 4:**
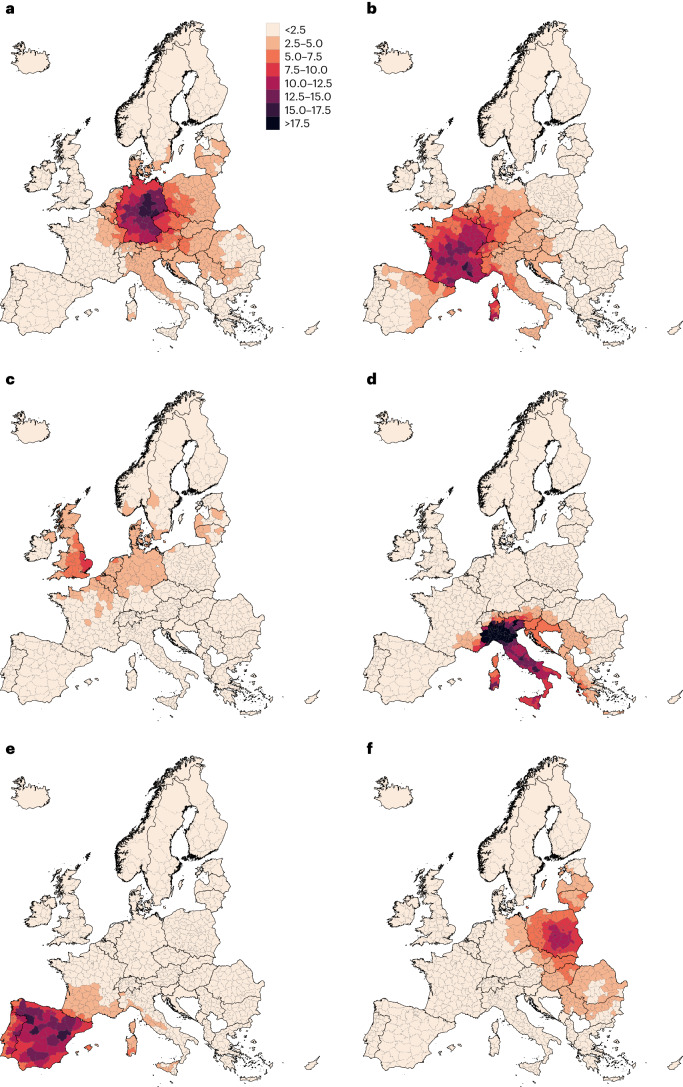
O_3_-attributable mortality caused by major O_3_ precursor-emitting countries, 2015–2017. **a**–**f**, Estimates are expressed as annual deaths per 1 million population: Germany (**a**); France (**b**); UK (**c**); Italy (**d**); Spain (**e**); and Poland (**f**).

**Fig. 5:**
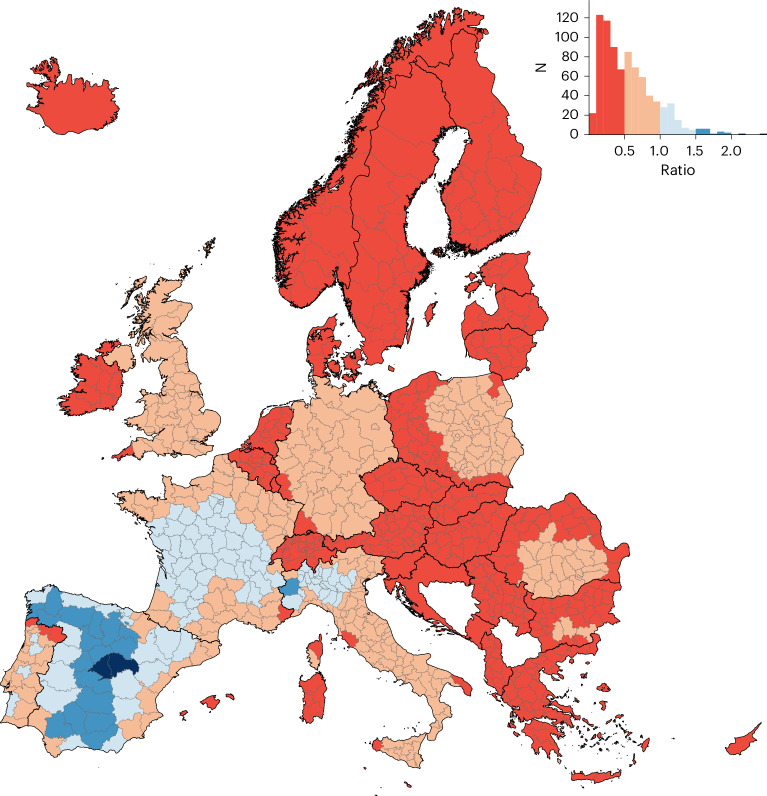
Ratio of national to imported O_3_-attributable deaths, 2015–2017. The histogram depicts both the color legend and the number of regions for each ratio, with a large number of regions where imported O_3_ deaths predominated over those nationally generated. The imported fraction refers to imports from the 34 other European countries.

Figure 4 shows regional maps of mortality caused by the major exporting countries, namely Germany, France, the UK, Italy, Spain and Poland. These maps emphasize the importance of the prevailing large-scale winds in the mid-latitudes (that is, the westerlies), which characterize an eastward plume of deaths attributable to transboundary-transported O_3_. Consequently, the south-western European countries are less affected by the health effects of transboundary-transported O_3_. Thus Spain (53.7%), France (47.1%) and Portugal (46.2%) are the countries with the largest attributable mortality due to national O_3_ production (Fig. 3) and the smallest imported:exported ratio of attributable deaths (Fig. 5).

### Sensitivity analysis

**Extended Data Fig. 1:**
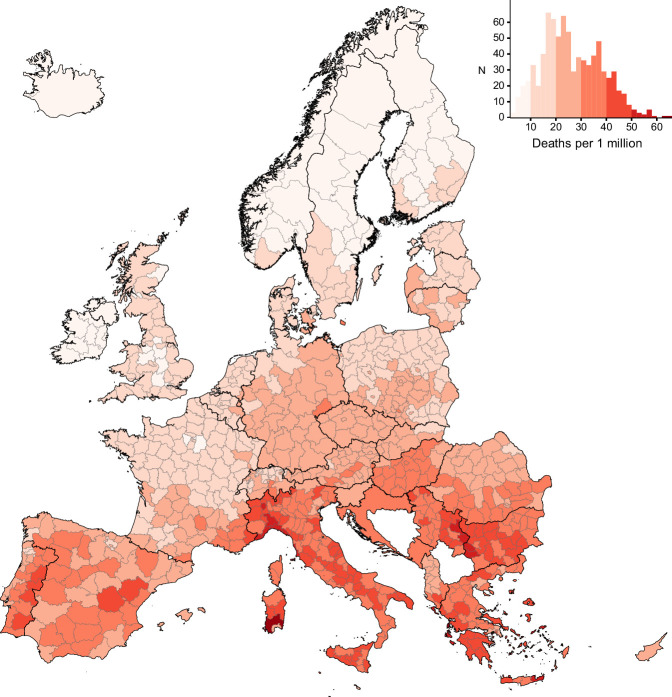
O_3_ attributable mortality rate during the warm season (May-September), 2015–2017. The mortality rate is expressed as annual deaths per million population. Only days with average 8-hour maximum O_3_ above 70 µg/m^3^ were considered in this analysis. The histogram depicts both the color legend and the number of regions for each value.

**Extended Data Fig. 2:**
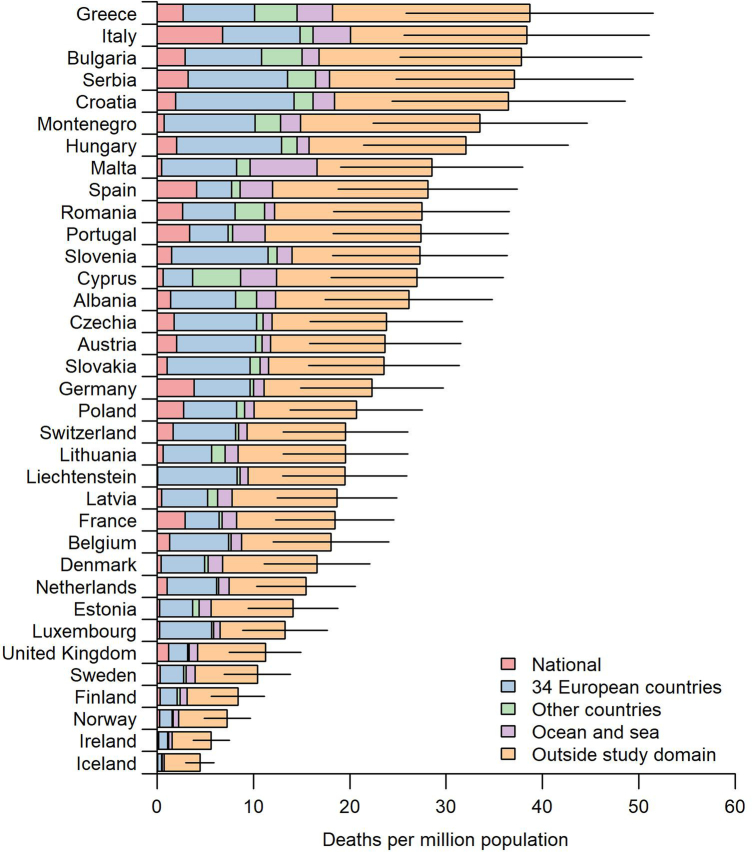
O_3_ associated mortality according to O_3_ emission sources in 35 European countries, 2015–2017. Only days with average mean 8-hour maximum O_3_ above 70 µg/m^3^ were considered in this analysis. The horizontal bars represent the 95% empirical CI of the overall O_3_ attributable mortality (ie, the sum of the five contribution sources).

**Extended Data Fig. 3:**
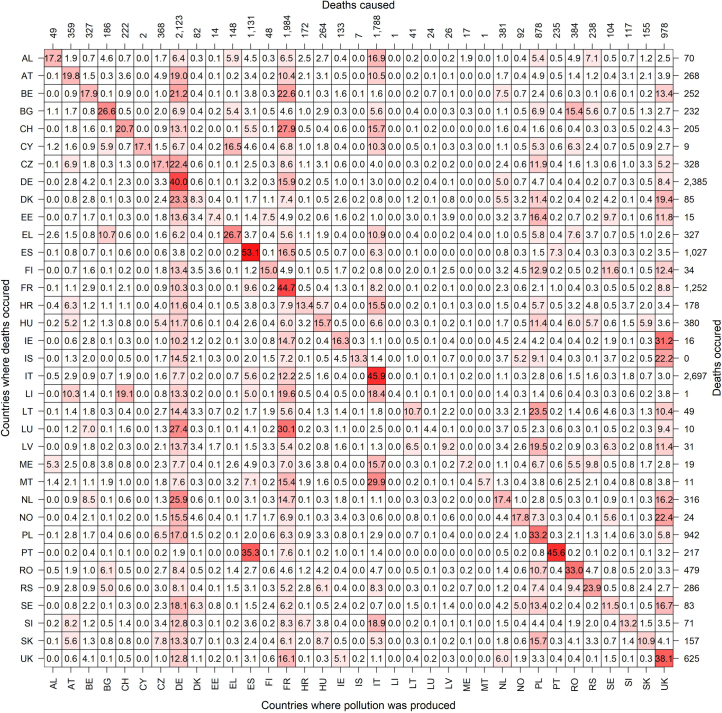
Country-to-country O_3_ attributable mortality contribution in Europe, 2015–2017. Only days with average 8-hour maximum O_3_ above 70 µg/m^3^ were considered in this analysis. Each grid cell of the matrix shows the AF (%) in a country (row) due to the O_3_ produced in another country (column). The diagonal corresponds to the mortality caused by the national (ie, non-imported) O_3_. The number of attributable deaths that occurred in each country is shown on the right, and thus the sum of the grid-cells in a row is always equal to 100%, while the number of attributable deaths caused by each county is shown on the top.

The mortality estimates reported are based on the whole range of O_3_ exposures. We performed a sensitivity analysis to evaluate the effect of a safe threshold at 70 µg m^−^^3^, in which (1) the exposure–response function between O_3_ and mortality was centered at 70 µg m^−3^ and (2) days when O_3_ concentrations were <70 µg m^−^^3^ were excluded (that is, days <70 µg m^−^^3^ are not harmful and health effects start to increase log-linearly beyond this threshold). Specifically, the number of deaths was reduced by a factor of nearly three, resulting in 36,523 (95% eCI 24,382–48,633) attributable deaths during the warm months (May–September) of 2015–2017 or, in other words, 23.0 (95% eCI 15.3–30.6) annual deaths per 1 million inhabitants (Extended Data Fig. 1). However, the apportionment of mortality according to O_3_-attributed sources (that is, countries) changed minimally (Extended Data Figs. 2 and 3).

## Discussion

This study presents an integrated, continent-wide analysis of mortality burden due to transported transboundary O_3_ in Europe. We found that only a small fraction of O_3_-attributable deaths was due to national sources (11.7%). The largest mortality burden was, instead, associated with hemispheric O_3_ transported from outside the European domain (56.7%). Moreover, the contribution of other European countries to each of the countries analyzed also had a substantial impact on mortality (20.9%). In some coastal regions and smaller countries in the Mediterranean, the contribution of maritime emissions on mortality was considerable.

The findings of our study have implications for air-quality and public health policies across Europe. Thus far, mitigation efforts have primarily focused on national and regional scales, lacking a comprehensive, transboundary assessment of pollution sources and their associated mortality. Future research should refine the present study by analyzing the contribution to mortality of the different economic sectors or activities, by country, to transported transboundary O_3_ (for example, energy, industry, transportation, residential and agriculture). This characterization of health impacts by type of emission would shed additional light on the interventions required in key strategic economic sectors to improve air quality and drive new health policies, which would need to be designed and implemented at the EU level and beyond in light of our results. The contribution of maritime emissions to O_3_ levels and associated attributable mortality in Mediterranean countries such as Malta, Greece and Cyprus, where their contribution is similar to or even larger than national contributions, indicates the need to implement a nitrogen emission control area to help reduce NO_x_ emissions, as previously done in the North Sea and Baltic Sea^15^. Given the large non-national contributions to average O_3_ in each location, our results should not be interpreted by local air-quality authorities as a justification for local inaction. Typically, during the highest O_3_ episodes, local contributions to O_3_ can increase substantially and local mitigation actions can contribute considerably to the reduction in instances where the 120 μg m^−^^3^ daily 8-h maximum target value and 180 μg m^−^^3^ hourly information threshold set by the EU Air Quality Directive have been exceeded^16^. In addition, local mitigation strategies are the key towards reducing the export of O_3_ to other regions and countries.

The present study highlights the need for a systematic quantification of national and remote contributions to air pollution concentrations, and their associated health impacts, as an essential step before the elaboration of mitigation plans, especially in regard to air pollutants such as O_3_ that are easily transported across borders. Our methodology allowed us to determine that the abatement strategies used to reduce O_3_ pollution, and the related health impacts, are to a large degree compromised by the long-range atmospheric transport of O_3_ and, in particular, hemispheric contributions. Nonetheless, in a globalized economy some of the imported pollution, and thus related mortality burden, has its origin in highly industrialized areas in Asia or North America. These goods and services, which are produced remotely but consumed in Europe, would need to be imputed to the respective European countries if we also accounted for the net import–export balance of goods, and not only that of air pollution concentrations^7,8^. Our study findings would help to implement coordinated pan-European and global strategies and thus achieve the air-quality guidelines of the WHO^6^ to avoid premature deaths and other health impacts such as hospitalizations and chronic diseases.

In this study we estimated a much higher premature mortality burden from O_3_ than that carried out by the EEA for the 35 countries analyzed. For example, in 2015 the EEA^17^ quantified an average of 32.6 deaths per 1 million population using annual data and assuming a higher mortality risk per 1 µg m^−^^3^ increase in O_3_. Instead, our study, based on warm-season data only (May–September), and assuming a lower mortality risk value, elevated this number to 74.5 deaths per 1 million inhabitants. This difference may, in part, be due to the fact that the EEA does not account for the potential health effects of ambient O_3_ <70 μg m^−^^3^, because when we adopt this assumption in our analysis we estimate a lower O_3_-attributable mortality rate (25.3 deaths per 1 million) than that reported by the EEA (32.6 deaths per 1 million). However, given that the current scientific evidence is insufficient to set a safe threshold (that is, a level below which O_3_ has no effect on mortality)^2,18^, the EEA data may be underestimating the actual impact of O_3_. Moreover, another methodological factor that might also contribute to differences in these estimates is that the EEA assumes a constant level of baseline mortality over each country while we use the actual number of deaths in each European region.

One of the strengths of this study is the use of a unique and format-homogeneous mortality dataset for 813 contiguous regions in 35 European countries, including deaths in both urban and rural areas. The large geographical coverage and high spatial resolution of our dataset allowed detailed characterization of the effects of O_3_ on all-cause mortality across the whole continent. Another strength of this study is the use of a source-apportionment method to quantify the contributions of O_3_ from specific geographic locations.

However, our study also had some limitations that must be acknowledged. First, its estimates consider only acute effects on mortality but the possibility of chronic effects cannot be ruled out despite the inconsistent evidence to date^19,20^. Second, as is common in large-scale health-impact assessments^7,8,12^, we assumed a fixed association between O_3_ and mortality for all countries, given that country- or region-specific associations were not yet available. The risk of death from O_3_ might, however, vary markedly between one population and another depending on underlying health, demographic and socioeconomic characteristics, which is a potential source of bias for reported estimates. Third, we did not consider the years of life lost (YLL) as an additional health outcome because our mortality data were not available according to individual age. Note that the calculation of YLL involves totaling deaths occurring at each individual age and multiplying this by the number of remaining years of life up to a selected age limit (usually life expectancy). Moreover, short-term exposure-response functions between O_3_ and YLL reported in the literature were based on Chinese data only and therefore they were not representative of the European context. Fourth, we were not able to estimate the economic cost associated with O_3_-attributable deaths. Fifth, we were not able to propagate the uncertainty of the air pollution models to health impacts^21^. Finally, while source-apportionment methods avoid the limitations associated with typical sensitivity analysis methods^22^, these are not devoid of uncertainties. Future studies may attempt to apply other source-apportionment schemes with each having its own specificities and limitations^23^.

Climate warming will reinforce conditions conducive to the formation of tropospheric O_3_ in the future, because the photochemical mechanisms of O_3_ formation are favored during heatwaves and periods of high solar radiation. This is indeed the dominant factor leading to the projected increase in O_3_ concentrations that might aggravate the associated health impacts^24^. However, the effect of climate warming is not limited to O_3_ formation—it also influences the emissions of chemical compounds that are O_3_ precursors, such as VOCs of biotic origin (that is, emitted by vegetation), which could counteract the efforts devoted to the reduction of the emissions of anthropogenic NO_x_ and VOCs. Therefore, the fight against climate change is key to improvement in air quality and, in turn, a key element to consider in future studies for the design and implementation of long-term, long-lasting policies to be discussed at the continental, hemispheric and global scales.

In conclusion, achieving the air-quality guidelines set out by the WHO and avoiding premature deaths and other types of health impacts of O_3_ require not only the implementation of national or coordinated pan-European actions, but also global strategies. Quantitative approaches directly linking local health impacts to O_3_ contributions from different geographical regions are key to the design and implementation of such coordinated and tailored mitigation strategies.

## Methods

To develop our methodological framework for this large-scale health-impact assessment we combined data on O_3_ concentrations, population numbers and mortality records, together with the exposure–response associations modeling the effects of O_3_ on human mortality.

### Health data

Weekly time series of all-cause mortality counts^25^ for the warm season (weeks 18–39 (ISO8601)), corresponding approximately to the months May–September, together with annual population estimates^26^, were obtained for the period 2015–2017 from Eurostat. The mortality dataset included 6,291,008 counts of death from 813 contiguous regions (nomenclature of territorial units for statistics 1–3) representing about 530 million people in 35 countries: Albania (AL, *n* = 12 regions), Austria (AT, *n* = 35), Belgium (BE, *n* = 11), Bulgaria (BG, *n* = 28), Croatia (HR, *n* = 1), Cyprus (CY, *n* = 1), Czechia (CZ, *n* = 14), Denmark (DK, *n* = 11), Estonia (EE, *n* = 5), Finland (FI, *n* = 19), France (FR, *n* = 96), Germany (DE, *n* = 38), Greece (EL, *n* = 50), Hungary (HU, *n* = 20), Iceland (IS, *n* = 2), Ireland (IE, *n* = 8), Italy (IT, *n* = 107), Latvia (LV, *n* = 6), Liechtenstein (LI, *n* = 1), Lithuania (LT, *n* = 10), Luxembourg (LU, *n* = 1), Malta (MT, *n* = 1), Montenegro (ME, *n* = 1), the Netherlands (NL, *n* = 12), Norway (NO, *n* = 11), Poland (PL, *n* = 73), Portugal (PT, *n* = 23), Romania (RO, *n* = 42), Serbia (RS, *n* = 25), Slovakia (SK, *n* = 8), Slovenia (SI, *n* = 1), Spain (ES, *n* = 50), Sweden (SE, *n* = 21), Switzerland (CH, *n* = 26) and UK (*n* = 41).

### Air-quality modeling

**Extended Data Fig. 4:**
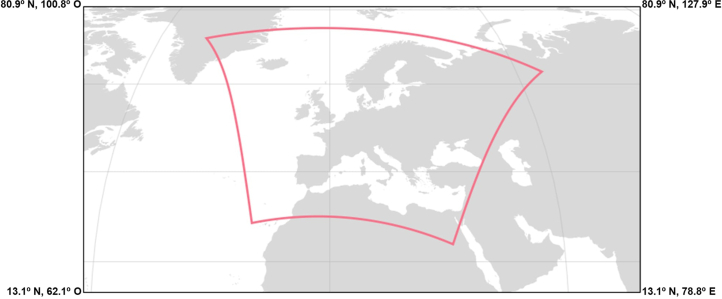
Map of the study domain. The study domain includes countries other than the 35 analysed countries (non-EU-35) along with the ocean and the sea, where both land and maritime emissions occur.

The modeling experiment used to track O_3_ and its precursor emissions is based on the CALIOPE air-quality system^27,28^ developed at Barcelona Supercomputing Center (BSC). The system is run at a horizontal grid of 18 km^2^ covering Europe and surrounding areas (Extended Data Fig. 4), and it integrates several components: the WRF-ARW (v.3.6) meteorological model^29^, the HERMES (v.3) anthropogenic emissions model^30^, the MEGAN (v.2.0.4) biogenic emissions model^31^ and the CMAQ (v.5.0.2) chemical transport model^32^. The WRF-ARW model is fed by meteorological initial and boundary conditions from ERA-Interim^33^ atmospheric reanalysis; the HERMES model uses regional anthropogenic emission data from CAMS-REG-AP, v.4.2 (ref. ^34^); and the CMAQ model takes chemical boundary conditions (reactive gases and aerosols data) from the CAMS global analysis^35^. The MEGAN model uses temperature and solar radiation from the WRF-ARW and specified soil emission factors, plant leaf area index and plant functional type to derive biogenic emissions.

### Source apportionment of O_3_

Ozone is a secondary pollutant and currently there is a lack of observational methods available to discern its sources. However, chemical transport models, while characterized by inherent uncertainties, offer a valuable means to attribute O_3_ concentration contributions to specific sources, be it by activity sector (for example, traffic or industry) or geographical region. The predominant approach employed for such attribution is often referred to as the ‘brute force’ method, which involves conducting a series of simulations where individual sources are systematically reduced or deactivated, followed by a comparative analysis against a baseline simulation encompassing all sources. However, brute force is not suitable for retrieval of source contributions when the relationship between emissions and concentrations is nonlinear, as in the case of O_3_. A second approach involves tracking pollutants throughout their lifetime using a tagging method within the chemical transport models. This method quantifies the contribution of a sector or region to the total concentration of a pollutant while accounting for nonlinear processes and mass conservation, aspects that provide more realistic estimates compared with the brute force method. Here we used the tagging approach within a regional air-quality modeling system to quantify the contributions to ground-level O_3_ within 35 European countries, covering 813 contiguous regions with access to high-quality mortality data (listed above). The tagging approach tracks both O_3_ and its precursors (NO_x_ and VOCs) formed or emitted in each region all the way through their life cycle in the atmosphere, including transport, chemistry and deposition. Our study primarily focused on assessment of both national and imported O_3_ contributions for each country. The imported contributions for each country were further classified based on their origin, including the individual contributions from the other European countries considered in the analysis, neighboring countries, maritime sources primarily associated with shipping in close proximity to the European continent, and hemispheric sources.

**Extended Data Fig. 5:**
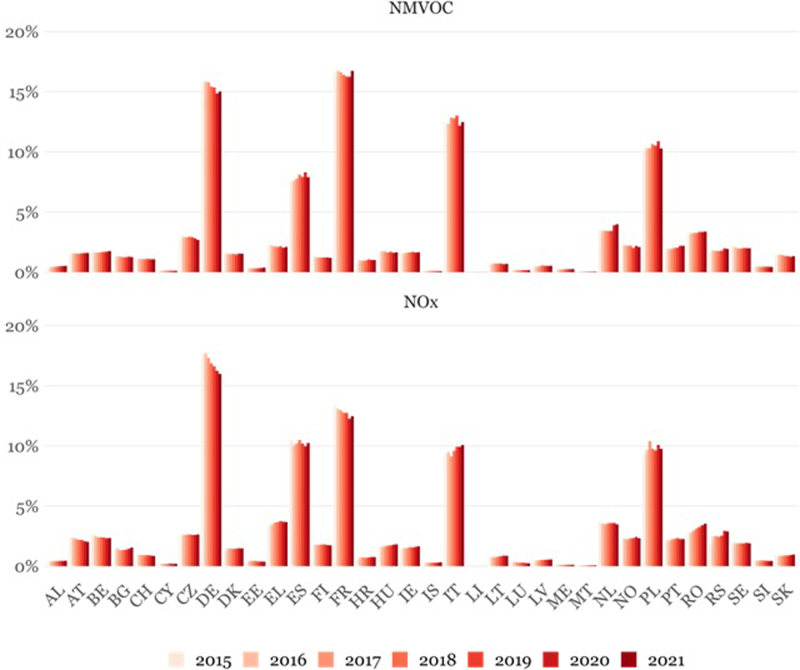
National contributions to total annual NO_x_ and NMVOC anthropogenic emissions by country, 2015–2021. Reference: Centre on Emissions Inventories and Projections. Officially reported emission data [https://www.ceip.at/webdab-emission-database/reported-emissiondata (last access: September 2023).

The contribution to ground-level O_3_ (daily maximum 8-h average) from each of the 35 countries in each location was obtained using the integrated source-apportionment method implemented within CMAQ in the CALIOPE system. The CMAQ–integrated source-apportionment methodology is described in detail elsewhere^36,37^. The source-apportionment method used here assumes that the formation of O_3_ within each location is governed by either a NO_x_- or VOC-limited chemical regime. This entails assigning all production to the precursor that acts as the limiting factor. Depending on the regime, NO_x_ or VOC tracers associated with each tagged source are proportionally attributed to the O_3_ formed, and vice versa when O_3_ is transformed into other species. Determination of the chemical regime that controls O_3_ production is made according to the ratio H_2_O_2_:HNO_3_, where a ratio <0.35 designates a VOC-sensitive regime, and a ratio >0.35 a NO_x_-sensitive regime^38^. The simulation domain (Extended Data Fig. 4) also includes countries other than the 35 analyzed (non-EU-35), along with ocean and sea, where both land and maritime emissions occur. The method also provides the separate contribution of these two emission sources to O_3_ concentrations along with the O_3_ contribution transported through the simulation domain boundaries, which mostly accounts for remote hemispheric O_3_ contributions. The modeling experiment covered the extended summer season (May–September) for the years 2015–2017, including 3 years of increases, with the robustness of our results demonstrated by their accounting for interannual variability in meteorological conditions. The selection of these 3 years is limited to the availability of a consistent regional emission inventory (CAMS-REG-AP, v.4.2). CAMS-REG-AP, v.4.2 is primarily based on official national inventories reported with a 2-year time lag. The lagged reporting deadlines, together with the time needed to produce the CAMS-REG-AP inventory, implies that the final product typically presents a time lag of 3–4 years from the present time. The contribution of the 35 countries to total NO_x_ and VOC anthropogenic emissions during the years 2015–2017 has remained consistent when compared with more recent years (2018–2021), and therefore the selected years can be considered representative of more recent emission shares when modeling imported and national O_3_ contributions by country (Extended Data Fig. 5).

### Statistical analysis

#### Health-impact assessment

The effect of O_3_ on mortality was derived from the largest available multicountry epidemiological study to date^2^, which reported a statistically significant, meta-analytic coefficient (*β*) of the log-linear exposure–response association between O_3_ and all-cause mortality of 0.00018 (95% CI 0.00012–0.00024) per 1 µg m^−^^3^ increase in daily maximum 8-h average O_3_. Sensitivity analyses suggested no evidence of nonlinearity in the exposure–response association^2^.

The health-impact assessment consisted of the following steps. First, for each grid cell (*x*) and day (*d*) of the model simulations we transformed the daily maximum 8-h average O_3_ (μg m^−^^3^) into the daily mortality attributable fraction (AF(*x*,*d*)) according to

$${{\mathrm{AF}}}\left(x,d\right)=1-\exp (-\beta \times {{\mathrm{O}}}_{3}(x,d)),$$

where *β* is the mortality risk per unit increase in O_3_ mentioned above^39^. We used the entire range of O_3_ because there is no compelling evidence of a so-called safe threshold^2,18^. However, we performed a sensitivity analysis to evaluate the effect of a safe threshold at 70 µg m^−^^3^, excluding days with O_3_ <70 µg m^−^^3^ and centering the association at 70 µg m^−^^3^, as commonly done in previous assessments^2,13,14^, including those by the EEA^4^. Second, for each grid cell and week (*w*) we calculated the weekly averages of AF (*x*,*w*). Third, for each European region (*r*) and week we computed the population-weighted average of AF by considering all continental grid cells of the region, using gridded population counts at a horizontal 1-km^2^ resolution for the year 2015 from the Global Human Settlement Layer^40^. We note that in the boundary grid cells (that is, those representing more than one region), the population considered was the only one of the corresponding region. Fourth, for each region we transformed the weekly time series of population-weighted AF into a weekly time series of mortality attributable number (AN) according to

$${{\mathrm{AN}}}(r,w)=N(r,w)\times {{\mathrm{AF}}}(r,w),$$

where *N*(*r*,*w*) is the total number of deaths in the corresponding region *r* and week *w*. The weekly time series of AN were apportioned according to O_3_-attributed sources to the AF in each region. Moreover, we estimated the 95% CI of weekly AN by assuming the upper and lower range of the *β* coefficient of the log-linear exposure–response association between O_3_ and mortality. Finally, total AN during the warm season (May–September) resulted from the sum of the weekly contributions, and its ratio with the total population provided the O_3_-attributable mortality rate.

#### Evaluation of O_3_ simulations

**Extended Data Fig. 6:**
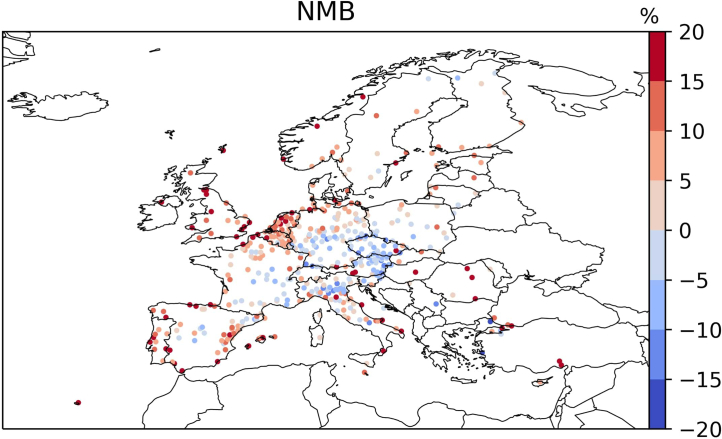
Normalized Mean Bias (NMB) for the O_3_ concentrations for the weeks 18–39 in 2015–2017. EEA rural background stations below 1 000 m above sea level.

We used hourly observations of ground-level O_3_ concentrations from the Air Quality eReporting database of the EEA to evaluate the quality of the modeled O_3_ (overall daily maximum 8-h average) values by CALIOPE. Specifically, we compared the modeled O_3_ concentrations with observations provided by the EEA for rural background stations <1,000 m above sea level, because the spatial representativeness of these stations is comparable to CALIOPE spatial resolution. Only those days with >75% availability of hourly observations were used to calculate the observed O_3_ and compare it with the modeled values. The results of the evaluation showed good agreement between modeled and observed O_3_ concentrations (Supplementary Table 7), with a Pearson correlation coefficient of 0.66 ± 0.11, a normalized mean bias of 5.49 ± 9.25% and a normalized root mean squared error of 20.46 ± 5.7. Extended Data Fig. 6 shows the normalized mean bias in each of the EEA stations used.

In addition to the traditional statistical indicators used for evaluation of model performance, we followed the Guidance Document on Modelling Quality Objectives and Benchmarking^41^ established by the Forum for Air Quality Modeling, which aims to promote and support the harmonized use of models and their applications under the European Air Quality Directive (no. 2008/50/EC). The Forum for Air Quality Modeling proposes a modeling quality indicator (MQI) to assess the reliability of air-quality models considering the measurement uncertainty at each individual station. The MQI is defined as

$${\mathrm{MQI}}={\mathrm{r}}.{\mathrm{m}}.{\mathrm{s}}.{\mathrm{e}}./(\,\beta \times {\mathrm{r}}.{\mathrm{m}}.{\mathrm{s}}._{\mathrm{U}}),$$

where r.m.s.e. is the root mean squared error between the modeled and observed pollutant concentrations, r.m.s._U_ is pollutant measurement uncertainty and *β* is set to 2, allowing the difference between the modeled and observed concentration to be twice the measurement uncertainty. MQI ≤ 1 indicates that the model error at a specific station is acceptable compared with the uncertainty of observations. When 90% of the individual air-quality stations included in the model evaluation assessment have MQI ≤ 1, the air-quality model meets the modeling quality objective and can be considered reliable for air-quality assessment within the framework of the European Air Quality Directive. The model used in this study complied with MQI ≤ 1 in 100% of rural background stations <1,000 m above sea level in the EEA dataset (Supplementary Table 8).

### Reporting summary

Further information on research design is available in the Nature Portfolio Reporting Summary linked to this article.

## Online content

Any methods, additional references, Nature Portfolio reporting summaries, source data, extended data, supplementary information, acknowledgements, peer review information; details of author contributions and competing interests; and statements of data and code availability are available at 10.1038/s41591-024-02976-x.

### Supplementary information

Supplementary Information Supplementary Tables 1–8.

Reporting Summary

## Data Availability

Weekly deaths and population numbers can be freely downloaded from Eurostat (https://ec.europa.eu/eurostat/cache/metadata/en/demomwk_esms.htm; https://ec.europa.eu/eurostat/cache/metadata/en/demo_r_gind3_esms.htm), and O_3_ simulated values from the Zenodo repository (10.5281/zenodo.10606147)^42^.

## Code availability

The CMAQ code is available at https://github.com/USEPA/CMAQ, the WRF-ARW code at https://github.com/wrf-model/WRF, the HERMESv3 code at https://earth.bsc.es/gitlab/es/hermesv3_gr and the MEGAN code at https://bai.ess.uci.edu/megan/data-and-code/.
