# Supplementary material for: Geographic sources of ozone air pollution and mortality burden in Europe
Source: Nat Med. 2024 Jun 3;30(6):1732–8. doi: 10.1038/s41591-024-02976-x (PMC11186783; doi:10.1038/s41591-024-02976-x)
Supplement: Supplementary file 1 — Supplementary Tables 1–8. [file 41591_2024_2976_MOESM1_ESM.pdf]

nature medicine

Supplementary information https://doi.org/10.1038/s41591-024-02976-x

Geographic sources of ozone air pollution and mortality burden in Europe

In the format provided by the authors and unedited

Supplementary Information

Table S1. O_3 levels according to O_3 precursors emission sources in 35 European countries, 2015-2017 2
Table S2. O_3 attributable deaths according to O_3 precursors emission sources in 35 European countries, 2015-2017 3
Table S3. Average annual O_3 attributable mortality rate according to O_3 emission sources in 35 European countries, 2015-2017 ... 4
Table S4. O_3 levels according to O_3 precursors emission sources in European regions, 2015-2017 5
Table S5. O_3 attributable deaths according to O_3 precursors emission sources in European regions, 2015-2017 26
Table S6. Average annual O_3 attributable mortality rate according to O_3 emission sources in European regions, 2015-2017 47
Table S7. Statistics for O_3 concentrations for all the tagged countries for the weeks 18-39 of 2015-2017 68
Table S8. EEA stations by country and year with Modelling Quality Indicator (MQI) ≤ 1 70

Table S1. O_3 levels according to O_3 precursors emission sources in 35 European countries, 2015-2017

	Ozone (μg/m³)						Percentage (%)					
Country	National	34 European countries	Other countries	Ocean and sea	Outside study domain	Overall	National	34 European countries	Other countries	Ocean and sea	Outside study domain	Overall
Albania	5.6	30.8	10.3	8.7	65.3	120.7	4.6	25.5	8.5	7.2	54.1	100.0
Austria	7	36.7	2.8	4.4	57.5	108.4	6.5	33.9	2.6	4.1	53.0	100.0
Belgium	5.3	28.3	1.1	6.3	55.3	96.3	5.5	29.4	1.1	6.5	57.4	100.0
Bulgaria	7	22.3	12.3	4.8	59.2	105.6	6.6	21.1	11.6	4.5	56.1	100.0
Croatia	4.6	36.7	6.5	7.1	56.9	111.8	4.1	32.8	5.8	6.4	50.9	100.0
Cyprus	2.6	14.8	24	18.4	69.4	129.2	2.0	11.5	18.6	14.2	53.7	100.0
Czechia	6.7	35.2	2.8	4.3	53	102.0	6.6	34.5	2.7	4.2	52.0	100.0
Denmark	2.4	22.7	2.4	8.9	59.9	96.3	2.5	23.6	2.5	9.2	62.2	100.0
Estonia	1.8	17.5	4.7	7.2	54.3	85.5	2.1	20.5	5.5	8.4	63.5	100.0
Finland	3.4	10.9	3.2	5.3	53.9	76.7	4.4	14.2	4.2	6.9	70.3	100.0
France	14.5	16.3	1.3	7.4	59.4	98.9	14.7	16.5	1.3	7.5	60.1	100.0
Germany	14.6	23.6	1.6	5.3	53	98.1	14.9	24.1	1.6	5.4	54.0	100.0
Greece	6.8	24.1	13.5	11.9	64.4	120.7	5.6	20.0	11.2	9.9	53.4	100.0
Hungary	5.8	34.6	5.6	4.2	53.6	103.8	5.6	33.3	5.4	4.0	51.6	100.0
Iceland	1.1	4	0.5	3.1	68.9	77.6	1.4	5.2	0.6	4.0	88.8	100.0
Ireland	2.9	7.3	0.6	4.5	66.7	82.0	3.5	8.9	0.7	5.5	81.3	100.0
Italy	18.4	26	4.4	11.8	60.6	121.2	15.2	21.5	3.6	9.7	50.0	100.0
Latvia	2.3	18.6	5.5	6.6	52.8	85.8	2.7	21.7	6.4	7.7	61.5	100.0
Liechtenstein	0.2	44.8	1.8	4.7	60.3	111.8	0.2	40.1	1.6	4.2	53.9	100.0
Lithuania	2.7	20.1	6.6	6.2	52.5	88.1	3.1	22.8	7.5	7.0	59.6	100.0
Luxembourg	1.5	35.2	1.2	5.3	54.2	97.4	1.5	36.1	1.2	5.4	55.6	100.0
Malta	2.1	35	6.7	31.4	54.9	130.1	1.6	26.9	5.1	24.1	42.2	100.0
Montenegro	2.1	32.4	9	6.7	66.1	116.3	1.8	27.9	7.7	5.8	56.8	100.0
Netherlands	5.2	26.1	1.2	7	55.4	94.9	5.5	27.5	1.3	7.4	58.4	100.0
Norway	3.1	10.4	1.6	6	63.7	84.8	3.7	12.3	1.9	7.1	75.1	100.0
Poland	11.5	23.6	4.6	5.1	52.5	97.3	11.8	24.3	4.7	5.2	54.0	100.0
Portugal	12	14.8	1.6	12.1	68.7	109.2	11.0	13.6	1.5	11.1	62.9	100.0
Romania	9.1	19.3	11.5	3.7	56.7	100.3	9.1	19.2	11.5	3.7	56.5	100.0
Serbia	7.5	29.6	8.5	4.3	56.4	106.3	7.1	27.8	8.0	4.0	53.1	100.0
Slovakia	4.3	36.7	5.1	4.1	54	104.2	4.1	35.2	4.9	3.9	51.8	100.0
Slovenia	5.2	40.8	3.9	6.2	56.2	112.3	4.6	36.3	3.5	5.5	50.0	100.0
Spain	15.2	13.2	3	11.8	70.3	113.5	13.4	11.6	2.6	10.4	61.9	100.0
Sweden	2.8	14.3	2.3	6.7	56.8	82.9	3.4	17.2	2.8	8.1	68.5	100.0
Switzerland	8.3	34.2	1.7	4.9	58.8	107.9	7.7	31.7	1.6	4.5	54.5	100.0
United Kingdom	8.1	10.2	0.8	6.2	61.6	86.9	9.3	11.7	0.9	7.1	70.9	100.0
Europe	6.1	24.3	5.0	7.5	58.9	101.9	6.0	23.8	4.9	7.4	57.8	100.0

Table S2. O_3 attributable deaths according to O_3 precursors emission sources in 35 European countries, 2015-2017

Country	Number of deaths						Percentage (%)					
	National	34 European countries	Other countries	Ocean and sea	Outside study domain	Overall (95% eCI)	National	34 European countries	Other countries	Ocean and sea	Outside study domain	Overall
Albania	29	137	45	41	286	538 (359 to 713)	5.4	25.5	8.4	7.6	53.2	100.0
Austria	141	608	50	71	918	1,788 (1,196 to 2,376)	7.9	34.0	2.8	4.0	51.3	100.0
Belgium	123	631	25	144	1,254	2,177 (1,455 to 2,893)	5.6	29.0	1.1	6.6	57.6	100.0
Bulgaria	177	495	272	107	1,333	2,384 (1,595 to 3,169)	7.4	20.8	11.4	4.5	55.9	100.0
Croatia	62	411	67	71	620	1,231 (823 to 1,636)	5.0	33.4	5.4	5.8	50.4	100.0
Cyprus	3	17	28	21	81	150 (101 to 200)	2.0	11.3	18.7	14.0	54.0	100.0
Czechia	165	813	69	99	1,224	2,370 (1,585 to 3,150)	7.0	34.3	2.9	4.2	51.6	100.0
Denmark	26	248	26	97	669	1,066 (713 to 1,417)	2.4	23.3	2.4	9.1	62.8	100.0
Estonia	6	56	15	23	178	278 (186 to 370)	2.2	20.1	5.4	8.3	64.0	100.0
Finland	42	133	38	64	614	891 (596 to 1,186)	4.7	14.9	4.3	7.2	68.9	100.0
France	1,780	1,996	157	945	7,124	12,002 (8,026 to 15,953)	14.8	16.6	1.3	7.9	59.4	100.0
Germany	2,823	4,514	296	1,011	10,136	18,780 (12,558 to 24,963)	15.0	24.0	1.6	5.4	54.0	100.0
Greece	206	576	347	273	1,627	3,029 (2,026 to 4,024)	6.8	19.0	11.5	9.0	53.7	100.0
Hungary	172	922	155	111	1,450	2,810 (1,879 to 3,734)	6.1	32.8	5.5	4.0	51.6	100.0
Iceland	1	2	0	2	34	39 (25 to 51)	2.6	5.1	0.0	5.1	87.2	100.0
Ireland	19	47	4	28	409	507 (339 to 674)	3.7	9.3	0.8	5.5	80.7	100.0
Italy	2,782	3,421	582	1,621	7,920	16,326 (10,924 to 21,687)	17.0	21.0	3.6	9.9	48.5	100.0
Latvia	15	114	33	40	322	524 (350 to 697)	2.9	21.8	6.3	7.6	61.5	100.0
Liechtenstein	0	2	0	0	3	5 (4 to 8)	0.0	40.0	0.0	0.0	60.0	100.0
Lithuania	26	171	60	52	454	763 (510 to 1014)	3.4	22.4	7.9	6.8	59.5	100.0
Luxembourg	1	30	1	4	46	82 (55 to 109)	1.2	36.6	1.2	4.9	56.1	100.0
Malta	1	23	5	21	37	87 (58 to 115)	1.1	26.4	5.7	24.1	42.5	100.0
Montenegro	3	43	12	9	87	154 (104 to 207)	1.9	27.9	7.8	5.8	56.5	100.0
Netherlands	165	808	36	208	1,707	2,924 (1,955 to 3,887)	5.6	27.6	1.2	7.1	58.4	100.0
Norway	29	87	13	50	484	663 (442 to 879)	4.4	13.1	2.0	7.5	73.0	100.0
Poland	987	1,981	388	408	4,349	8,113 (5,425 to 10,785)	12.2	24.4	4.8	5.0	53.6	100.0
Portugal	251	292	31	275	1,407	2,256 (1,509 to 2,997)	11.1	12.9	1.4	12.2	62.4	100.0
Romania	515	1,019	627	198	3,009	5,368 (3,590 to 7,136)	9.6	19.0	11.7	3.7	56.1	100.0
Serbia	186	626	179	90	1,183	2,264 (1,513 to 3,007)	8.2	27.7	7.9	4.0	52.3	100.0
Slovakia	50	408	59	46	604	1,167 (781 to 1,552)	4.3	35.0	5.1	3.9	51.8	100.0
Slovenia	24	163	16	25	226	454 (304 to 603)	5.3	35.9	3.5	5.5	49.8	100.0
Spain	1,330	1,148	261	1,093	5,619	9,451 (6,323 to 12,557)	14.1	12.1	2.8	11.6	59.5	100.0
Sweden	57	288	43	132	1,061	1,581 (1,057 to 2,103)	3.6	18.2	2.7	8.3	67.1	100.0
Switzerland	122	460	22	67	782	1,453 (972 to 1,931)	8.4	31.7	1.5	4.6	53.8	100.0
United Kingdom	1,037	1,286	104	764	7,581	10,772 (7,201 to 14,325)	9.6	11.9	1.0	7.1	70.4	100.0
Europe	13,356	23,976	4,066	8,211	64,838	114,447 (76,539 to 152,108)	11.7	20.9	3.6	7.2	56.7	100.0

Table S3. Average annual O_3 attributable mortality rate according to O_3 emission sources in 35 European countries, 2015-2017

Country	Population	Number of deaths per million population					
		National	34 European countries	Other countries	Ocean and sea	Outside study domain	Overall (95% eCI)
Albania	2,877,078	3.36	15.87	5.21	4.75	33.14	62.33 (41.59 to 82.61)
Austria	8,720,137	5.39	23.24	1.91	2.71	35.09	68.35 (45.72 to 90.82)
Belgium	11,324,677	3.62	18.57	0.74	4.24	36.91	64.08 (42.83 to 85.15)
Bulgaria	7,126,973	8.28	23.15	12.72	5.00	62.35	111.5 (74.6 to 148.22)
Croatia	4,168,923	4.96	32.86	5.36	5.68	49.57	98.43 (65.8 to 130.81)
Cyprus	853,591	1.17	6.64	10.93	8.20	31.63	58.58 (39.44 to 78.1)
Czechia	10,570,249	5.20	25.64	2.18	3.12	38.60	74.74 (49.98 to 99.34)
Denmark	5,724,233	1.51	14.44	1.51	5.65	38.96	62.08 (41.52 to 82.51)
Estonia	1,316,396	1.52	14.18	3.80	5.82	45.07	70.39 (47.1 to 93.69)
Finland	5,493,873	2.55	8.07	2.31	3.88	37.25	54.06 (36.16 to 71.96)
France	64,563,213	9.19	10.31	0.81	4.88	36.78	61.97 (41.44 to 82.36)
Germany	82,171,811	11.45	18.31	1.20	4.10	41.12	76.18 (50.94 to 101.26)
Greece	10,787,789	6.37	17.80	10.72	8.44	50.27	93.59 (62.6 to 124.34)
Hungary	9,815,498	5.84	31.31	5.26	3.77	49.24	95.43 (63.81 to 126.81)
Iceland	337,107	0.99	1.98	0.00	1.98	33.62	38.56 (24.72 to 50.43)
Ireland	4,754,674	1.33	3.30	0.28	1.96	28.67	35.54 (23.77 to 47.25)
Italy	60,633,659	15.29	18.81	3.20	8.91	43.54	89.75 (60.05 to 119.22)
Latvia	1,959,888	2.55	19.39	5.61	6.80	54.77	89.12 (59.53 to 118.54)
Liechtenstein	37,728	0.00	17.67	0.00	0.00	26.51	44.18 (35.34 to 70.68)
Lithuania	2,866,657	3.02	19.88	6.98	6.05	52.79	88.72 (59.3 to 117.91)
Luxembourg	582,970	0.57	17.15	0.57	2.29	26.30	46.89 (31.45 to 62.32)
Malta	456,526	0.73	16.79	3.65	15.33	27.02	63.52 (42.35 to 83.97)
Montenegro	622,266	1.61	23.03	6.43	4.82	46.60	82.49 (55.71 to 110.89)
Netherlands	17,035,609	3.23	15.81	0.70	4.07	33.40	57.21 (38.25 to 76.06)
Norway	5,232,789	1.85	5.54	0.83	3.19	30.83	42.23 (28.16 to 55.99)
Poland	37,980,626	8.66	17.39	3.41	3.58	38.17	71.2 (47.61 to 94.65)
Portugal	9,827,786	8.51	9.90	1.05	9.33	47.72	76.52 (51.18 to 101.65)
Romania	19,702,173	8.71	17.24	10.61	3.35	50.91	90.82 (60.74 to 120.73)
Serbia	7,020,865	8.83	29.72	8.50	4.27	56.17	107.49 (71.83 to 142.76)
Slovakia	5,431,517	3.07	25.04	3.62	2.82	37.07	71.62 (47.93 to 95.25)
Slovenia	2,064,959	3.87	26.31	2.58	4.04	36.48	73.29 (49.07 to 97.34)
Spain	44,201,053	10.03	8.66	1.97	8.24	42.37	71.27 (47.68 to 94.7)
Sweden	9,928,446	1.91	9.67	1.44	4.43	35.62	53.08 (35.49 to 70.61)
Switzerland	8,367,121	4.86	18.33	0.88	2.67	31.15	57.89 (38.72 to 76.93)
United Kingdom	65,587,543	5.27	6.54	0.53	3.88	38.53	54.75 (36.6 to 72.8)
Europe	530,146,403	8.40	15.08	2.56	5.16	40.77	71.96 (48.12 to 95.64)

Table S4. O_3 levels according to O_3 precursors emission sources in European regions, 2015-2017
NUTS ID represents the code of the region.

		Ozone (μg/m^3)						Percentage (%)					
Country	NUTS ID	National	34 European countries	Other countries	Ocean and sea	Outside study domain	Overall	National	34 European countries	Other countries	Ocean and sea	Outside study domain	Overall
Albania	AL011	5.3	29.4	11.0	7.0	68.1	120.8	4.4	24.3	9.1	5.8	56.4	100.0
Albania	AL012	7.0	30.9	9.9	9.5	61.2	118.5	5.9	26.1	8.4	8.0	51.6	100.0
Albania	AL013	3.2	31.0	13.2	6.2	64.8	118.4	2.7	26.2	11.1	5.2	54.7	100.0
Albania	AL014	5.6	31.1	11.3	8.0	63.3	119.3	4.7	26.1	9.5	6.7	53.1	100.0
Albania	AL015	3.7	32.6	11.3	7.5	64.3	119.4	3.1	27.3	9.5	6.3	53.9	100.0
Albania	AL021	6.8	29.2	10.0	7.9	66.4	120.3	5.7	24.3	8.3	6.6	55.2	100.0
Albania	AL022	7.5	30.6	9.9	9.2	63.6	120.8	6.2	25.3	8.2	7.6	52.6	100.0
Albania	AL031	6.6	29.7	9.3	8.8	66.5	120.9	5.5	24.6	7.7	7.3	55.0	100.0
Albania	AL032	6.1	31.8	9.3	11.0	62.8	121.0	5.0	26.3	7.7	9.1	51.9	100.0
Albania	AL033	5.4	30.5	9.2	9.2	67.9	122.2	4.4	25.0	7.5	7.5	55.6	100.0
Albania	AL034	4.4	29.2	10.2	7.0	69.8	120.6	3.6	24.2	8.5	5.8	57.9	100.0
Albania	AL035	5.2	33.1	9.3	12.8	64.5	124.9	4.2	26.5	7.4	10.2	51.6	100.0
Austria	AT111	7.9	37.4	3.7	4.4	52.7	106.1	7.4	35.2	3.5	4.1	49.7	100.0
Austria	AT112	7.6	36.9	3.6	4.2	51.8	104.1	7.3	35.4	3.5	4.0	49.8	100.0
Austria	AT113	7.4	36.4	3.6	4.4	52.6	104.4	7.1	34.9	3.4	4.2	50.4	100.0
Austria	AT121	10.2	34.8	2.8	4.1	52.9	104.8	9.7	33.2	2.7	3.9	50.5	100.0
Austria	AT122	9.0	36.8	3.4	4.4	55.2	108.8	8.3	33.8	3.1	4.0	50.7	100.0
Austria	AT123	10.6	34.5	3.0	4.0	51.3	103.4	10.3	33.4	2.9	3.9	49.6	100.0
Austria	AT124	7.8	35.7	2.8	4.1	53.3	103.7	7.5	34.4	2.7	4.0	51.4	100.0
Austria	AT125	7.1	36.8	3.4	4.1	52.3	103.7	6.8	35.5	3.3	4.0	50.4	100.0
Austria	AT126	8.9	35.4	3.3	4.0	50.9	102.5	8.7	34.5	3.2	3.9	49.7	100.0
Austria	AT127	9.4	36.0	3.4	4.1	51.7	104.6	9.0	34.4	3.3	3.9	49.4	100.0
Austria	AT130	10.3	35.5	3.3	4.1	51.9	105.1	9.8	33.8	3.1	3.9	49.4	100.0
Austria	AT211	5.7	38.0	3.1	5.5	57.5	109.8	5.2	34.6	2.8	5.0	52.4	100.0
Austria	AT212	4.7	38.2	3.0	5.4	63.3	114.6	4.1	33.3	2.6	4.7	55.2	100.0
Austria	AT213	6.4	37.4	3.3	5.1	57.6	109.8	5.8	34.1	3.0	4.6	52.5	100.0
Austria	AT221	9.0	36.4	3.4	4.6	55.7	109.1	8.2	33.4	3.1	4.2	51.1	100.0
Austria	AT222	6.4	36.3	2.8	4.6	60.4	110.5	5.8	32.9	2.5	4.2	54.7	100.0
Austria	AT223	7.9	36.0	3.2	4.5	57.5	109.1	7.2	33.0	2.9	4.1	52.7	100.0
Austria	AT224	8.0	36.1	3.4	4.5	54.1	106.1	7.5	34.0	3.2	4.2	51.0	100.0
Austria	AT225	7.8	37.6	3.4	4.8	55.6	109.2	7.1	34.4	3.1	4.4	50.9	100.0
Austria	AT226	6.2	36.6	3.1	4.9	60.3	111.1	5.6	32.9	2.8	4.4	54.3	100.0
Austria	AT311	8.1	36.5	2.0	3.8	51.4	101.8	8.0	35.9	2.0	3.7	50.5	100.0
Austria	AT312	10.3	34.1	2.3	3.8	51.3	101.8	10.1	33.5	2.3	3.7	50.4	100.0
Austria	AT313	8.8	35.8	2.5	4.1	53.8	105.0	8.4	34.1	2.4	3.9	51.2	100.0

Austria	AT314	9.1	35.0	2.5	4.1	54.5	105.2	8.7	33.3	2.4	3.9	51.8	100.0
Austria	AT315	8.2	36.1	2.3	4.1	55.3	106.0	7.7	34.1	2.2	3.9	52.2	100.0
Austria	AT321	5.0	37.7	3.0	5.1	65.6	116.4	4.3	32.4	2.6	4.4	56.4	100.0
Austria	AT322	5.2	36.7	2.5	4.6	66.6	115.6	4.5	31.7	2.2	4.0	57.6	100.0
Austria	AT323	7.0	37.3	2.1	4.0	55.8	106.2	6.6	35.1	2.0	3.8	52.5	100.0
Austria	AT331	2.9	38.2	1.9	4.5	63.6	111.1	2.6	34.4	1.7	4.1	57.2	100.0
Austria	AT332	4.1	36.7	2.2	4.6	69.7	117.3	3.5	31.3	1.9	3.9	59.4	100.0
Austria	AT333	3.6	36.4	2.7	5.0	70.9	118.6	3.0	30.7	2.3	4.2	59.8	100.0
Austria	AT334	3.2	34.8	2.2	4.5	72.4	117.1	2.7	29.7	1.9	3.8	61.8	100.0
Austria	AT335	5.0	37.8	2.2	4.4	63.2	112.6	4.4	33.6	2.0	3.9	56.1	100.0
Austria	AT341	3.7	39.7	1.9	4.7	65.1	115.1	3.2	34.5	1.7	4.1	56.6	100.0
Austria	AT342	3.3	42.7	1.6	4.5	55.8	107.9	3.1	39.6	1.5	4.2	51.7	100.0
Belgium	BE10	6.3	28.1	1.1	6.5	56.6	98.6	6.4	28.5	1.1	6.6	57.4	100.0
Belgium	BE21	5.8	28.5	1.1	6.3	55.5	97.2	6.0	29.3	1.1	6.5	57.1	100.0
Belgium	BE22	6.6	30.4	1.1	6.0	54.8	98.9	6.7	30.7	1.1	6.1	55.4	100.0
Belgium	BE23	4.8	25.0	1.0	6.6	55.0	92.4	5.2	27.1	1.1	7.1	59.5	100.0
Belgium	BE24	6.4	28.0	1.1	6.3	55.7	97.5	6.6	28.7	1.1	6.5	57.1	100.0
Belgium	BE25	3.4	25.4	1.1	7.6	57.8	95.3	3.6	26.7	1.2	8.0	60.7	100.0
Belgium	BE31	6.0	27.3	1.1	6.1	53.9	94.4	6.4	28.9	1.2	6.5	57.1	100.0
Belgium	BE32	4.3	26.8	1.1	6.4	54.3	92.9	4.6	28.8	1.2	6.9	58.4	100.0
Belgium	BE33	5.7	30.8	1.2	5.6	54.6	97.9	5.8	31.5	1.2	5.7	55.8	100.0
Belgium	BE34	4.1	31.8	1.2	5.6	55.1	97.8	4.2	32.5	1.2	5.7	56.3	100.0
Belgium	BE35	4.8	29.3	1.1	5.9	54.5	95.6	5.0	30.6	1.2	6.2	57.0	100.0
Bulgaria	BG311	4.7	29.1	9.3	3.9	55.0	102.0	4.6	28.5	9.1	3.8	53.9	100.0
Bulgaria	BG312	6.1	27.3	9.8	4.0	56.3	103.5	5.9	26.4	9.5	3.9	54.4	100.0
Bulgaria	BG313	6.9	25.6	10.0	3.8	55.5	101.8	6.8	25.1	9.8	3.7	54.5	100.0
Bulgaria	BG314	6.9	24.3	10.8	3.8	55.0	100.8	6.8	24.1	10.7	3.8	54.6	100.0
Bulgaria	BG315	7.9	23.6	10.9	4.1	58.1	104.6	7.6	22.6	10.4	3.9	55.5	100.0
Bulgaria	BG321	7.4	22.0	12.0	4.3	56.0	101.7	7.3	21.6	11.8	4.2	55.1	100.0
Bulgaria	BG322	7.8	22.7	11.7	4.4	57.8	104.4	7.5	21.7	11.2	4.2	55.4	100.0
Bulgaria	BG323	5.9	22.6	12.6	4.2	55.5	100.8	5.9	22.4	12.5	4.2	55.1	100.0
Bulgaria	BG324	5.1	21.5	13.5	4.7	55.6	100.4	5.1	21.4	13.4	4.7	55.4	100.0
Bulgaria	BG325	4.0	21.5	14.1	4.7	55.0	99.3	4.0	21.7	14.2	4.7	55.4	100.0
Bulgaria	BG331	5.0	18.4	16.3	6.5	58.4	104.6	4.8	17.6	15.6	6.2	55.8	100.0
Bulgaria	BG332	3.2	19.3	17.1	6.7	59.6	105.9	3.0	18.2	16.1	6.3	56.3	100.0
Bulgaria	BG333	5.6	19.9	14.5	5.4	56.9	102.3	5.5	19.5	14.2	5.3	55.6	100.0
Bulgaria	BG334	6.4	21.1	13.4	4.8	57.1	102.8	6.2	20.5	13.0	4.7	55.5	100.0
Bulgaria	BG341	5.3	18.3	16.5	6.9	60.0	107.0	5.0	17.1	15.4	6.4	56.1	100.0
Bulgaria	BG342	7.5	20.6	13.9	5.3	59.8	107.1	7.0	19.2	13.0	4.9	55.8	100.0
Bulgaria	BG343	6.9	18.9	14.9	5.9	60.0	106.6	6.5	17.7	14.0	5.5	56.3	100.0

Bulgaria	BG344	8.9	20.6	12.5	4.9	59.0	105.9	8.4	19.5	11.8	4.6	55.7	100.0
Bulgaria	BG411	9.1	23.6	10.0	4.1	61.0	107.8	8.4	21.9	9.3	3.8	56.6	100.0
Bulgaria	BG412	8.3	24.3	10.4	4.3	62.1	109.4	7.6	22.2	9.5	3.9	56.8	100.0
Bulgaria	BG413	6.4	23.1	10.4	4.7	67.7	112.3	5.7	20.6	9.3	4.2	60.3	100.0
Bulgaria	BG414	7.1	25.6	9.8	4.3	62.2	109.0	6.5	23.5	9.0	3.9	57.1	100.0
Bulgaria	BG415	6.8	25.1	10.1	4.5	65.3	111.8	6.1	22.5	9.0	4.0	58.4	100.0
Bulgaria	BG421	10.3	21.2	11.2	4.5	59.4	106.6	9.7	19.9	10.5	4.2	55.7	100.0
Bulgaria	BG422	9.1	19.7	13.5	5.5	59.3	107.1	8.5	18.4	12.6	5.1	55.4	100.0
Bulgaria	BG423	9.9	22.0	10.7	4.4	62.8	109.8	9.0	20.0	9.7	4.0	57.2	100.0
Bulgaria	BG424	7.7	21.8	11.5	4.9	65.3	111.2	6.9	19.6	10.3	4.4	58.7	100.0
Bulgaria	BG425	9.0	21.1	12.8	5.6	60.9	109.4	8.2	19.3	11.7	5.1	55.7	100.0
Switzerland	CH011	6.9	31.8	1.6	5.2	59.5	105.0	6.6	30.3	1.5	5.0	56.7	100.0
Switzerland	CH012	4.9	31.3	2.0	4.9	75.9	119.0	4.1	26.3	1.7	4.1	63.8	100.0
Switzerland	CH013	5.4	33.8	1.6	5.6	60.1	106.5	5.1	31.7	1.5	5.3	56.4	100.0
Switzerland	CH021	8.2	31.2	1.7	4.8	61.8	107.7	7.6	29.0	1.6	4.5	57.4	100.0
Switzerland	CH022	8.2	30.2	1.5	4.9	57.5	102.3	8.0	29.5	1.5	4.8	56.2	100.0
Switzerland	CH023	8.6	32.2	1.4	4.7	53.7	100.6	8.5	32.0	1.4	4.7	53.4	100.0
Switzerland	CH024	5.6	33.6	1.5	5.3	58.5	104.5	5.4	32.2	1.4	5.1	56.0	100.0
Switzerland	CH025	4.9	35.3	1.4	5.1	55.5	102.2	4.8	34.5	1.4	5.0	54.3	100.0
Switzerland	CH031	4.9	38.2	1.4	4.8	55.4	104.7	4.7	36.5	1.3	4.6	52.9	100.0
Switzerland	CH032	7.4	34.2	1.3	4.7	53.6	101.2	7.3	33.8	1.3	4.6	53.0	100.0
Switzerland	CH033	10.0	32.3	1.4	4.5	52.4	100.6	9.9	32.1	1.4	4.5	52.1	100.0
Switzerland	CH040	11.1	32.2	1.5	4.5	52.4	101.7	10.9	31.7	1.5	4.4	51.5	100.0
Switzerland	CH051	8.3	37.5	2.0	5.3	66.2	119.3	7.0	31.4	1.7	4.4	55.5	100.0
Switzerland	CH052	6.6	34.7	1.4	4.5	52.8	100.0	6.6	34.7	1.4	4.5	52.8	100.0
Switzerland	CH053	10.7	33.8	1.6	4.5	54.6	105.2	10.2	32.1	1.5	4.3	51.9	100.0
Switzerland	CH054	10.4	34.3	1.6	4.6	55.8	106.7	9.7	32.1	1.5	4.3	52.3	100.0
Switzerland	CH055	10.0	34.6	1.7	4.7	57.7	108.7	9.2	31.8	1.6	4.3	53.1	100.0
Switzerland	CH056	5.3	36.9	2.2	5.2	72.8	122.4	4.3	30.1	1.8	4.2	59.5	100.0
Switzerland	CH057	10.3	33.2	1.5	4.4	52.3	101.7	10.1	32.6	1.5	4.3	51.4	100.0
Switzerland	CH061	10.9	30.0	1.5	4.6	54.7	101.7	10.7	29.5	1.5	4.5	53.8	100.0
Switzerland	CH062	8.0	38.9	2.1	5.5	66.0	120.5	6.6	32.3	1.7	4.6	54.8	100.0
Switzerland	CH063	10.1	35.8	1.8	5.1	60.4	113.2	8.9	31.6	1.6	4.5	53.4	100.0
Switzerland	CH064	10.4	32.2	1.8	4.9	61.3	110.6	9.4	29.1	1.6	4.4	55.4	100.0
Switzerland	CH065	11.4	33.8	1.8	4.9	59.5	111.4	10.2	30.3	1.6	4.4	53.4	100.0
Switzerland	CH066	11.4	32.5	1.6	4.6	54.4	104.5	10.9	31.1	1.5	4.4	52.1	100.0
Switzerland	CH070	5.3	45.4	2.3	6.0	65.0	124.0	4.3	36.6	1.9	4.8	52.4	100.0
Cyprus	CY000	2.6	14.8	24.0	18.4	69.4	129.2	2.0	11.5	18.6	14.2	53.7	100.0
Czechia	CZ010	8.7	33.7	2.3	4.3	52.9	101.9	8.5	33.1	2.3	4.2	51.9	100.0
Czechia	CZ020	7.8	33.7	2.4	4.3	52.4	100.6	7.8	33.5	2.4	4.3	52.1	100.0

Czechia	CZ031	5.1	36.2	2.4	4.1	53.6	101.4	5.0	35.7	2.4	4.0	52.9	100.0
Czechia	CZ032	4.8	35.7	2.1	4.3	53.9	100.8	4.8	35.4	2.1	4.3	53.5	100.0
Czechia	CZ041	4.9	35.2	2.0	4.5	54.6	101.2	4.8	34.8	2.0	4.4	54.0	100.0
Czechia	CZ042	7.0	34.2	2.1	4.5	52.8	100.6	7.0	34.0	2.1	4.5	52.5	100.0
Czechia	CZ051	7.0	35.2	2.5	4.6	52.9	102.2	6.8	34.4	2.4	4.5	51.8	100.0
Czechia	CZ052	7.4	34.5	2.9	4.5	52.7	102.0	7.3	33.8	2.8	4.4	51.7	100.0
Czechia	CZ053	7.8	34.2	3.1	4.3	52.1	101.5	7.7	33.7	3.1	4.2	51.3	100.0
Czechia	CZ063	7.0	35.5	3.0	4.3	53.1	102.9	6.8	34.5	2.9	4.2	51.6	100.0
Czechia	CZ064	7.0	36.1	3.5	4.2	52.5	103.3	6.8	34.9	3.4	4.1	50.8	100.0
Czechia	CZ071	7.1	35.6	3.7	4.4	52.9	103.7	6.8	34.3	3.6	4.2	51.0	100.0
Czechia	CZ072	6.5	36.0	3.9	4.2	52.3	102.9	6.3	35.0	3.8	4.1	50.8	100.0
Czechia	CZ080	6.1	36.7	4.0	4.3	53.3	104.4	5.8	35.2	3.8	4.1	51.1	100.0
Germany	DE11	18.1	24.8	1.5	4.4	52.9	101.7	17.8	24.4	1.5	4.3	52.0	100.0
Germany	DE12	16.4	26.0	1.4	4.6	52.7	101.1	16.2	25.7	1.4	4.5	52.1	100.0
Germany	DE13	11.8	29.9	1.4	4.7	54.6	102.4	11.5	29.2	1.4	4.6	53.3	100.0
Germany	DE14	14.2	28.1	1.5	4.5	54.0	102.3	13.9	27.5	1.5	4.4	52.8	100.0
Germany	DE21	16.5	27.2	1.7	4.1	53.8	103.3	16.0	26.3	1.6	4.0	52.1	100.0
Germany	DE22	16.5	27.0	2.0	4.0	52.3	101.8	16.2	26.5	2.0	3.9	51.4	100.0
Germany	DE23	16.8	24.8	1.8	4.2	53.7	101.3	16.6	24.5	1.8	4.1	53.0	100.0
Germany	DE24	17.0	23.7	1.7	4.5	53.4	100.3	16.9	23.6	1.7	4.5	53.2	100.0
Germany	DE25	18.2	24.1	1.6	4.3	53.1	101.3	18.0	23.8	1.6	4.2	52.4	100.0
Germany	DE26	18.0	23.3	1.5	4.5	52.0	99.3	18.1	23.5	1.5	4.5	52.4	100.0
Germany	DE27	15.0	27.8	1.6	4.2	53.7	102.3	14.7	27.2	1.6	4.1	52.5	100.0
Germany	DE30	15.4	22.6	2.1	6.0	54.0	100.1	15.4	22.6	2.1	6.0	53.9	100.0
Germany	DE40	14.1	22.0	2.1	5.7	52.0	95.9	14.7	22.9	2.2	5.9	54.2	100.0
Germany	DE50	10.8	18.6	1.4	6.9	51.9	89.6	12.1	20.8	1.6	7.7	57.9	100.0
Germany	DE60	11.5	18.3	1.6	6.8	52.9	91.1	12.6	20.1	1.8	7.5	58.1	100.0
Germany	DE71	17.7	23.4	1.4	4.6	51.9	99.0	17.9	23.6	1.4	4.6	52.4	100.0
Germany	DE72	16.9	22.7	1.3	4.8	52.0	97.7	17.3	23.2	1.3	4.9	53.2	100.0
Germany	DE73	16.8	22.4	1.4	4.9	52.2	97.7	17.2	22.9	1.4	5.0	53.4	100.0
Germany	DE80	10.5	20.5	2.2	7.2	55.5	95.9	10.9	21.4	2.3	7.5	57.9	100.0
Germany	DE91	15.9	21.2	1.5	5.5	52.0	96.1	16.5	22.1	1.6	5.7	54.1	100.0
Germany	DE92	14.4	20.8	1.4	5.9	51.9	94.4	15.3	22.0	1.5	6.3	55.0	100.0
Germany	DE93	12.4	19.2	1.6	6.8	52.3	92.3	13.4	20.8	1.7	7.4	56.7	100.0
Germany	DE94	10.3	21.0	1.3	7.1	53.4	93.1	11.1	22.6	1.4	7.6	57.4	100.0
Germany	DEA1	11.6	24.6	1.1	5.5	52.4	95.2	12.2	25.8	1.2	5.8	55.0	100.0
Germany	DEA2	12.4	25.0	1.2	5.2	53.0	96.8	12.8	25.8	1.2	5.4	54.8	100.0
Germany	DEA3	12.6	23.0	1.2	5.8	52.1	94.7	13.3	24.3	1.3	6.1	55.0	100.0
Germany	DEA4	14.6	21.6	1.3	5.5	51.7	94.7	15.4	22.8	1.4	5.8	54.6	100.0
Germany	DEA5	15.1	23.4	1.3	5.3	53.7	98.8	15.3	23.7	1.3	5.4	54.4	100.0

Germany	DEB1	14.7	24.6	1.2	5.0	53.0	98.5	14.9	25.0	1.2	5.1	53.8	100.0
Germany	DEB2	11.4	26.6	1.2	5.2	53.8	98.2	11.6	27.1	1.2	5.3	54.8	100.0
Germany	DEB3	15.6	25.8	1.3	4.8	52.8	100.3	15.6	25.7	1.3	4.8	52.6	100.0
Germany	DEC0	12.2	27.5	1.3	5.0	54.1	100.1	12.2	27.5	1.3	5.0	54.0	100.0
Germany	DED2	14.6	25.8	2.2	4.7	51.7	99.0	14.7	26.1	2.2	4.7	52.2	100.0
Germany	DED4	15.4	25.1	2.0	4.7	53.3	100.5	15.3	25.0	2.0	4.7	53.0	100.0
Germany	DED5	16.7	22.8	1.9	4.9	52.4	98.7	16.9	23.1	1.9	5.0	53.1	100.0
Germany	DEE0	16.1	21.5	1.7	5.5	52.4	97.2	16.6	22.1	1.7	5.7	53.9	100.0
Germany	DEF0	9.3	18.9	1.9	7.9	55.8	93.8	9.9	20.1	2.0	8.4	59.5	100.0
Germany	DEG0	16.8	23.0	1.7	4.9	52.9	99.3	16.9	23.2	1.7	4.9	53.3	100.0
Denmark	DK011	2.6	22.8	2.6	8.7	59.3	96.0	2.7	23.8	2.7	9.1	61.8	100.0
Denmark	DK012	2.8	22.8	2.6	8.6	60.1	96.9	2.9	23.5	2.7	8.9	62.0	100.0
Denmark	DK013	2.8	22.4	2.5	8.9	60.6	97.2	2.9	23.0	2.6	9.2	62.3	100.0
Denmark	DK014	2.5	27.5	3.3	10.2	59.2	102.7	2.4	26.8	3.2	9.9	57.6	100.0
Denmark	DK021	2.8	22.6	2.5	8.3	59.8	96.0	2.9	23.5	2.6	8.6	62.3	100.0
Denmark	DK022	2.4	24.3	2.5	8.6	60.5	98.3	2.4	24.7	2.5	8.7	61.5	100.0
Denmark	DK031	2.2	24.4	2.3	8.7	60.9	98.5	2.2	24.8	2.3	8.8	61.8	100.0
Denmark	DK032	1.7	23.1	1.9	9.1	59.6	95.4	1.8	24.2	2.0	9.5	62.5	100.0
Denmark	DK041	1.9	20.4	1.9	9.0	59.9	93.1	2.0	21.9	2.0	9.7	64.3	100.0
Denmark	DK042	2.5	20.6	2.1	8.5	59.1	92.8	2.7	22.2	2.3	9.2	63.7	100.0
Denmark	DK050	1.9	19.2	2.0	9.0	60.3	92.4	2.1	20.8	2.2	9.7	65.3	100.0
Estonia	EE001	1.8	17.6	4.0	7.5	54.8	85.7	2.1	20.5	4.7	8.8	63.9	100.0
Estonia	EE004	1.3	19.7	4.1	8.5	56.3	89.9	1.4	21.9	4.6	9.5	62.6	100.0
Estonia	EE008	1.9	16.7	5.4	6.3	53.2	83.5	2.3	20.0	6.5	7.5	63.7	100.0
Estonia	EE009	1.9	16.8	4.4	7.0	53.5	83.6	2.3	20.1	5.3	8.4	64.0	100.0
Estonia	EE00A	1.9	16.5	5.8	6.6	53.7	84.5	2.2	19.5	6.9	7.8	63.6	100.0
Greece	EL301	8.6	20.7	14.7	10.9	64.9	119.8	7.2	17.3	12.3	9.1	54.2	100.0
Greece	EL302	9.1	20.7	14.4	10.7	64.7	119.6	7.6	17.3	12.0	8.9	54.1	100.0
Greece	EL303	9.1	20.5	14.6	11.7	63.3	119.2	7.6	17.2	12.2	9.8	53.1	100.0
Greece	EL304	9.4	20.2	14.6	12.6	61.5	118.3	7.9	17.1	12.3	10.7	52.0	100.0
Greece	EL305	7.5	21.4	15.5	12.5	64.7	121.6	6.2	17.6	12.7	10.3	53.2	100.0
Greece	EL306	8.4	22.1	13.9	9.9	65.9	120.2	7.0	18.4	11.6	8.2	54.8	100.0
Greece	EL307	11.5	23.0	14.5	18.5	64.2	131.7	8.7	17.5	11.0	14.0	48.7	100.0
Greece	EL411	3.9	19.3	21.6	13.6	65.8	124.2	3.1	15.5	17.4	11.0	53.0	100.0
Greece	EL412	4.9	17.8	27.8	18.9	64.2	133.6	3.7	13.3	20.8	14.1	48.1	100.0
Greece	EL413	5.0	19.2	22.7	17.1	65.0	129.0	3.9	14.9	17.6	13.3	50.4	100.0
Greece	EL421	5.4	17.1	29.3	24.0	63.6	139.4	3.9	12.3	21.0	17.2	45.6	100.0
Greece	EL422	6.5	21.9	20.8	23.2	62.6	135.0	4.8	16.2	15.4	17.2	46.4	100.0
Greece	EL431	6.5	20.6	18.1	18.4	66.0	129.6	5.0	15.9	14.0	14.2	50.9	100.0
Greece	EL432	6.2	20.7	20.0	21.4	64.0	132.3	4.7	15.6	15.1	16.2	48.4	100.0

Greece	EL433	6.0	21.6	17.3	18.6	65.4	128.9	4.7	16.8	13.4	14.4	50.7	100.0
Greece	EL434	6.1	23.2	16.0	19.5	65.6	130.4	4.7	17.8	12.3	15.0	50.3	100.0
Greece	EL511	3.0	22.6	15.6	6.8	60.8	108.8	2.8	20.8	14.3	6.3	55.9	100.0
Greece	EL512	4.2	24.4	12.6	6.0	61.8	109.0	3.9	22.4	11.6	5.5	56.7	100.0
Greece	EL513	3.6	24.8	14.4	6.7	62.0	111.5	3.2	22.2	12.9	6.0	55.6	100.0
Greece	EL514	4.9	22.8	10.8	5.1	63.5	107.1	4.6	21.3	10.1	4.8	59.3	100.0
Greece	EL515	5.1	23.3	12.9	7.0	62.1	110.4	4.6	21.1	11.7	6.3	56.3	100.0
Greece	EL521	10.0	23.7	10.7	5.5	61.7	111.6	9.0	21.2	9.6	4.9	55.3	100.0
Greece	EL522	8.6	23.2	11.7	5.9	62.5	111.9	7.7	20.7	10.5	5.3	55.9	100.0
Greece	EL523	7.9	24.1	11.3	5.2	62.2	110.7	7.1	21.8	10.2	4.7	56.2	100.0
Greece	EL524	8.7	24.8	11.1	5.3	62.4	112.3	7.7	22.1	9.9	4.7	55.6	100.0
Greece	EL525	9.6	23.6	11.2	6.0	63.1	113.5	8.5	20.8	9.9	5.3	55.6	100.0
Greece	EL526	6.4	23.0	11.0	5.4	62.8	108.6	5.9	21.2	10.1	5.0	57.8	100.0
Greece	EL527	6.3	23.8	13.7	7.3	63.9	115.0	5.5	20.7	11.9	6.3	55.6	100.0
Greece	EL531	8.2	24.6	10.1	5.8	65.6	114.3	7.2	21.5	8.8	5.1	57.4	100.0
Greece	EL532	5.3	26.7	10.6	6.0	66.3	114.9	4.6	23.2	9.2	5.2	57.7	100.0
Greece	EL533	5.4	27.1	11.8	5.7	66.2	116.2	4.6	23.3	10.2	4.9	57.0	100.0
Greece	EL541	7.4	27.0	9.5	11.0	63.6	118.5	6.2	22.8	8.0	9.3	53.7	100.0
Greece	EL542	6.7	29.6	9.1	12.7	63.5	121.6	5.5	24.3	7.5	10.4	52.2	100.0
Greece	EL543	5.8	27.2	9.4	8.3	68.0	118.7	4.9	22.9	7.9	7.0	57.3	100.0
Greece	EL611	8.8	24.0	10.1	7.1	63.4	113.4	7.8	21.2	8.9	6.3	55.9	100.0
Greece	EL612	9.0	22.9	11.2	6.5	63.6	113.2	8.0	20.2	9.9	5.7	56.2	100.0
Greece	EL613	7.7	24.0	13.6	9.0	64.1	118.4	6.5	20.3	11.5	7.6	54.1	100.0
Greece	EL621	5.6	34.4	9.3	29.8	62.1	141.2	4.0	24.4	6.6	21.1	44.0	100.0
Greece	EL622	5.5	36.1	9.4	20.4	62.0	133.4	4.1	27.1	7.0	15.3	46.5	100.0
Greece	EL623	6.2	34.1	9.4	22.5	63.1	135.3	4.6	25.2	6.9	16.6	46.6	100.0
Greece	EL624	5.8	33.3	9.8	19.2	64.2	132.3	4.4	25.2	7.4	14.5	48.5	100.0
Greece	EL631	6.8	27.1	9.8	12.5	65.6	121.8	5.6	22.2	8.0	10.3	53.9	100.0
Greece	EL632	6.0	25.8	10.5	12.1	68.4	122.8	4.9	21.0	8.6	9.9	55.7	100.0
Greece	EL633	6.1	27.8	9.9	16.3	65.9	126.0	4.8	22.1	7.9	12.9	52.3	100.0
Greece	EL641	6.9	22.7	13.2	9.0	65.7	117.5	5.9	19.3	11.2	7.7	55.9	100.0
Greece	EL642	6.2	23.0	15.7	10.7	64.7	120.3	5.2	19.1	13.1	8.9	53.8	100.0
Greece	EL643	7.4	25.3	10.3	9.3	65.8	118.1	6.3	21.4	8.7	7.9	55.7	100.0
Greece	EL644	7.6	23.4	11.8	8.1	64.7	115.6	6.6	20.2	10.2	7.0	56.0	100.0
Greece	EL645	7.1	23.9	11.1	9.0	67.6	118.7	6.0	20.1	9.4	7.6	57.0	100.0
Greece	EL651	7.6	23.1	11.8	10.9	67.9	121.3	6.3	19.0	9.7	9.0	56.0	100.0
Greece	EL652	7.2	23.0	12.3	9.8	67.9	120.2	6.0	19.1	10.2	8.2	56.5	100.0
Greece	EL653	7.6	24.6	11.6	14.9	66.5	125.2	6.1	19.6	9.3	11.9	53.1	100.0
Spain	ES111	9.3	7.4	0.7	12.2	67.4	97.0	9.6	7.6	0.7	12.6	69.5	100.0
Spain	ES112	12.4	8.0	0.9	10.0	68.1	99.4	12.5	8.0	0.9	10.1	68.5	100.0

Spain	ES113	13.5	8.9	1.2	8.2	72.0	103.8	13.0	8.6	1.2	7.9	69.4	100.0
Spain	ES114	12.7	7.8	0.8	11.2	66.6	99.1	12.8	7.9	0.8	11.3	67.2	100.0
Spain	ES120	13.1	9.1	1.0	10.7	68.7	102.6	12.8	8.9	1.0	10.4	67.0	100.0
Spain	ES130	14.5	11.1	1.2	10.1	68.0	104.9	13.8	10.6	1.1	9.6	64.8	100.0
Spain	ES211	16.7	12.6	1.3	8.7	64.5	103.8	16.1	12.1	1.3	8.4	62.1	100.0
Spain	ES212	12.1	15.0	1.2	9.7	63.6	101.6	11.9	14.8	1.2	9.5	62.6	100.0
Spain	ES213	13.4	13.1	1.1	9.5	62.4	99.5	13.5	13.2	1.1	9.5	62.7	100.0
Spain	ES220	14.1	14.9	1.6	9.1	65.7	105.4	13.4	14.1	1.5	8.6	62.3	100.0
Spain	ES230	17.8	11.2	1.7	7.9	69.1	107.7	16.5	10.4	1.6	7.3	64.2	100.0
Spain	ES241	15.0	14.5	2.3	10.0	69.5	111.3	13.5	13.0	2.1	9.0	62.4	100.0
Spain	ES242	18.1	12.4	2.9	10.8	73.0	117.2	15.4	10.6	2.5	9.2	62.3	100.0
Spain	ES243	17.7	13.2	2.1	9.6	68.1	110.7	16.0	11.9	1.9	8.7	61.5	100.0
Spain	ES300	21.4	9.9	2.7	7.5	76.0	117.5	18.2	8.4	2.3	6.4	64.7	100.0
Spain	ES411	16.1	9.5	2.6	7.1	77.6	112.9	14.3	8.4	2.3	6.3	68.7	100.0
Spain	ES412	15.9	10.2	1.7	7.8	71.0	106.6	14.9	9.6	1.6	7.3	66.6	100.0
Spain	ES413	14.8	8.1	1.4	6.9	75.4	106.6	13.9	7.6	1.3	6.5	70.7	100.0
Spain	ES414	15.0	9.3	1.6	7.4	73.5	106.8	14.0	8.7	1.5	6.9	68.8	100.0
Spain	ES415	13.2	10.9	2.2	7.4	75.3	109.0	12.1	10.0	2.0	6.8	69.1	100.0
Spain	ES416	16.4	9.1	2.4	6.9	75.6	110.4	14.9	8.2	2.2	6.3	68.5	100.0
Spain	ES417	16.1	10.3	2.2	7.4	74.2	110.2	14.6	9.3	2.0	6.7	67.3	100.0
Spain	ES418	15.2	9.0	1.9	6.9	74.2	107.2	14.2	8.4	1.8	6.4	69.2	100.0
Spain	ES419	13.4	9.5	1.7	6.8	74.9	106.3	12.6	8.9	1.6	6.4	70.5	100.0
Spain	ES421	17.3	13.4	4.2	13.0	72.4	120.3	14.4	11.1	3.5	10.8	60.2	100.0
Spain	ES422	16.3	10.3	4.0	9.5	76.2	116.3	14.0	8.9	3.4	8.2	65.5	100.0
Spain	ES423	17.5	12.4	3.4	10.8	75.0	119.1	14.7	10.4	2.9	9.1	63.0	100.0
Spain	ES424	18.1	10.2	2.8	8.2	75.7	115.0	15.7	8.9	2.4	7.1	65.8	100.0
Spain	ES425	17.9	10.3	3.1	8.1	75.6	115.0	15.6	9.0	2.7	7.0	65.7	100.0
Spain	ES431	14.0	13.1	3.6	10.1	73.1	113.9	12.3	11.5	3.2	8.9	64.2	100.0
Spain	ES432	14.8	12.3	2.7	8.3	73.4	111.5	13.3	11.0	2.4	7.4	65.8	100.0
Spain	ES511	17.0	19.2	2.4	15.1	63.9	117.6	14.5	16.3	2.0	12.8	54.3	100.0
Spain	ES512	12.9	21.3	2.1	12.9	64.8	114.0	11.3	18.7	1.8	11.3	56.8	100.0
Spain	ES513	15.8	15.9	2.4	10.9	69.0	114.0	13.9	13.9	2.1	9.6	60.5	100.0
Spain	ES514	14.7	18.8	2.8	16.5	64.1	116.9	12.6	16.1	2.4	14.1	54.8	100.0
Spain	ES521	14.4	18.1	4.6	18.9	66.8	122.8	11.7	14.7	3.7	15.4	54.4	100.0
Spain	ES522	18.3	16.2	3.1	16.6	67.1	121.3	15.1	13.4	2.6	13.7	55.3	100.0
Spain	ES523	18.7	16.3	3.5	17.3	66.5	122.3	15.3	13.3	2.9	14.1	54.4	100.0
Spain	ES531	9.9	23.9	5.2	22.8	63.3	125.1	7.9	19.1	4.2	18.2	50.6	100.0
Spain	ES532	9.3	24.9	4.3	17.7	63.7	119.9	7.8	20.8	3.6	14.8	53.1	100.0
Spain	ES533	7.9	29.8	3.8	19.9	61.9	123.3	6.4	24.2	3.1	16.1	50.2	100.0
Spain	ES611	15.8	14.8	6.1	18.5	71.5	126.7	12.5	11.7	4.8	14.6	56.4	100.0

Spain	ES612	14.1	15.0	7.3	19.9	67.9	124.2	11.4	12.1	5.9	16.0	54.7	100.0
Spain	ES613	17.9	11.5	5.0	12.7	73.8	120.9	14.8	9.5	4.1	10.5	61.0	100.0
Spain	ES614	15.9	11.0	6.2	13.9	77.6	124.6	12.8	8.8	5.0	11.2	62.3	100.0
Spain	ES615	14.5	14.7	5.0	14.3	71.6	120.1	12.1	12.2	4.2	11.9	59.6	100.0
Spain	ES616	18.2	10.5	5.0	11.6	75.8	121.1	15.0	8.7	4.1	9.6	62.6	100.0
Spain	ES617	15.2	13.9	7.2	19.7	71.6	127.6	11.9	10.9	5.6	15.4	56.1	100.0
Spain	ES618	18.4	12.7	5.6	14.8	71.3	122.8	15.0	10.3	4.6	12.1	58.1	100.0
Spain	ES620	16.4	15.9	5.0	17.7	68.2	123.2	13.3	12.9	4.1	14.4	55.4	100.0
Finland	FI193	3.8	10.1	3.0	4.8	53.2	74.9	5.1	13.5	4.0	6.4	71.0	100.0
Finland	FI194	3.1	10.3	2.5	5.1	53.2	74.2	4.2	13.9	3.4	6.9	71.7	100.0
Finland	FI195	3.0	10.5	2.4	5.6	54.2	75.7	4.0	13.9	3.2	7.4	71.6	100.0
Finland	FI196	3.1	12.6	2.6	6.2	54.1	78.6	3.9	16.0	3.3	7.9	68.8	100.0
Finland	FI197	3.8	11.7	2.8	5.5	53.5	77.3	4.9	15.1	3.6	7.1	69.2	100.0
Finland	FI1B1	3.9	14.4	3.7	6.8	55.3	84.1	4.6	17.1	4.4	8.1	65.8	100.0
Finland	FI1C1	3.0	15.1	3.1	7.5	56.2	84.9	3.5	17.8	3.7	8.8	66.2	100.0
Finland	FI1C2	4.2	12.7	3.1	6.0	53.7	79.7	5.3	15.9	3.9	7.5	67.4	100.0
Finland	FI1C3	4.7	12.2	3.5	5.6	54.1	80.1	5.9	15.2	4.4	7.0	67.5	100.0
Finland	FI1C4	4.6	12.4	4.2	5.8	52.9	79.9	5.8	15.5	5.3	7.3	66.2	100.0
Finland	FI1C5	4.2	11.2	4.7	5.3	53.7	79.1	5.3	14.2	5.9	6.7	67.9	100.0
Finland	FI1D1	4.1	10.3	4.1	4.9	53.8	77.2	5.3	13.3	5.3	6.3	69.7	100.0
Finland	FI1D2	3.7	8.6	3.4	4.2	53.1	73.0	5.1	11.8	4.7	5.8	72.7	100.0
Finland	FI1D3	3.3	8.3	4.5	4.0	53.1	73.2	4.5	11.3	6.1	5.5	72.5	100.0
Finland	FI1D5	3.2	9.0	2.5	4.6	53.2	72.5	4.4	12.4	3.4	6.3	73.4	100.0
Finland	FI1D7	1.7	5.6	2.0	3.1	53.5	65.9	2.6	8.5	3.0	4.7	81.2	100.0
Finland	FI1D8	2.8	7.0	3.5	3.5	53.4	70.2	4.0	10.0	5.0	5.0	76.1	100.0
Finland	FI1D9	3.0	7.1	2.8	3.7	52.8	69.4	4.3	10.2	4.0	5.3	76.1	100.0
Finland	FI200	2.1	17.4	3.2	9.0	57.3	89.0	2.4	19.6	3.6	10.1	64.4	100.0
France	FR101	16.4	14.5	1.0	6.6	58.2	96.7	17.0	15.0	1.0	6.8	60.2	100.0
France	FR102	16.1	14.7	1.0	6.1	55.8	93.7	17.2	15.7	1.1	6.5	59.6	100.0
France	FR103	14.8	13.8	1.0	6.7	56.9	93.2	15.9	14.8	1.1	7.2	61.1	100.0
France	FR104	15.5	13.8	1.0	6.4	56.5	93.2	16.6	14.8	1.1	6.9	60.6	100.0
France	FR105	16.2	14.6	1.0	6.8	58.4	97.0	16.7	15.1	1.0	7.0	60.2	100.0
France	FR106	16.3	14.7	1.0	6.5	57.9	96.4	16.9	15.2	1.0	6.7	60.1	100.0
France	FR107	16.5	14.6	1.0	6.5	58.0	96.6	17.1	15.1	1.0	6.7	60.0	100.0
France	FR108	15.0	14.5	1.0	6.7	56.8	94.0	16.0	15.4	1.1	7.1	60.4	100.0
France	FRB01	14.5	13.1	1.0	6.0	56.3	90.9	16.0	14.4	1.1	6.6	61.9	100.0
France	FRB02	13.8	13.3	0.9	6.8	57.9	92.7	14.9	14.3	1.0	7.3	62.5	100.0
France	FRB03	14.1	12.4	1.0	6.4	56.8	90.7	15.5	13.7	1.1	7.1	62.6	100.0
France	FRB04	14.1	12.0	0.9	6.8	57.7	91.5	15.4	13.1	1.0	7.4	63.1	100.0
France	FRB05	14.3	12.8	0.9	6.5	57.6	92.1	15.5	13.9	1.0	7.1	62.5	100.0

France	FRB06	14.8	13.4	1.0	6.1	56.2	91.5	16.2	14.6	1.1	6.7	61.4	100.0
France	FRC11	16.2	16.3	1.3	5.5	55.2	94.5	17.1	17.2	1.4	5.8	58.4	100.0
France	FRC12	14.9	14.0	1.1	5.6	54.5	90.1	16.5	15.5	1.2	6.2	60.5	100.0
France	FRC13	17.7	15.1	1.3	5.4	53.0	92.5	19.1	16.3	1.4	5.8	57.3	100.0
France	FRC14	15.1	14.7	1.1	5.7	55.3	91.9	16.4	16.0	1.2	6.2	60.2	100.0
France	FRC21	17.6	20.5	1.5	5.4	56.5	101.5	17.3	20.2	1.5	5.3	55.7	100.0
France	FRC22	18.3	18.7	1.5	5.6	55.0	99.1	18.5	18.9	1.5	5.7	55.5	100.0
France	FRC23	17.6	18.7	1.3	5.2	53.4	96.2	18.3	19.4	1.4	5.4	55.5	100.0
France	FRC24	18.6	21.8	1.4	5.1	54.8	101.7	18.3	21.4	1.4	5.0	53.9	100.0
France	FRD11	8.9	12.8	0.9	8.4	61.1	92.1	9.7	13.9	1.0	9.1	66.3	100.0
France	FRD12	7.6	12.2	0.8	9.2	62.2	92.0	8.3	13.3	0.9	10.0	67.6	100.0
France	FRD13	11.4	12.4	0.8	7.4	58.8	90.8	12.6	13.7	0.9	8.1	64.8	100.0
France	FRD21	12.4	13.7	0.9	7.3	57.8	92.1	13.5	14.9	1.0	7.9	62.8	100.0
France	FRD22	10.1	15.0	0.9	8.1	58.3	92.4	10.9	16.2	1.0	8.8	63.1	100.0
France	FRE11	10.0	19.3	1.1	7.0	55.8	93.2	10.7	20.7	1.2	7.5	59.9	100.0
France	FRE12	8.9	18.0	1.0	8.0	58.3	94.2	9.4	19.1	1.1	8.5	61.9	100.0
France	FRE21	13.9	17.3	1.1	6.3	54.6	93.2	14.9	18.6	1.2	6.8	58.6	100.0
France	FRE22	13.9	15.4	1.0	6.8	55.9	93.0	14.9	16.6	1.1	7.3	60.1	100.0
France	FRE23	11.0	16.6	1.0	7.6	57.1	93.3	11.8	17.8	1.1	8.1	61.2	100.0
France	FRF11	15.9	24.8	1.3	4.7	51.6	98.3	16.2	25.2	1.3	4.8	52.5	100.0
France	FRF12	17.6	23.1	1.3	4.9	53.5	100.4	17.5	23.0	1.3	4.9	53.3	100.0
France	FRF21	13.5	20.0	1.1	5.8	54.3	94.7	14.3	21.1	1.2	6.1	57.3	100.0
France	FRF22	15.6	16.0	1.1	5.7	55.5	93.9	16.6	17.0	1.2	6.1	59.1	100.0
France	FRF23	15.4	17.1	1.1	5.8	55.1	94.5	16.3	18.1	1.2	6.1	58.3	100.0
France	FRF24	15.9	17.6	1.2	5.5	55.2	95.4	16.7	18.4	1.3	5.8	57.9	100.0
France	FRF31	15.5	20.3	1.2	5.2	53.9	96.1	16.1	21.1	1.2	5.4	56.1	100.0
France	FRF32	15.0	19.5	1.2	5.4	54.7	95.8	15.7	20.4	1.3	5.6	57.1	100.0
France	FRF33	14.9	22.9	1.2	5.1	53.5	97.6	15.3	23.5	1.2	5.2	54.8	100.0
France	FRF34	16.3	19.8	1.3	5.2	54.1	96.7	16.9	20.5	1.3	5.4	55.9	100.0
France	FRG01	11.5	10.3	0.8	8.9	62.1	93.6	12.3	11.0	0.9	9.5	66.3	100.0
France	FRG02	13.3	11.0	0.8	7.6	59.9	92.6	14.4	11.9	0.9	8.2	64.7	100.0
France	FRG03	11.7	11.3	0.8	7.7	59.1	90.6	12.9	12.5	0.9	8.5	65.2	100.0
France	FRG04	12.9	11.9	0.8	7.1	58.1	90.8	14.2	13.1	0.9	7.8	64.0	100.0
France	FRG05	11.9	10.9	0.9	9.1	62.4	95.2	12.5	11.4	0.9	9.6	65.5	100.0
France	FRH01	7.5	10.4	0.7	9.8	63.9	92.3	8.1	11.3	0.8	10.6	69.2	100.0
France	FRH02	6.1	9.1	0.7	10.8	66.9	93.6	6.5	9.7	0.7	11.5	71.5	100.0
France	FRH03	10.4	10.9	0.8	8.7	61.0	91.8	11.3	11.9	0.9	9.5	66.4	100.0
France	FRH04	9.0	9.7	0.8	9.5	63.2	92.2	9.8	10.5	0.9	10.3	68.5	100.0
France	FRI11	15.0	12.7	1.1	7.2	58.8	94.8	15.8	13.4	1.2	7.6	62.0	100.0
France	FRI12	13.8	12.3	1.0	8.5	60.6	96.2	14.3	12.8	1.0	8.8	63.0	100.0

France	FRI13	11.8	12.7	1.0	8.0	59.5	93.0	12.7	13.7	1.1	8.6	64.0	100.0
France	FRI14	15.1	12.8	1.1	7.5	58.2	94.7	15.9	13.5	1.2	7.9	61.5	100.0
France	FRI15	11.7	14.7	1.3	7.9	62.3	97.9	12.0	15.0	1.3	8.1	63.6	100.0
France	FRI21	13.7	14.1	1.3	6.6	59.8	95.5	14.3	14.8	1.4	6.9	62.6	100.0
France	FRI22	13.2	13.3	1.2	6.3	57.5	91.5	14.4	14.5	1.3	6.9	62.8	100.0
France	FRI23	13.8	12.7	1.1	6.7	57.2	91.5	15.1	13.9	1.2	7.3	62.5	100.0
France	FRI31	13.9	12.0	1.0	7.4	58.8	93.1	14.9	12.9	1.1	7.9	63.2	100.0
France	FRI32	12.7	11.6	1.0	8.7	61.4	95.4	13.3	12.2	1.0	9.1	64.4	100.0
France	FRI33	13.2	11.4	0.9	7.8	59.8	93.1	14.2	12.2	1.0	8.4	64.2	100.0
France	FRI34	13.8	11.6	0.9	7.0	58.0	91.3	15.1	12.7	1.0	7.7	63.5	100.0
France	FRJ11	16.2	17.4	1.6	10.6	61.5	107.3	15.1	16.2	1.5	9.9	57.3	100.0
France	FRJ12	17.9	19.9	1.9	9.6	62.6	111.9	16.0	17.8	1.7	8.6	55.9	100.0
France	FRJ13	15.4	19.3	1.7	10.9	63.7	111.0	13.9	17.4	1.5	9.8	57.4	100.0
France	FRJ14	13.7	18.0	1.8	7.3	66.2	107.0	12.8	16.8	1.7	6.8	61.9	100.0
France	FRJ15	12.6	19.8	2.0	9.8	67.6	111.8	11.3	17.7	1.8	8.8	60.5	100.0
France	FRJ21	13.3	16.3	1.7	7.6	66.2	105.1	12.7	15.5	1.6	7.2	63.0	100.0
France	FRJ22	13.7	16.8	1.6	7.8	62.3	102.2	13.4	16.4	1.6	7.6	61.0	100.0
France	FRJ23	15.8	14.8	1.4	8.1	60.3	100.4	15.7	14.7	1.4	8.1	60.1	100.0
France	FRJ24	14.4	13.2	1.2	7.4	58.7	94.9	15.2	13.9	1.3	7.8	61.9	100.0
France	FRJ25	14.8	14.2	1.3	7.3	59.9	97.5	15.2	14.6	1.3	7.5	61.4	100.0
France	FRJ26	11.7	16.1	1.7	7.3	67.2	104.0	11.3	15.5	1.6	7.0	64.6	100.0
France	FRJ27	15.3	16.3	1.5	8.7	60.5	102.3	15.0	15.9	1.5	8.5	59.1	100.0
France	FRJ28	16.0	14.1	1.3	7.9	59.2	98.5	16.2	14.3	1.3	8.0	60.1	100.0
France	FRK11	15.1	13.5	1.2	5.7	54.9	90.4	16.7	14.9	1.3	6.3	60.7	100.0
France	FRK12	13.1	15.8	1.5	6.7	62.3	99.4	13.2	15.9	1.5	6.7	62.7	100.0
France	FRK13	14.3	16.5	1.7	6.4	62.2	101.1	14.1	16.3	1.7	6.3	61.5	100.0
France	FRK14	14.3	14.8	1.4	6.0	59.3	95.8	14.9	15.4	1.5	6.3	61.9	100.0
France	FRK21	20.2	17.8	1.5	5.8	54.8	100.1	20.2	17.8	1.5	5.8	54.7	100.0
France	FRK22	19.1	18.3	1.8	7.4	60.9	107.5	17.8	17.0	1.7	6.9	56.7	100.0
France	FRK23	21.4	19.9	2.0	7.6	60.0	110.9	19.3	17.9	1.8	6.9	54.1	100.0
France	FRK24	20.6	18.7	1.8	6.3	60.7	108.1	19.1	17.3	1.7	5.8	56.2	100.0
France	FRK25	17.0	15.4	1.5	5.9	57.3	97.1	17.5	15.9	1.5	6.1	59.0	100.0
France	FRK26	20.0	15.9	1.5	5.9	55.7	99.0	20.2	16.1	1.5	6.0	56.3	100.0
France	FRK27	16.7	20.5	2.0	5.2	69.3	113.7	14.7	18.0	1.8	4.6	60.9	100.0
France	FRK28	16.3	21.0	1.7	5.2	63.8	108.0	15.1	19.4	1.6	4.8	59.1	100.0
France	FRL01	15.0	23.4	2.5	8.2	71.3	120.4	12.5	19.4	2.1	6.8	59.2	100.0
France	FRL02	14.6	21.3	2.3	6.4	75.0	119.6	12.2	17.8	1.9	5.4	62.7	100.0
France	FRL03	13.2	28.5	2.6	11.8	69.3	125.4	10.5	22.7	2.1	9.4	55.3	100.0
France	FRL04	19.9	23.8	2.2	12.2	60.9	119.0	16.7	20.0	1.8	10.3	51.2	100.0
France	FRL05	16.1	27.2	2.6	12.6	63.8	122.3	13.2	22.2	2.1	10.3	52.2	100.0

France	FRL06	21.0	22.6	2.2	9.4	61.7	116.9	18.0	19.3	1.9	8.0	52.8	100.0
France	FRM01	12.9	28.6	3.8	15.3	64.4	125.0	10.3	22.9	3.0	12.2	51.5	100.0
France	FRM02	10.5	30.4	3.7	15.5	65.8	125.9	8.3	24.1	2.9	12.3	52.3	100.0
Croatia	HR	4.6	36.7	6.5	7.1	56.9	111.8	4.1	32.8	5.8	6.4	50.9	100.0
Hungary	HU110	8.5	35.7	5.5	4.3	56.2	110.2	7.7	32.4	5.0	3.9	51.0	100.0
Hungary	HU120	7.6	33.7	5.5	4.1	54.1	105.0	7.2	32.1	5.2	3.9	51.5	100.0
Hungary	HU211	7.0	35.0	5.1	4.3	54.0	105.4	6.6	33.2	4.8	4.1	51.2	100.0
Hungary	HU212	6.0	37.1	4.8	4.2	53.9	106.0	5.7	35.0	4.5	4.0	50.8	100.0
Hungary	HU213	5.5	38.4	4.7	4.5	54.4	107.5	5.1	35.7	4.4	4.2	50.6	100.0
Hungary	HU221	4.2	39.4	4.1	4.3	52.4	104.4	4.0	37.7	3.9	4.1	50.2	100.0
Hungary	HU222	4.0	39.8	3.9	4.4	52.2	104.3	3.8	38.2	3.7	4.2	50.0	100.0
Hungary	HU223	4.5	38.1	4.3	4.6	51.8	103.3	4.4	36.9	4.2	4.5	50.1	100.0
Hungary	HU231	5.5	34.9	5.8	4.5	53.3	104.0	5.3	33.6	5.6	4.3	51.3	100.0
Hungary	HU232	5.2	36.1	5.0	4.6	53.1	104.0	5.0	34.7	4.8	4.4	51.1	100.0
Hungary	HU233	6.4	34.3	5.5	4.3	54.0	104.5	6.1	32.8	5.3	4.1	51.7	100.0
Hungary	HU311	5.2	32.4	6.4	3.9	53.0	100.9	5.2	32.1	6.3	3.9	52.5	100.0
Hungary	HU312	6.7	33.2	5.9	4.0	54.2	104.0	6.4	31.9	5.7	3.8	52.1	100.0
Hungary	HU313	6.3	34.4	5.3	4.0	54.1	104.1	6.1	33.0	5.1	3.8	52.0	100.0
Hungary	HU321	4.9	30.4	6.9	3.8	53.5	99.5	4.9	30.6	6.9	3.8	53.8	100.0
Hungary	HU322	6.4	32.0	6.3	4.0	54.1	102.8	6.2	31.1	6.1	3.9	52.6	100.0
Hungary	HU323	4.1	29.3	7.7	3.7	52.5	97.3	4.2	30.1	7.9	3.8	54.0	100.0
Hungary	HU331	6.8	33.1	5.9	4.1	53.4	103.3	6.6	32.0	5.7	4.0	51.7	100.0
Hungary	HU332	4.9	31.7	6.8	3.8	54.2	101.4	4.8	31.3	6.7	3.7	53.5	100.0
Hungary	HU333	5.7	33.0	6.5	4.0	53.8	103.0	5.5	32.0	6.3	3.9	52.2	100.0
Ireland	IE041	2.1	7.2	0.6	4.1	66.7	80.7	2.6	8.9	0.7	5.1	82.7	100.0
Ireland	IE042	1.9	6.2	0.6	4.0	68.8	81.5	2.3	7.6	0.7	4.9	84.4	100.0
Ireland	IE051	2.7	6.5	0.6	4.2	67.1	81.1	3.3	8.0	0.7	5.2	82.7	100.0
Ireland	IE052	3.5	7.9	0.6	5.2	66.2	83.4	4.2	9.5	0.7	6.2	79.4	100.0
Ireland	IE053	1.8	6.5	0.6	4.8	71.0	84.7	2.1	7.7	0.7	5.7	83.8	100.0
Ireland	IE061	3.8	8.6	0.6	4.9	65.1	83.0	4.6	10.4	0.7	5.9	78.4	100.0
Ireland	IE062	3.7	8.2	0.6	4.7	64.3	81.5	4.5	10.1	0.7	5.8	78.9	100.0
Ireland	IE063	3.4	7.0	0.6	4.1	64.3	79.4	4.3	8.8	0.8	5.2	81.0	100.0
Iceland	IS001	1.2	4.0	0.5	3.2	69.3	78.2	1.5	5.1	0.6	4.1	88.6	100.0
Iceland	IS002	1.1	4.1	0.5	3.0	68.5	77.2	1.4	5.3	0.6	3.9	88.7	100.0
Italy	ITC11	26.8	22.3	2.1	6.1	64.4	121.7	22.0	18.3	1.7	5.0	52.9	100.0
Italy	ITC12	28.4	22.4	2.2	7.0	59.4	119.4	23.8	18.8	1.8	5.9	49.7	100.0
Italy	ITC13	31.2	22.7	2.2	6.6	62.3	125.0	25.0	18.2	1.8	5.3	49.8	100.0
Italy	ITC14	22.9	25.6	2.2	5.9	66.9	123.5	18.5	20.7	1.8	4.8	54.2	100.0
Italy	ITC15	30.1	23.4	2.2	7.3	55.0	118.0	25.5	19.8	1.9	6.2	46.6	100.0
Italy	ITC16	22.5	22.9	2.2	7.7	63.7	119.0	18.9	19.2	1.8	6.5	53.5	100.0

Italy	ITC17	28.0	22.9	2.2	9.9	55.4	118.4	23.6	19.3	1.9	8.4	46.8	100.0
Italy	ITC18	24.5	25.6	2.6	12.3	56.4	121.4	20.2	21.1	2.1	10.1	46.5	100.0
Italy	ITC20	13.0	23.1	2.1	5.0	79.3	122.5	10.6	18.9	1.7	4.1	64.7	100.0
Italy	ITC31	17.2	28.2	2.6	14.7	63.9	126.6	13.6	22.3	2.1	11.6	50.5	100.0
Italy	ITC32	20.4	27.1	2.6	14.4	58.6	123.1	16.6	22.0	2.1	11.7	47.6	100.0
Italy	ITC33	19.0	28.6	3.0	15.8	59.8	126.2	15.1	22.7	2.4	12.5	47.4	100.0
Italy	ITC34	17.8	28.8	3.2	15.9	58.4	124.1	14.3	23.2	2.6	12.8	47.1	100.0
Italy	ITC41	31.5	25.8	2.3	7.0	58.9	125.5	25.1	20.6	1.8	5.6	46.9	100.0
Italy	ITC42	30.3	26.2	2.5	6.8	62.1	127.9	23.7	20.5	2.0	5.3	48.6	100.0
Italy	ITC43	31.6	25.2	2.6	6.6	62.6	128.6	24.6	19.6	2.0	5.1	48.7	100.0
Italy	ITC44	19.3	24.8	2.4	5.4	73.1	125.0	15.4	19.8	1.9	4.3	58.5	100.0
Italy	ITC46	32.3	24.1	2.6	6.6	60.5	126.1	25.6	19.1	2.1	5.2	48.0	100.0
Italy	ITC47	28.3	24.3	2.7	6.5	59.1	120.9	23.4	20.1	2.2	5.4	48.9	100.0
Italy	ITC48	26.5	24.8	2.6	9.7	55.2	118.8	22.3	20.9	2.2	8.2	46.5	100.0
Italy	ITC49	28.3	23.6	2.5	7.9	53.5	115.8	24.4	20.4	2.2	6.8	46.2	100.0
Italy	ITC4A	27.4	23.7	2.6	7.4	53.1	114.2	24.0	20.8	2.3	6.5	46.5	100.0
Italy	ITC4B	26.0	24.6	2.8	7.3	52.4	113.1	23.0	21.8	2.5	6.5	46.3	100.0
Italy	ITC4C	30.8	24.7	2.5	8.3	56.1	122.4	25.2	20.2	2.0	6.8	45.8	100.0
Italy	ITC4D	34.0	25.3	2.6	7.6	58.5	128.0	26.6	19.8	2.0	5.9	45.7	100.0
Italy	ITF11	15.4	24.9	5.4	10.5	66.5	122.7	12.6	20.3	4.4	8.6	54.2	100.0
Italy	ITF12	14.8	26.2	5.3	10.9	61.2	118.4	12.5	22.1	4.5	9.2	51.7	100.0
Italy	ITF13	15.4	25.4	5.5	10.5	62.3	119.1	12.9	21.3	4.6	8.8	52.3	100.0
Italy	ITF14	15.1	25.6	5.8	11.0	62.2	119.7	12.6	21.4	4.8	9.2	52.0	100.0
Italy	ITF21	16.4	24.2	5.6	11.1	63.5	120.8	13.6	20.0	4.6	9.2	52.6	100.0
Italy	ITF22	15.1	25.9	6.0	11.7	61.3	120.0	12.6	21.6	5.0	9.8	51.1	100.0
Italy	ITF31	17.7	24.3	5.5	14.4	60.1	122.0	14.5	19.9	4.5	11.8	49.3	100.0
Italy	ITF32	17.8	25.1	5.8	12.5	60.4	121.6	14.6	20.6	4.8	10.3	49.7	100.0
Italy	ITF33	18.5	24.9	5.5	17.6	59.8	126.3	14.6	19.7	4.4	13.9	47.3	100.0
Italy	ITF34	17.7	25.1	6.1	12.6	61.8	123.3	14.4	20.4	4.9	10.2	50.1	100.0
Italy	ITF35	15.9	24.3	6.1	15.2	62.3	123.8	12.8	19.6	4.9	12.3	50.3	100.0
Italy	ITF43	14.8	27.2	7.8	13.3	62.7	125.8	11.8	21.6	6.2	10.6	49.8	100.0
Italy	ITF44	12.6	29.3	8.6	15.4	61.5	127.4	9.9	23.0	6.8	12.1	48.3	100.0
Italy	ITF45	12.5	29.0	8.7	16.9	61.4	128.5	9.7	22.6	6.8	13.2	47.8	100.0
Italy	ITF46	14.8	27.5	6.6	12.7	60.9	122.5	12.1	22.4	5.4	10.4	49.7	100.0
Italy	ITF47	14.0	27.6	7.5	13.5	62.3	124.9	11.2	22.1	6.0	10.8	49.9	100.0
Italy	ITF48	14.8	26.6	6.9	12.3	61.6	122.2	12.1	21.8	5.6	10.1	50.4	100.0
Italy	ITF51	14.4	24.4	6.5	12.2	64.0	121.5	11.9	20.1	5.3	10.0	52.7	100.0
Italy	ITF52	14.8	24.8	6.7	11.6	63.3	121.2	12.2	20.5	5.5	9.6	52.2	100.0
Italy	ITF61	13.5	23.8	6.9	14.1	64.5	122.8	11.0	19.4	5.6	11.5	52.5	100.0
Italy	ITF62	14.1	23.8	7.3	13.8	63.5	122.5	11.5	19.4	6.0	11.3	51.8	100.0

Italy	ITF63	13.3	23.1	6.8	16.3	62.8	122.3	10.9	18.9	5.6	13.3	51.3	100.0
Italy	ITF64	13.6	23.9	6.6	19.3	62.0	125.4	10.8	19.1	5.3	15.4	49.4	100.0
Italy	ITF65	13.5	23.4	6.6	19.3	62.3	125.1	10.8	18.7	5.3	15.4	49.8	100.0
Italy	ITG11	11.6	25.3	6.6	20.3	61.8	125.6	9.2	20.1	5.3	16.2	49.2	100.0
Italy	ITG12	12.4	23.8	6.6	17.6	64.4	124.8	9.9	19.1	5.3	14.1	51.6	100.0
Italy	ITG13	13.3	23.4	6.6	18.9	63.7	125.9	10.6	18.6	5.2	15.0	50.6	100.0
Italy	ITG14	11.9	23.5	6.7	18.4	63.9	124.4	9.6	18.9	5.4	14.8	51.4	100.0
Italy	ITG15	12.4	22.4	6.6	18.5	64.3	124.2	10.0	18.0	5.3	14.9	51.8	100.0
Italy	ITG16	12.8	22.0	6.7	16.2	67.3	125.0	10.2	17.6	5.4	13.0	53.8	100.0
Italy	ITG17	13.7	21.5	6.8	16.9	66.5	125.4	10.9	17.1	5.4	13.5	53.0	100.0
Italy	ITG18	13.0	22.9	7.0	24.0	61.0	127.9	10.2	17.9	5.5	18.8	47.7	100.0
Italy	ITG19	14.1	22.1	7.0	20.8	62.6	126.6	11.1	17.5	5.5	16.4	49.4	100.0
Italy	ITG2D	10.9	30.7	4.2	15.9	63.1	124.8	8.7	24.6	3.4	12.7	50.6	100.0
Italy	ITG2E	10.9	27.9	4.9	14.5	65.0	123.2	8.8	22.6	4.0	11.8	52.8	100.0
Italy	ITG2F	11.4	27.5	5.7	16.9	62.1	123.6	9.2	22.2	4.6	13.7	50.2	100.0
Italy	ITG2G	11.0	28.6	4.7	14.3	62.6	121.2	9.1	23.6	3.9	11.8	51.7	100.0
Italy	ITG2H	11.0	28.4	5.4	16.5	63.0	124.3	8.8	22.8	4.3	13.3	50.7	100.0
Italy	ITH10	13.9	26.2	2.5	4.9	69.3	116.8	11.9	22.4	2.1	4.2	59.3	100.0
Italy	ITH20	21.7	25.1	2.7	5.8	64.9	120.2	18.1	20.9	2.2	4.8	54.0	100.0
Italy	ITH31	27.4	26.1	3.0	7.0	55.2	118.7	23.1	22.0	2.5	5.9	46.5	100.0
Italy	ITH32	26.2	26.2	3.0	6.7	55.4	117.5	22.3	22.3	2.6	5.7	47.1	100.0
Italy	ITH33	19.0	27.0	2.9	6.0	63.2	118.1	16.1	22.9	2.5	5.1	53.5	100.0
Italy	ITH34	23.2	27.2	3.1	7.3	53.9	114.7	20.2	23.7	2.7	6.4	47.0	100.0
Italy	ITH35	19.6	29.6	3.6	9.6	55.3	117.7	16.7	25.1	3.1	8.2	47.0	100.0
Italy	ITH36	23.4	26.4	3.2	7.7	53.3	114.0	20.5	23.2	2.8	6.8	46.8	100.0
Italy	ITH37	20.4	27.5	3.6	9.1	55.0	115.6	17.6	23.8	3.1	7.9	47.6	100.0
Italy	ITH41	19.5	28.5	3.1	7.1	56.4	114.6	17.0	24.9	2.7	6.2	49.2	100.0
Italy	ITH42	16.4	30.7	3.3	7.3	58.1	115.8	14.2	26.5	2.8	6.3	50.2	100.0
Italy	ITH43	14.9	33.2	3.8	8.9	56.9	117.7	12.7	28.2	3.2	7.6	48.3	100.0
Italy	ITH44	12.6	35.2	4.1	8.7	58.0	118.6	10.6	29.7	3.5	7.3	48.9	100.0
Italy	ITH51	24.4	25.7	2.9	9.4	57.4	119.8	20.4	21.5	2.4	7.8	47.9	100.0
Italy	ITH52	22.6	26.6	3.1	9.9	57.9	120.1	18.8	22.1	2.6	8.2	48.2	100.0
Italy	ITH53	23.5	26.3	3.2	9.3	56.3	118.6	19.8	22.2	2.7	7.8	47.5	100.0
Italy	ITH54	22.6	26.3	3.3	8.9	56.5	117.6	19.2	22.4	2.8	7.6	48.0	100.0
Italy	ITH55	21.1	26.7	3.5	9.6	56.6	117.5	18.0	22.7	3.0	8.2	48.2	100.0
Italy	ITH56	19.6	27.0	3.7	9.5	55.1	114.9	17.1	23.5	3.2	8.3	48.0	100.0
Italy	ITH57	17.8	28.3	4.0	11.4	57.1	118.6	15.0	23.9	3.4	9.6	48.1	100.0
Italy	ITH58	16.5	28.6	4.1	11.0	59.3	119.5	13.8	23.9	3.4	9.2	49.6	100.0
Italy	ITH59	15.2	28.8	4.4	11.1	59.1	118.6	12.8	24.3	3.7	9.4	49.8	100.0
Italy	ITI11	18.8	28.4	3.3	13.6	59.4	123.5	15.2	23.0	2.7	11.0	48.1	100.0

Italy	ITI12	18.4	28.3	3.4	13.5	59.2	122.8	15.0	23.0	2.8	11.0	48.2	100.0
Italy	ITI13	19.1	28.2	3.6	12.2	58.9	122.0	15.7	23.1	3.0	10.0	48.3	100.0
Italy	ITI14	17.9	27.6	3.7	12.2	58.1	119.5	15.0	23.1	3.1	10.2	48.6	100.0
Italy	ITI15	19.2	28.0	3.6	11.9	58.3	121.0	15.9	23.1	3.0	9.8	48.2	100.0
Italy	ITI16	15.6	30.3	3.6	19.6	59.3	128.4	12.1	23.6	2.8	15.3	46.2	100.0
Italy	ITI17	16.0	28.1	3.5	15.4	57.5	120.5	13.3	23.3	2.9	12.8	47.7	100.0
Italy	ITI18	15.0	27.4	4.0	11.3	59.5	117.2	12.8	23.4	3.4	9.6	50.8	100.0
Italy	ITI19	14.1	27.1	3.9	12.8	59.4	117.3	12.0	23.1	3.3	10.9	50.6	100.0
Italy	ITI1A	13.7	27.9	4.1	15.2	59.7	120.6	11.4	23.1	3.4	12.6	49.5	100.0
Italy	ITI21	14.7	25.9	4.4	10.6	59.8	115.4	12.7	22.4	3.8	9.2	51.8	100.0
Italy	ITI22	15.2	24.9	4.4	11.6	60.0	116.1	13.1	21.4	3.8	10.0	51.7	100.0
Italy	ITI31	14.1	28.1	4.4	10.9	58.7	116.2	12.1	24.2	3.8	9.4	50.5	100.0
Italy	ITI32	13.8	28.1	4.7	11.5	58.7	116.8	11.8	24.1	4.0	9.8	50.3	100.0
Italy	ITI33	13.7	26.9	4.8	10.8	59.7	115.9	11.8	23.2	4.1	9.3	51.5	100.0
Italy	ITI34	14.3	26.9	5.2	11.1	61.8	119.3	12.0	22.5	4.4	9.3	51.8	100.0
Italy	ITI35	13.8	27.2	5.1	11.5	59.8	117.4	11.8	23.2	4.3	9.8	50.9	100.0
Italy	ITI41	14.4	25.7	4.3	14.1	59.4	117.9	12.2	21.8	3.6	12.0	50.4	100.0
Italy	ITI42	16.4	24.9	5.0	11.2	63.9	121.4	13.5	20.5	4.1	9.2	52.6	100.0
Italy	ITI43	17.6	24.6	4.7	14.9	60.7	122.5	14.4	20.1	3.8	12.2	49.6	100.0
Italy	ITI44	17.3	24.5	5.0	16.9	60.8	124.5	13.9	19.7	4.0	13.6	48.8	100.0
Italy	ITI45	17.5	23.4	5.1	12.3	63.5	121.8	14.4	19.2	4.2	10.1	52.1	100.0
Liechtenstein	LI000	0.2	44.8	1.8	4.7	60.3	111.8	0.2	40.1	1.6	4.2	53.9	100.0
Lithuania	LT011	3.1	18.8	8.1	5.2	52.6	87.8	3.5	21.4	9.2	5.9	59.9	100.0
Lithuania	LT021	2.1	20.7	8.4	5.2	52.7	89.1	2.4	23.2	9.4	5.8	59.1	100.0
Lithuania	LT022	3.2	19.6	6.8	5.8	52.0	87.4	3.7	22.4	7.8	6.6	59.5	100.0
Lithuania	LT023	2.1	22.6	5.2	8.3	54.0	92.2	2.3	24.5	5.6	9.0	58.6	100.0
Lithuania	LT024	2.4	21.3	7.1	5.9	52.3	89.0	2.7	23.9	8.0	6.6	58.8	100.0
Lithuania	LT025	3.0	18.5	6.2	5.8	51.7	85.2	3.5	21.7	7.3	6.8	60.7	100.0
Lithuania	LT026	2.9	19.8	5.6	6.6	52.5	87.4	3.3	22.7	6.4	7.6	60.1	100.0
Lithuania	LT027	2.5	20.8	6.1	6.7	51.9	88.0	2.8	23.6	6.9	7.6	59.0	100.0
Lithuania	LT028	2.5	21.0	5.0	7.4	52.7	88.6	2.8	23.7	5.6	8.4	59.5	100.0
Lithuania	LT029	3.0	18.2	7.3	5.4	52.2	86.1	3.5	21.1	8.5	6.3	60.6	100.0
Luxembourg	LU000	1.5	35.2	1.2	5.3	54.2	97.4	1.5	36.1	1.2	5.4	55.6	100.0
Latvia	LV003	1.6	21.2	4.4	8.5	54.3	90.0	1.8	23.6	4.9	9.4	60.3	100.0
Latvia	LV005	2.1	16.7	7.2	5.2	51.6	82.8	2.5	20.2	8.7	6.3	62.3	100.0
Latvia	LV006	2.9	19.2	5.1	7.0	54.2	88.4	3.3	21.7	5.8	7.9	61.3	100.0
Latvia	LV007	2.5	18.7	5.0	7.0	53.0	86.2	2.9	21.7	5.8	8.1	61.5	100.0
Latvia	LV008	2.2	16.9	5.6	6.0	52.1	82.8	2.7	20.4	6.8	7.2	62.9	100.0
Latvia	LV009	2.3	18.6	5.7	6.1	51.5	84.2	2.7	22.1	6.8	7.2	61.2	100.0
Montenegro	ME000	2.1	32.4	9.0	6.7	66.1	116.3	1.8	27.9	7.7	5.8	56.8	100.0

Malta	MT	2.1	35.0	6.7	31.4	54.9	130.1	1.6	26.9	5.1	24.1	42.2	100.0
Netherlands	NL11	4.4	25.0	1.3	7.8	56.3	94.8	4.6	26.4	1.4	8.2	59.4	100.0
Netherlands	NL12	4.4	25.2	1.3	8.2	58.6	97.7	4.5	25.8	1.3	8.4	60.0	100.0
Netherlands	NL13	5.5	25.3	1.2	7.1	54.3	93.4	5.9	27.1	1.3	7.6	58.1	100.0
Netherlands	NL21	6.3	26.2	1.2	6.6	53.2	93.5	6.7	28.0	1.3	7.1	56.9	100.0
Netherlands	NL22	6.8	26.5	1.1	6.2	53.2	93.8	7.2	28.3	1.2	6.6	56.7	100.0
Netherlands	NL23	6.0	26.4	1.2	7.8	58.4	99.8	6.0	26.5	1.2	7.8	58.5	100.0
Netherlands	NL31	6.5	24.6	1.1	6.4	54.2	92.8	7.0	26.5	1.2	6.9	58.4	100.0
Netherlands	NL32	4.8	25.9	1.3	8.2	59.5	99.7	4.8	26.0	1.3	8.2	59.7	100.0
Netherlands	NL33	4.7	23.6	1.1	6.8	54.5	90.7	5.2	26.0	1.2	7.5	60.1	100.0
Netherlands	NL34	2.9	24.6	1.0	7.0	55.3	90.8	3.2	27.1	1.1	7.7	60.9	100.0
Netherlands	NL41	5.6	28.3	1.1	6.1	54.1	95.2	5.9	29.7	1.2	6.4	56.8	100.0
Netherlands	NL42	4.5	32.1	1.1	5.7	53.5	96.9	4.6	33.1	1.1	5.9	55.2	100.0
Norway	NO020	3.4	10.2	1.6	5.4	63.8	84.4	4.0	12.1	1.9	6.4	75.6	100.0
Norway	NO060	2.7	8.1	1.5	5.4	63.3	81.0	3.3	10.0	1.9	6.7	78.1	100.0
Norway	NO071	2.1	6.9	1.4	4.9	64.3	79.6	2.6	8.7	1.8	6.2	80.8	100.0
Norway	NO074	1.5	5.0	1.4	3.6	60.3	71.8	2.1	7.0	1.9	5.0	84.0	100.0
Norway	NO081	4.6	11.5	1.6	5.8	57.1	80.6	5.7	14.3	2.0	7.2	70.8	100.0
Norway	NO082	3.7	11.9	1.7	6.0	61.7	85.0	4.4	14.0	2.0	7.1	72.6	100.0
Norway	NO091	3.2	12.6	1.7	6.3	63.8	87.6	3.7	14.4	1.9	7.2	72.8	100.0
Norway	NO092	2.9	14.2	1.7	7.3	64.2	90.3	3.2	15.7	1.9	8.1	71.1	100.0
Norway	NO0A1	3.1	13.9	1.7	8.1	65.7	92.5	3.4	15.0	1.8	8.8	71.0	100.0
Norway	NO0A2	3.4	11.1	1.6	6.8	68.6	91.5	3.7	12.1	1.7	7.4	75.0	100.0
Norway	NO0A3	3.0	9.1	1.4	6.2	67.8	87.5	3.4	10.4	1.6	7.1	77.5	100.0
Poland	PL213	14.5	25.5	4.6	3.9	50.7	99.2	14.6	25.7	4.6	3.9	51.1	100.0
Poland	PL214	14.1	26.0	4.8	4.1	51.4	100.4	14.0	25.9	4.8	4.1	51.2	100.0
Poland	PL217	13.7	24.3	5.4	3.9	50.4	97.7	14.0	24.9	5.5	4.0	51.6	100.0
Poland	PL218	12.1	26.9	5.7	4.0	52.6	101.3	11.9	26.6	5.6	3.9	51.9	100.0
Poland	PL219	10.9	29.0	5.0	4.1	54.8	103.8	10.5	27.9	4.8	3.9	52.8	100.0
Poland	PL21A	13.7	27.3	4.5	4.1	51.9	101.5	13.5	26.9	4.4	4.0	51.1	100.0
Poland	PL224	14.1	25.9	4.2	4.4	51.7	100.3	14.1	25.8	4.2	4.4	51.5	100.0
Poland	PL225	10.8	31.5	4.5	4.2	54.0	105.0	10.3	30.0	4.3	4.0	51.4	100.0
Poland	PL227	11.6	30.0	4.0	4.1	51.7	101.4	11.4	29.6	3.9	4.0	51.0	100.0
Poland	PL228	13.6	27.1	4.1	4.3	51.9	101.0	13.5	26.8	4.1	4.3	51.4	100.0
Poland	PL229	13.2	28.2	4.0	4.2	51.6	101.2	13.0	27.9	4.0	4.2	51.0	100.0
Poland	PL22A	14.4	28.9	4.4	4.3	54.0	106.0	13.6	27.3	4.2	4.1	50.9	100.0
Poland	PL22B	14.4	26.5	4.5	4.3	52.2	101.9	14.1	26.0	4.4	4.2	51.2	100.0
Poland	PL22C	12.9	28.7	4.2	4.1	51.8	101.7	12.7	28.2	4.1	4.0	50.9	100.0
Poland	PL411	10.0	22.6	3.1	6.0	52.5	94.2	10.6	24.0	3.3	6.4	55.7	100.0
Poland	PL414	12.4	24.0	3.6	5.2	52.9	98.1	12.6	24.5	3.7	5.3	53.9	100.0

Poland	PL415	11.3	25.8	3.2	5.5	53.7	99.5	11.4	25.9	3.2	5.5	54.0	100.0
Poland	PL416	11.9	26.1	3.5	4.9	52.7	99.1	12.0	26.3	3.5	4.9	53.2	100.0
Poland	PL417	10.1	26.7	2.9	5.2	52.1	97.0	10.4	27.5	3.0	5.4	53.7	100.0
Poland	PL418	10.8	24.8	3.1	5.4	52.2	96.3	11.2	25.8	3.2	5.6	54.2	100.0
Poland	PL424	7.4	26.2	2.5	7.0	55.5	98.6	7.5	26.6	2.5	7.1	56.3	100.0
Poland	PL426	7.2	22.6	3.0	8.0	55.6	96.4	7.5	23.4	3.1	8.3	57.7	100.0
Poland	PL427	8.3	23.4	2.7	6.5	53.2	94.1	8.8	24.9	2.9	6.9	56.5	100.0
Poland	PL428	7.0	24.8	2.5	6.9	54.0	95.2	7.4	26.1	2.6	7.2	56.7	100.0
Poland	PL431	8.4	26.7	2.5	5.6	51.4	94.6	8.9	28.2	2.6	5.9	54.3	100.0
Poland	PL432	8.2	29.7	2.5	5.0	51.4	96.8	8.5	30.7	2.6	5.2	53.1	100.0
Poland	PL514	10.5	28.7	3.1	4.6	52.0	98.9	10.6	29.0	3.1	4.7	52.6	100.0
Poland	PL515	7.2	33.6	2.6	4.7	52.8	100.9	7.1	33.3	2.6	4.7	52.3	100.0
Poland	PL516	9.4	29.5	2.8	4.8	51.6	98.1	9.6	30.1	2.9	4.9	52.6	100.0
Poland	PL517	8.3	32.8	3.1	4.6	52.6	101.4	8.2	32.3	3.1	4.5	51.9	100.0
Poland	PL518	10.5	28.6	3.1	4.7	52.0	98.9	10.6	28.9	3.1	4.8	52.6	100.0
Poland	PL523	10.6	30.3	3.5	4.5	52.4	101.3	10.5	29.9	3.5	4.4	51.7	100.0
Poland	PL524	12.2	28.1	3.7	4.5	52.2	100.7	12.1	27.9	3.7	4.5	51.8	100.0
Poland	PL613	11.7	21.1	3.7	5.7	52.8	95.0	12.3	22.2	3.9	6.0	55.6	100.0
Poland	PL616	11.9	20.2	4.1	5.7	53.3	95.2	12.5	21.2	4.3	6.0	56.0	100.0
Poland	PL617	11.7	22.6	3.5	5.6	53.2	96.6	12.1	23.4	3.6	5.8	55.1	100.0
Poland	PL618	10.4	20.8	3.5	6.3	53.3	94.3	11.0	22.1	3.7	6.7	56.5	100.0
Poland	PL619	13.0	21.7	3.9	5.2	53.1	96.9	13.4	22.4	4.0	5.4	54.8	100.0
Poland	PL621	10.3	19.3	4.5	6.5	53.3	93.9	11.0	20.6	4.8	6.9	56.8	100.0
Poland	PL622	9.6	18.2	5.3	6.1	52.4	91.6	10.5	19.9	5.8	6.7	57.2	100.0
Poland	PL623	8.7	17.3	6.6	5.7	52.1	90.4	9.6	19.1	7.3	6.3	57.6	100.0
Poland	PL633	8.0	20.5	4.1	8.2	55.6	96.4	8.3	21.3	4.3	8.5	57.7	100.0
Poland	PL634	7.9	20.5	3.9	8.0	55.3	95.6	8.3	21.4	4.1	8.4	57.8	100.0
Poland	PL636	7.5	21.2	3.4	8.0	55.2	95.3	7.9	22.2	3.6	8.4	57.9	100.0
Poland	PL637	9.1	21.0	3.4	6.9	54.3	94.7	9.6	22.2	3.6	7.3	57.3	100.0
Poland	PL638	10.0	19.7	3.9	6.6	53.5	93.7	10.7	21.0	4.2	7.0	57.1	100.0
Poland	PL711	14.3	23.6	4.1	4.8	52.4	99.2	14.4	23.8	4.1	4.8	52.8	100.0
Poland	PL712	14.2	23.4	4.1	4.8	52.4	98.9	14.4	23.7	4.1	4.9	53.0	100.0
Poland	PL713	14.6	23.6	4.3	4.5	51.4	98.4	14.8	24.0	4.4	4.6	52.2	100.0
Poland	PL714	13.3	25.2	3.9	4.8	52.5	99.7	13.3	25.3	3.9	4.8	52.7	100.0
Poland	PL715	14.2	22.2	4.3	4.8	52.6	98.1	14.5	22.6	4.4	4.9	53.6	100.0
Poland	PL721	14.7	22.6	5.3	4.3	51.3	98.2	15.0	23.0	5.4	4.4	52.2	100.0
Poland	PL722	14.7	23.3	5.3	4.2	51.3	98.8	14.9	23.6	5.4	4.3	51.9	100.0
Poland	PL811	10.9	18.0	7.8	4.4	52.1	93.2	11.7	19.3	8.4	4.7	55.9	100.0
Poland	PL812	10.6	19.0	9.0	4.1	52.4	95.1	11.1	20.0	9.5	4.3	55.1	100.0
Poland	PL814	12.4	19.3	7.2	4.3	52.1	95.3	13.0	20.3	7.6	4.5	54.7	100.0

Poland	PL815	13.3	19.6	6.6	4.3	51.6	95.4	13.9	20.5	6.9	4.5	54.1	100.0
Poland	PL821	10.4	24.3	8.0	3.9	52.6	99.2	10.5	24.5	8.1	3.9	53.0	100.0
Poland	PL822	10.8	20.8	9.0	3.8	51.2	95.6	11.3	21.8	9.4	4.0	53.6	100.0
Poland	PL823	12.8	22.4	6.8	3.9	50.1	96.0	13.3	23.3	7.1	4.1	52.2	100.0
Poland	PL824	13.5	21.8	6.6	4.0	50.5	96.4	14.0	22.6	6.8	4.1	52.4	100.0
Poland	PL841	8.8	16.4	7.8	4.8	51.7	89.5	9.8	18.3	8.7	5.4	57.8	100.0
Poland	PL842	10.1	17.1	7.1	4.8	51.9	91.0	11.1	18.8	7.8	5.3	57.0	100.0
Poland	PL843	8.2	16.8	7.4	5.3	52.2	89.9	9.1	18.7	8.2	5.9	58.1	100.0
Poland	PL911	14.8	20.7	5.4	4.9	53.7	99.5	14.9	20.8	5.4	4.9	54.0	100.0
Poland	PL912	13.7	19.3	5.6	4.7	52.0	95.3	14.4	20.3	5.9	4.9	54.6	100.0
Poland	PL913	14.4	20.3	5.0	4.8	52.2	96.7	14.9	21.0	5.2	5.0	54.0	100.0
Poland	PL921	14.6	21.1	5.5	4.4	51.6	97.2	15.0	21.7	5.7	4.5	53.1	100.0
Poland	PL922	12.6	19.5	4.8	5.2	52.8	94.9	13.3	20.5	5.1	5.5	55.6	100.0
Poland	PL923	13.4	20.7	4.3	5.1	52.9	96.4	13.9	21.5	4.5	5.3	54.9	100.0
Poland	PL924	11.6	18.0	5.6	5.1	52.0	92.3	12.6	19.5	6.1	5.5	56.3	100.0
Poland	PL925	12.4	18.5	6.4	4.6	52.0	93.9	13.2	19.7	6.8	4.9	55.4	100.0
Poland	PL926	14.6	21.1	4.9	4.7	52.2	97.5	15.0	21.6	5.0	4.8	53.5	100.0
Portugal	PT111	5.9	16.6	1.0	11.0	68.2	102.7	5.7	16.2	1.0	10.7	66.4	100.0
Portugal	PT112	8.7	15.7	0.9	11.6	67.7	104.6	8.3	15.0	0.9	11.1	64.7	100.0
Portugal	PT119	11.8	15.3	1.0	10.0	68.0	106.1	11.1	14.4	0.9	9.4	64.1	100.0
Portugal	PT11A	13.3	14.3	0.9	12.9	65.8	107.2	12.4	13.3	0.8	12.0	61.4	100.0
Portugal	PT11B	8.0	16.5	1.3	8.2	72.2	106.2	7.5	15.5	1.2	7.7	68.0	100.0
Portugal	PT11C	16.4	14.4	1.1	10.3	66.8	109.0	15.0	13.2	1.0	9.4	61.3	100.0
Portugal	PT11D	12.2	15.5	1.4	8.6	71.2	108.9	11.2	14.2	1.3	7.9	65.4	100.0
Portugal	PT11E	6.6	16.8	1.5	7.1	73.2	105.2	6.3	16.0	1.4	6.7	69.6	100.0
Portugal	PT150	11.2	15.8	3.9	17.6	69.8	118.3	9.5	13.4	3.3	14.9	59.0	100.0
Portugal	PT16B	7.7	12.8	1.0	15.7	67.1	104.3	7.4	12.3	1.0	15.1	64.3	100.0
Portugal	PT16D	13.0	13.9	0.9	14.5	64.8	107.1	12.1	13.0	0.8	13.5	60.5	100.0
Portugal	PT16E	14.3	14.0	1.1	13.4	66.0	108.8	13.1	12.9	1.0	12.3	60.7	100.0
Portugal	PT16F	11.4	13.7	1.1	14.9	65.8	106.9	10.7	12.8	1.0	13.9	61.6	100.0
Portugal	PT16G	16.5	14.2	1.3	9.9	68.6	110.5	14.9	12.9	1.2	9.0	62.1	100.0
Portugal	PT16H	13.6	16.0	2.0	9.3	71.8	112.7	12.1	14.2	1.8	8.3	63.7	100.0
Portugal	PT16I	15.0	14.2	1.5	12.8	67.9	111.4	13.5	12.7	1.3	11.5	61.0	100.0
Portugal	PT16J	12.8	15.6	1.8	8.7	73.1	112.0	11.4	13.9	1.6	7.8	65.3	100.0
Portugal	PT170	12.1	12.6	1.4	14.8	68.0	108.9	11.1	11.6	1.3	13.6	62.4	100.0
Portugal	PT181	13.9	13.6	2.3	15.9	67.7	113.4	12.3	12.0	2.0	14.0	59.7	100.0
Portugal	PT184	11.7	15.6	3.4	13.7	70.2	114.6	10.2	13.6	3.0	12.0	61.3	100.0
Portugal	PT185	13.5	13.1	1.5	13.3	67.2	108.6	12.4	12.1	1.4	12.2	61.9	100.0
Portugal	PT186	13.6	15.1	2.2	10.8	70.1	111.8	12.2	13.5	2.0	9.7	62.7	100.0
Portugal	PT187	12.8	14.8	2.5	12.2	69.9	112.2	11.4	13.2	2.2	10.9	62.3	100.0

Romania	RO111	7.9	25.7	7.7	3.6	55.2	100.1	7.9	25.7	7.7	3.6	55.1	100.0
Romania	RO112	8.0	20.0	10.0	3.4	57.9	99.3	8.1	20.1	10.1	3.4	58.3	100.0
Romania	RO113	8.8	21.5	8.6	3.4	56.1	98.4	8.9	21.8	8.7	3.5	57.0	100.0
Romania	RO114	6.9	22.0	10.1	3.6	58.0	100.6	6.9	21.9	10.0	3.6	57.7	100.0
Romania	RO115	7.3	23.8	8.6	3.5	53.1	96.3	7.6	24.7	8.9	3.6	55.1	100.0
Romania	RO116	8.1	22.7	8.3	3.4	54.2	96.7	8.4	23.5	8.6	3.5	56.0	100.0
Romania	RO121	9.6	21.1	8.7	3.4	57.1	99.9	9.6	21.1	8.7	3.4	57.2	100.0
Romania	RO122	9.6	18.2	10.3	3.4	58.5	100.0	9.6	18.2	10.3	3.4	58.5	100.0
Romania	RO123	8.8	17.6	11.2	3.5	58.5	99.6	8.8	17.7	11.2	3.5	58.7	100.0
Romania	RO124	7.8	18.5	10.8	3.4	59.6	100.1	7.8	18.5	10.8	3.4	59.5	100.0
Romania	RO125	9.5	18.3	9.5	3.2	55.9	96.4	9.9	19.0	9.9	3.3	58.0	100.0
Romania	RO126	10.2	19.1	9.2	3.3	57.2	99.0	10.3	19.3	9.3	3.3	57.8	100.0
Romania	RO211	7.6	17.0	12.4	3.4	55.5	95.9	7.9	17.7	12.9	3.5	57.9	100.0
Romania	RO212	4.9	17.2	13.9	3.3	53.5	92.8	5.3	18.5	15.0	3.6	57.7	100.0
Romania	RO213	6.1	16.4	14.1	3.4	54.5	94.5	6.5	17.4	14.9	3.6	57.7	100.0
Romania	RO214	7.0	17.9	12.2	3.4	56.0	96.5	7.3	18.5	12.6	3.5	58.0	100.0
Romania	RO215	5.9	19.3	12.0	3.5	57.2	97.9	6.0	19.7	12.3	3.6	58.4	100.0
Romania	RO216	6.1	16.0	15.0	3.7	55.7	96.5	6.3	16.6	15.5	3.8	57.7	100.0
Romania	RO221	8.3	15.6	15.5	4.3	56.4	100.1	8.3	15.6	15.5	4.3	56.3	100.0
Romania	RO222	9.6	17.1	13.3	3.8	57.7	101.5	9.5	16.8	13.1	3.7	56.8	100.0
Romania	RO223	6.5	15.5	17.5	6.1	59.6	105.2	6.2	14.7	16.6	5.8	56.7	100.0
Romania	RO224	7.0	15.7	15.0	3.9	56.1	97.7	7.2	16.1	15.4	4.0	57.4	100.0
Romania	RO225	5.1	14.9	18.7	5.6	60.4	104.7	4.9	14.2	17.9	5.3	57.7	100.0
Romania	RO226	8.5	17.1	13.1	3.7	56.9	99.3	8.6	17.2	13.2	3.7	57.3	100.0
Romania	RO311	11.9	18.5	11.2	3.6	60.1	105.3	11.3	17.6	10.6	3.4	57.1	100.0
Romania	RO312	9.1	16.7	14.2	4.4	55.7	100.1	9.1	16.7	14.2	4.4	55.6	100.0
Romania	RO313	12.6	17.3	11.9	3.6	57.4	102.8	12.3	16.8	11.6	3.5	55.8	100.0
Romania	RO314	10.6	18.1	12.3	3.8	54.6	99.4	10.7	18.2	12.4	3.8	54.9	100.0
Romania	RO315	9.1	16.0	14.7	4.4	56.3	100.5	9.1	15.9	14.6	4.4	56.0	100.0
Romania	RO316	11.3	17.4	12.3	3.7	58.6	103.3	10.9	16.8	11.9	3.6	56.7	100.0
Romania	RO317	10.5	19.3	11.6	3.7	55.3	100.4	10.5	19.2	11.6	3.7	55.1	100.0
Romania	RO321	12.9	17.3	12.9	3.8	56.6	103.5	12.5	16.7	12.5	3.7	54.7	100.0
Romania	RO322	11.8	16.6	12.6	3.7	54.8	99.5	11.9	16.7	12.7	3.7	55.1	100.0
Romania	RO411	11.0	21.4	9.9	3.6	54.6	100.5	10.9	21.3	9.9	3.6	54.3	100.0
Romania	RO412	14.2	20.7	9.9	3.6	58.8	107.2	13.2	19.3	9.2	3.4	54.9	100.0
Romania	RO413	11.6	22.8	9.4	3.6	55.7	103.1	11.3	22.1	9.1	3.5	54.0	100.0
Romania	RO414	11.4	19.4	10.8	3.6	55.6	100.8	11.3	19.2	10.7	3.6	55.2	100.0
Romania	RO415	12.4	19.4	10.3	3.6	59.6	105.3	11.8	18.4	9.8	3.4	56.6	100.0
Romania	RO421	8.3	26.3	7.6	3.6	54.9	100.7	8.2	26.1	7.5	3.6	54.5	100.0
Romania	RO422	9.6	25.9	8.9	3.8	57.1	105.3	9.1	24.6	8.5	3.6	54.2	100.0

Romania	RO423	10.8	22.4	8.7	3.5	58.7	104.1	10.4	21.5	8.4	3.4	56.4	100.0
Romania	RO424	8.0	27.9	7.6	3.7	54.3	101.5	7.9	27.5	7.5	3.6	53.5	100.0
Serbia	RS110	11.0	29.1	8.1	4.1	54.0	106.3	10.3	27.4	7.6	3.9	50.8	100.0
Serbia	RS121	6.1	33.4	6.7	4.2	53.1	103.5	5.9	32.3	6.5	4.1	51.3	100.0
Serbia	RS122	8.2	29.5	7.8	3.8	52.8	102.1	8.0	28.9	7.6	3.7	51.7	100.0
Serbia	RS123	8.6	31.5	7.2	4.2	53.3	104.8	8.2	30.1	6.9	4.0	50.9	100.0
Serbia	RS124	6.3	32.8	6.8	4.0	53.4	103.3	6.1	31.8	6.6	3.9	51.7	100.0
Serbia	RS125	6.3	33.6	6.6	4.2	53.4	104.1	6.1	32.3	6.3	4.0	51.3	100.0
Serbia	RS126	7.8	30.8	7.3	3.9	53.1	102.9	7.6	29.9	7.1	3.8	51.6	100.0
Serbia	RS127	10.0	30.2	7.8	4.2	53.4	105.6	9.5	28.6	7.4	4.0	50.6	100.0
Serbia	RS211	5.9	28.7	8.5	5.0	61.7	109.8	5.4	26.1	7.7	4.6	56.2	100.0
Serbia	RS212	8.9	29.4	8.7	4.6	55.9	107.5	8.3	27.3	8.1	4.3	52.0	100.0
Serbia	RS213	7.9	29.5	9.2	4.5	54.8	105.9	7.5	27.9	8.7	4.2	51.7	100.0
Serbia	RS214	8.0	28.5	8.4	4.6	59.0	108.5	7.4	26.3	7.7	4.2	54.4	100.0
Serbia	RS215	9.0	28.8	8.7	4.1	56.0	106.6	8.4	27.0	8.2	3.8	52.5	100.0
Serbia	RS216	8.2	27.7	8.9	4.4	57.6	106.8	7.7	25.9	8.3	4.1	53.9	100.0
Serbia	RS217	6.8	28.0	8.8	4.8	61.3	109.7	6.2	25.5	8.0	4.4	55.9	100.0
Serbia	RS218	10.1	28.5	8.3	4.2	55.6	106.7	9.5	26.7	7.8	3.9	52.1	100.0
Serbia	RS221	4.6	31.0	9.1	3.8	55.5	104.0	4.4	29.8	8.8	3.7	53.4	100.0
Serbia	RS222	7.4	29.5	8.5	3.9	53.9	103.2	7.2	28.6	8.2	3.8	52.2	100.0
Serbia	RS223	5.4	29.5	9.3	4.1	57.2	105.5	5.1	28.0	8.8	3.9	54.2	100.0
Serbia	RS224	6.6	28.2	10.4	4.6	60.0	109.8	6.0	25.7	9.5	4.2	54.6	100.0
Serbia	RS225	7.3	28.2	9.4	4.3	58.0	107.2	6.8	26.3	8.8	4.0	54.1	100.0
Serbia	RS226	4.5	29.1	10.0	4.4	61.7	109.7	4.1	26.5	9.1	4.0	56.2	100.0
Serbia	RS227	10.1	28.3	8.1	4.0	53.5	104.0	9.7	27.2	7.8	3.8	51.4	100.0
Serbia	RS228	5.3	28.8	10.8	4.7	63.1	112.7	4.7	25.6	9.6	4.2	56.0	100.0
Serbia	RS229	7.2	28.0	9.9	4.7	59.8	109.6	6.6	25.5	9.0	4.3	54.6	100.0
Sweden	SE110	4.1	14.3	2.7	7.6	57.1	85.8	4.8	16.7	3.1	8.9	66.6	100.0
Sweden	SE121	3.6	12.4	2.3	6.4	55.2	79.9	4.5	15.5	2.9	8.0	69.1	100.0
Sweden	SE122	3.7	13.6	2.5	6.9	55.6	82.3	4.5	16.5	3.0	8.4	67.6	100.0
Sweden	SE123	3.4	14.5	2.4	7.1	56.2	83.6	4.1	17.3	2.9	8.5	67.2	100.0
Sweden	SE124	3.2	13.0	2.0	6.3	55.8	80.3	4.0	16.2	2.5	7.8	69.5	100.0
Sweden	SE125	3.5	12.3	2.1	6.1	54.6	78.6	4.5	15.6	2.7	7.8	69.5	100.0
Sweden	SE211	3.0	16.5	2.4	7.4	57.1	86.4	3.5	19.1	2.8	8.6	66.1	100.0
Sweden	SE212	2.7	18.5	2.5	7.9	56.4	88.0	3.1	21.0	2.8	9.0	64.1	100.0
Sweden	SE213	3.0	17.7	2.9	8.1	56.2	87.9	3.4	20.1	3.3	9.2	63.9	100.0
Sweden	SE214	3.3	19.5	3.7	9.2	57.9	93.6	3.5	20.8	4.0	9.8	61.9	100.0
Sweden	SE221	2.8	21.0	2.9	8.6	56.8	92.1	3.0	22.8	3.1	9.3	61.7	100.0
Sweden	SE224	2.4	22.3	2.7	8.5	57.7	93.6	2.6	23.8	2.9	9.1	61.6	100.0
Sweden	SE231	2.4	19.2	2.2	8.3	58.0	90.1	2.7	21.3	2.4	9.2	64.4	100.0

Sweden	SE232	3.0	15.9	2.0	7.4	58.1	86.4	3.5	18.4	2.3	8.6	67.2	100.0
Sweden	SE311	2.5	13.6	1.9	6.3	57.2	81.5	3.1	16.7	2.3	7.7	70.2	100.0
Sweden	SE312	2.1	11.7	1.9	5.5	57.4	78.6	2.7	14.9	2.4	7.0	73.0	100.0
Sweden	SE313	2.5	10.8	2.0	5.5	55.9	76.7	3.3	14.1	2.6	7.2	72.9	100.0
Sweden	SE321	2.3	9.3	1.9	5.1	55.5	74.1	3.1	12.6	2.6	6.9	74.9	100.0
Sweden	SE322	1.5	9.4	1.6	4.8	59.8	77.1	1.9	12.2	2.1	6.2	77.6	100.0
Sweden	SE331	1.8	7.8	1.7	4.3	57.4	73.0	2.5	10.7	2.3	5.9	78.6	100.0
Sweden	SE332	1.9	6.5	1.5	3.6	57.2	70.7	2.7	9.2	2.1	5.1	80.9	100.0
Slovenia	SI	5.2	40.8	3.9	6.2	56.2	112.3	4.6	36.3	3.5	5.5	50.0	100.0
Slovakia	SK010	4.4	39.7	3.9	4.2	52.4	104.6	4.2	38.0	3.7	4.0	50.1	100.0
Slovakia	SK021	4.5	38.7	4.1	4.2	52.7	104.2	4.3	37.1	3.9	4.0	50.6	100.0
Slovakia	SK022	4.9	37.4	4.3	4.1	53.5	104.2	4.7	35.9	4.1	3.9	51.3	100.0
Slovakia	SK023	4.8	38.0	4.6	4.1	53.5	105.0	4.6	36.2	4.4	3.9	51.0	100.0
Slovakia	SK031	4.0	36.8	4.8	4.2	56.1	105.9	3.8	34.7	4.5	4.0	53.0	100.0
Slovakia	SK032	4.7	36.1	5.3	4.1	55.7	105.9	4.4	34.1	5.0	3.9	52.6	100.0
Slovakia	SK041	3.5	33.7	6.8	4.0	54.2	102.2	3.4	33.0	6.7	3.9	53.0	100.0
Slovakia	SK042	3.9	32.9	6.9	3.9	53.8	101.4	3.8	32.4	6.8	3.8	53.1	100.0
United Kingdom	UKC1	7.2	9.5	0.9	6.2	62.7	86.5	8.3	11.0	1.0	7.2	72.5	100.0
United Kingdom	UKC2	6.8	9.1	0.8	6.3	63.4	86.4	7.9	10.5	0.9	7.3	73.4	100.0
United Kingdom	UKD1	6.6	9.7	0.9	6.0	64.4	87.6	7.5	11.1	1.0	6.8	73.5	100.0
United Kingdom	UKD3	7.8	9.7	0.9	5.6	60.9	84.9	9.2	11.4	1.1	6.6	71.7	100.0
United Kingdom	UKD4	6.8	9.5	0.9	5.8	61.6	84.6	8.0	11.2	1.1	6.9	72.8	100.0
United Kingdom	UKD6	7.4	8.9	0.8	5.2	58.7	81.0	9.1	11.0	1.0	6.4	72.5	100.0
United Kingdom	UKD7	6.6	9.3	0.8	5.7	61.8	84.2	7.8	11.0	1.0	6.8	73.4	100.0
United Kingdom	UKE1	8.7	10.4	0.9	6.3	58.6	84.9	10.2	12.2	1.1	7.4	69.0	100.0
United Kingdom	UKE2	7.9	9.9	0.9	6.1	61.0	85.8	9.2	11.5	1.0	7.1	71.1	100.0
United Kingdom	UKE3	9.0	9.5	0.8	5.4	59.0	83.7	10.8	11.4	1.0	6.5	70.5	100.0
United Kingdom	UKE4	8.1	9.3	0.8	5.5	59.5	83.2	9.7	11.2	1.0	6.6	71.5	100.0
United Kingdom	UKF1	9.3	9.6	0.8	5.4	58.1	83.2	11.2	11.5	1.0	6.5	69.8	100.0
United Kingdom	UKF2	10.3	10.4	0.8	5.7	58.6	85.8	12.0	12.1	0.9	6.6	68.3	100.0
United Kingdom	UKF3	10.4	11.1	0.9	6.3	58.9	87.6	11.9	12.7	1.0	7.2	67.2	100.0
United Kingdom	UKG1	8.6	9.5	0.8	5.6	59.7	84.2	10.2	11.3	1.0	6.7	70.9	100.0
United Kingdom	UKG2	7.8	9.2	0.8	5.4	59.4	82.6	9.4	11.1	1.0	6.5	71.9	100.0
United Kingdom	UKG3	9.1	9.8	0.9	5.6	59.8	85.2	10.7	11.5	1.1	6.6	70.2	100.0
United Kingdom	UKH1	11.1	13.3	1.0	6.8	59.0	91.2	12.2	14.6	1.1	7.5	64.7	100.0
United Kingdom	UKH2	11.0	11.9	0.9	6.1	58.9	88.8	12.4	13.4	1.0	6.9	66.3	100.0
United Kingdom	UKH3	11.3	13.6	0.9	6.8	59.4	92.0	12.3	14.8	1.0	7.4	64.6	100.0
United Kingdom	UKI3	11.2	12.7	0.9	6.7	60.6	92.1	12.2	13.8	1.0	7.3	65.8	100.0
United Kingdom	UKI4	11.3	12.8	0.9	6.7	60.7	92.4	12.2	13.9	1.0	7.3	65.7	100.0
United Kingdom	UKI5	11.5	13.1	0.9	6.8	60.9	93.2	12.3	14.1	1.0	7.3	65.3	100.0

United Kingdom	UKI6	11.0	13.0	0.9	7.0	60.5	92.4	11.9	14.1	1.0	7.6	65.5	100.0
United Kingdom	UKI7	11.2	12.5	0.9	6.6	60.7	91.9	12.2	13.6	1.0	7.2	66.1	100.0
United Kingdom	UKJ1	10.0	11.0	0.8	6.1	59.1	87.0	11.5	12.6	0.9	7.0	67.9	100.0
United Kingdom	UKJ2	9.6	13.2	0.9	7.5	59.6	90.8	10.6	14.5	1.0	8.3	65.6	100.0
United Kingdom	UKJ3	8.7	11.6	0.8	7.2	59.8	88.1	9.9	13.2	0.9	8.2	67.9	100.0
United Kingdom	UKJ4	9.8	14.2	0.9	7.6	59.0	91.5	10.7	15.5	1.0	8.3	64.5	100.0
United Kingdom	UKK1	8.7	10.2	0.8	6.3	60.2	86.2	10.1	11.8	0.9	7.3	69.8	100.0
United Kingdom	UKK2	7.0	10.4	0.8	7.3	61.8	87.3	8.0	11.9	0.9	8.4	70.8	100.0
United Kingdom	UKK3	4.1	9.2	0.8	8.2	68.2	90.5	4.5	10.2	0.9	9.1	75.4	100.0
United Kingdom	UKK4	5.4	9.7	0.8	7.5	65.1	88.5	6.1	11.0	0.9	8.5	73.6	100.0
United Kingdom	UKL1	5.7	9.3	0.8	6.5	66.2	88.5	6.4	10.5	0.9	7.3	74.8	100.0
United Kingdom	UKL2	6.6	9.3	0.8	5.9	63.5	86.1	7.7	10.8	0.9	6.9	73.8	100.0
United Kingdom	UKM5	5.4	7.3	0.8	6.2	66.2	85.9	6.3	8.5	0.9	7.2	77.1	100.0
United Kingdom	UKM6	4.1	6.5	0.8	5.5	69.9	86.8	4.7	7.5	0.9	6.3	80.5	100.0
United Kingdom	UKM7	5.4	7.2	0.8	5.5	65.7	84.6	6.4	8.5	0.9	6.5	77.7	100.0
United Kingdom	UKM8	5.2	7.1	0.8	5.1	63.9	82.1	6.3	8.6	1.0	6.2	77.8	100.0
United Kingdom	UKM9	6.0	8.4	0.8	5.7	64.4	85.3	7.0	9.8	0.9	6.7	75.5	100.0
United Kingdom	UKN0	4.1	7.1	0.6	4.5	64.8	81.1	5.1	8.8	0.7	5.5	79.9	100.0

Table S5. O_3 attributable deaths according to O_3 precursors emission sources in European regions, 2015-2017

Country	NUTS ID	Number of deaths						Percentage (%)					
		National	34 European countries	Other countries	Ocean and sea	Outside study domain	Overall	National	34 European countries	Other countries	Ocean and sea	Outside study domain	Overall
Albania	AL011	1	6	2	1	13	23	4.3	26.1	8.7	4.3	56.5	100.0
Albania	AL012	3	12	4	4	24	47	6.4	25.5	8.5	8.5	51.1	100.0
Albania	AL013	0	3	2	1	7	13	0.0	23.1	15.4	7.7	53.8	100.0
Albania	AL014	1	6	2	2	13	24	4.2	25.0	8.3	8.3	54.2	100.0
Albania	AL015	2	12	4	3	24	45	4.4	26.7	8.9	6.7	53.3	100.0
Albania	AL021	3	13	4	3	28	51	5.9	25.5	7.8	5.9	54.9	100.0
Albania	AL022	9	32	10	10	67	128	7.0	25.0	7.8	7.8	52.3	100.0
Albania	AL031	2	7	2	2	15	28	7.1	25.0	7.1	7.1	53.6	100.0
Albania	AL032	3	16	5	6	31	61	4.9	26.2	8.2	9.8	50.8	100.0
Albania	AL033	1	5	1	2	11	20	5.0	25.0	5.0	10.0	55.0	100.0
Albania	AL034	2	13	5	3	31	54	3.7	24.1	9.3	5.6	57.4	100.0
Albania	AL035	2	11	3	4	21	41	4.9	26.8	7.3	9.8	51.2	100.0
Austria	AT111	1	4	0	0	5	10	10.0	40.0	0.0	0.0	50.0	100.0
Austria	AT112	3	13	1	1	18	36	8.3	36.1	2.8	2.8	50.0	100.0
Austria	AT113	2	9	1	1	12	25	8.0	36.0	4.0	4.0	48.0	100.0
Austria	AT121	5	17	1	2	25	50	10.0	34.0	2.0	4.0	50.0	100.0
Austria	AT122	5	21	2	2	31	61	8.2	34.4	3.3	3.3	50.8	100.0
Austria	AT123	3	11	1	1	16	32	9.4	34.4	3.1	3.1	50.0	100.0
Austria	AT124	4	19	1	2	28	54	7.4	35.2	1.9	3.7	51.9	100.0
Austria	AT125	2	12	1	1	17	33	6.1	36.4	3.0	3.0	51.5	100.0
Austria	AT126	6	22	2	2	31	63	9.5	34.9	3.2	3.2	49.2	100.0
Austria	AT127	7	24	2	3	35	71	9.9	33.8	2.8	4.2	49.3	100.0
Austria	AT130	34	118	11	13	172	348	9.8	33.9	3.2	3.7	49.4	100.0
Austria	AT211	4	23	2	3	35	67	6.0	34.3	3.0	4.5	52.2	100.0
Austria	AT212	1	11	1	2	17	32	3.1	34.4	3.1	6.3	53.1	100.0
Austria	AT213	2	12	1	2	19	36	5.6	33.3	2.8	5.6	52.8	100.0
Austria	AT221	7	27	3	3	42	82	8.5	32.9	3.7	3.7	51.2	100.0
Austria	AT222	1	6	0	1	10	18	5.6	33.3	0.0	5.6	55.6	100.0
Austria	AT223	3	15	1	2	24	45	6.7	33.3	2.2	4.4	53.3	100.0
Austria	AT224	4	19	2	2	29	56	7.1	33.9	3.6	3.6	51.8	100.0
Austria	AT225	4	16	1	2	23	46	8.7	34.8	2.2	4.3	50.0	100.0
Austria	AT226	2	9	1	1	15	28	7.1	32.1	3.6	3.6	53.6	100.0
Austria	AT311	4	19	1	2	28	54	7.4	35.2	1.9	3.7	51.9	100.0
Austria	AT312	12	37	3	4	57	113	10.6	32.7	2.7	3.5	50.4	100.0
Austria	AT313	4	13	1	1	20	39	10.3	33.3	2.6	2.6	51.3	100.0
Austria	AT314	3	11	1	1	16	32	9.4	34.4	3.1	3.1	50.0	100.0

Austria	AT315	4	16	1	2	25	48	8.3	33.3	2.1	4.2	52.1	100.0
Austria	AT321	0	1	0	0	3	4	0.0	25.0	0.0	0.0	75.0	100.0
Austria	AT322	1	10	1	1	18	31	3.2	32.3	3.2	3.2	58.1	100.0
Austria	AT323	4	23	1	2	33	63	6.3	36.5	1.6	3.2	52.4	100.0
Austria	AT331	0	2	0	0	3	5	0.0	40.0	0.0	0.0	60.0	100.0
Austria	AT332	2	17	1	2	33	55	3.6	30.9	1.8	3.6	60.0	100.0
Austria	AT333	0	3	0	0	6	9	0.0	33.3	0.0	0.0	66.7	100.0
Austria	AT334	1	6	0	1	11	19	5.3	31.6	0.0	5.3	57.9	100.0
Austria	AT335	2	15	1	2	25	45	4.4	33.3	2.2	4.4	55.6	100.0
Austria	AT341	1	6	0	1	9	17	5.9	35.3	0.0	5.9	52.9	100.0
Austria	AT342	1	19	1	2	25	48	2.1	39.6	2.1	4.2	52.1	100.0
Belgium	BE10	12	54	2	13	109	190	6.3	28.4	1.1	6.8	57.4	100.0
Belgium	BE21	21	98	4	22	197	342	6.1	28.7	1.2	6.4	57.6	100.0
Belgium	BE22	10	47	2	9	84	152	6.6	30.9	1.3	5.9	55.3	100.0
Belgium	BE23	15	75	3	19	163	275	5.5	27.3	1.1	6.9	59.3	100.0
Belgium	BE24	14	60	2	14	120	210	6.7	28.6	1.0	6.7	57.1	100.0
Belgium	BE25	9	67	3	20	149	248	3.6	27.0	1.2	8.1	60.1	100.0
Belgium	BE31	4	20	1	5	40	70	5.7	28.6	1.4	7.1	57.1	100.0
Belgium	BE32	14	84	3	20	169	290	4.8	29.0	1.0	6.9	58.3	100.0
Belgium	BE33	15	77	3	14	134	243	6.2	31.7	1.2	5.8	55.1	100.0
Belgium	BE34	2	18	1	3	31	55	3.6	32.7	1.8	5.5	56.4	100.0
Belgium	BE35	5	31	1	6	58	101	5.0	30.7	1.0	5.9	57.4	100.0
Bulgaria	BG311	2	12	4	2	23	43	4.7	27.9	9.3	4.7	53.5	100.0
Bulgaria	BG312	4	16	6	2	33	61	6.6	26.2	9.8	3.3	54.1	100.0
Bulgaria	BG313	5	17	7	3	38	70	7.1	24.3	10.0	4.3	54.3	100.0
Bulgaria	BG314	7	23	10	4	53	97	7.2	23.7	10.3	4.1	54.6	100.0
Bulgaria	BG315	4	13	6	2	31	56	7.1	23.2	10.7	3.6	55.4	100.0
Bulgaria	BG321	6	19	10	4	47	86	7.0	22.1	11.6	4.7	54.7	100.0
Bulgaria	BG322	4	10	5	2	27	48	8.3	20.8	10.4	4.2	56.3	100.0
Bulgaria	BG323	5	18	10	3	43	79	6.3	22.8	12.7	3.8	54.4	100.0
Bulgaria	BG324	2	8	5	2	22	39	5.1	20.5	12.8	5.1	56.4	100.0
Bulgaria	BG325	2	8	6	2	21	39	5.1	20.5	15.4	5.1	53.8	100.0
Bulgaria	BG331	6	23	22	9	75	135	4.4	17.0	16.3	6.7	55.6	100.0
Bulgaria	BG332	2	11	10	4	35	62	3.2	17.7	16.1	6.5	56.5	100.0
Bulgaria	BG333	3	11	8	3	32	57	5.3	19.3	14.0	5.3	56.1	100.0
Bulgaria	BG334	2	8	5	2	22	39	5.1	20.5	12.8	5.1	56.4	100.0
Bulgaria	BG341	6	21	19	8	69	123	4.9	17.1	15.4	6.5	56.1	100.0
Bulgaria	BG342	5	12	9	3	36	65	7.7	18.5	13.8	4.6	55.4	100.0
Bulgaria	BG343	3	8	7	3	27	48	6.3	16.7	14.6	6.3	56.3	100.0
Bulgaria	BG344	10	23	14	5	64	116	8.6	19.8	12.1	4.3	55.2	100.0

Bulgaria	BG411	30	76	32	13	197	348	8.6	21.8	9.2	3.7	56.6	100.0
Bulgaria	BG412	7	21	9	4	53	94	7.4	22.3	9.6	4.3	56.4	100.0
Bulgaria	BG413	5	19	9	4	56	93	5.4	20.4	9.7	4.3	60.2	100.0
Bulgaria	BG414	4	12	5	2	30	53	7.5	22.6	9.4	3.8	56.6	100.0
Bulgaria	BG415	4	13	5	2	34	58	6.9	22.4	8.6	3.4	58.6	100.0
Bulgaria	BG421	24	42	23	9	119	217	11.1	19.4	10.6	4.1	54.8	100.0
Bulgaria	BG422	8	16	10	4	48	86	9.3	18.6	11.6	4.7	55.8	100.0
Bulgaria	BG423	9	18	9	4	51	91	9.9	19.8	9.9	4.4	56.0	100.0
Bulgaria	BG424	3	8	4	2	23	40	7.5	20.0	10.0	5.0	57.5	100.0
Bulgaria	BG425	4	8	5	2	24	43	9.3	18.6	11.6	4.7	55.8	100.0
Switzerland	CH011	9	37	2	6	68	122	7.4	30.3	1.6	4.9	55.7	100.0
Switzerland	CH012	3	17	1	3	41	65	4.6	26.2	1.5	4.6	63.1	100.0
Switzerland	CH013	4	24	1	4	42	75	5.3	32.0	1.3	5.3	56.0	100.0
Switzerland	CH021	18	60	3	9	112	202	8.9	29.7	1.5	4.5	55.4	100.0
Switzerland	CH022	4	13	1	2	24	44	9.1	29.5	2.3	4.5	54.5	100.0
Switzerland	CH023	4	16	1	2	27	50	8.0	32.0	2.0	4.0	54.0	100.0
Switzerland	CH024	2	11	1	2	20	36	5.6	30.6	2.8	5.6	55.6	100.0
Switzerland	CH025	1	5	0	1	8	15	6.7	33.3	0.0	6.7	53.3	100.0
Switzerland	CH031	2	17	1	2	25	47	4.3	36.2	2.1	4.3	53.2	100.0
Switzerland	CH032	3	18	1	2	28	52	5.8	34.6	1.9	3.8	53.8	100.0
Switzerland	CH033	10	32	1	5	53	101	9.9	31.7	1.0	5.0	52.5	100.0
Switzerland	CH040	26	72	3	10	119	230	11.3	31.3	1.3	4.3	51.7	100.0
Switzerland	CH051	1	3	0	0	5	9	11.1	33.3	0.0	0.0	55.6	100.0
Switzerland	CH052	1	5	0	1	7	14	7.1	35.7	0.0	7.1	50.0	100.0
Switzerland	CH053	1	3	0	0	6	10	10.0	30.0	0.0	0.0	60.0	100.0
Switzerland	CH054	0	1	0	0	1	2	0.0	50.0	0.0	0.0	50.0	100.0
Switzerland	CH055	9	27	1	4	45	86	10.5	31.4	1.2	4.7	52.3	100.0
Switzerland	CH056	2	13	1	2	24	42	4.8	31.0	2.4	4.8	57.1	100.0
Switzerland	CH057	4	13	1	2	21	41	9.8	31.7	2.4	4.9	51.2	100.0
Switzerland	CH061	7	19	1	3	34	64	10.9	29.7	1.6	4.7	53.1	100.0
Switzerland	CH062	1	3	0	0	5	9	11.1	33.3	0.0	0.0	55.6	100.0
Switzerland	CH063	2	8	0	1	13	24	8.3	33.3	0.0	4.2	54.2	100.0
Switzerland	CH064	1	2	0	0	3	6	16.7	33.3	0.0	0.0	50.0	100.0
Switzerland	CH065	1	2	0	0	4	7	14.3	28.6	0.0	0.0	57.1	100.0
Switzerland	CH066	2	5	0	1	9	17	11.8	29.4	0.0	5.9	52.9	100.0
Switzerland	CH070	3	33	2	4	40	82	3.7	40.2	2.4	4.9	48.8	100.0
Cyprus	CY000	3	17	28	21	81	150	2.0	11.3	18.7	14.0	54.0	100.0
Czechia	CZ010	23	89	6	11	139	268	8.6	33.2	2.2	4.1	51.9	100.0
Czechia	CZ020	23	93	7	12	144	279	8.2	33.3	2.5	4.3	51.6	100.0
Czechia	CZ031	7	51	4	6	76	144	4.9	35.4	2.8	4.2	52.8	100.0

Czechia	CZ032	7	46	3	5	70	131	5.3	35.1	2.3	3.8	53.4	100.0
Czechia	CZ041	3	24	1	3	38	69	4.3	34.8	1.4	4.3	55.1	100.0
Czechia	CZ042	14	66	4	9	101	194	7.2	34.0	2.1	4.6	52.1	100.0
Czechia	CZ051	7	34	2	4	51	98	7.1	34.7	2.0	4.1	52.0	100.0
Czechia	CZ052	9	42	4	5	63	123	7.3	34.1	3.3	4.1	51.2	100.0
Czechia	CZ053	9	38	4	5	57	113	8.0	33.6	3.5	4.4	50.4	100.0
Czechia	CZ063	8	38	3	5	57	111	7.2	34.2	2.7	4.5	51.4	100.0
Czechia	CZ064	19	91	9	10	133	262	7.3	34.7	3.4	3.8	50.8	100.0
Czechia	CZ071	11	51	5	6	76	149	7.4	34.2	3.4	4.0	51.0	100.0
Czechia	CZ072	9	47	5	5	69	135	6.7	34.8	3.7	3.7	51.1	100.0
Czechia	CZ080	17	104	12	12	150	295	5.8	35.3	4.1	4.1	50.8	100.0
Germany	DE11	149	206	12	37	437	841	17.7	24.5	1.4	4.4	52.0	100.0
Germany	DE12	99	152	8	27	307	593	16.7	25.6	1.3	4.6	51.8	100.0
Germany	DE13	54	138	6	22	248	468	11.5	29.5	1.3	4.7	53.0	100.0
Germany	DE14	52	102	6	16	196	372	14.0	27.4	1.6	4.3	52.7	100.0
Germany	DE21	151	232	15	35	461	894	16.9	26.0	1.7	3.9	51.6	100.0
Germany	DE22	46	74	5	11	144	280	16.4	26.4	1.8	3.9	51.4	100.0
Germany	DE23	42	61	5	10	132	250	16.8	24.4	2.0	4.0	52.8	100.0
Germany	DE24	46	64	5	12	145	272	16.9	23.5	1.8	4.4	53.3	100.0
Germany	DE25	73	95	7	17	210	402	18.2	23.6	1.7	4.2	52.2	100.0
Germany	DE26	54	69	4	13	154	294	18.4	23.5	1.4	4.4	52.4	100.0
Germany	DE27	61	105	6	16	205	393	15.5	26.7	1.5	4.1	52.2	100.0
Germany	DE30	110	162	15	43	386	716	15.4	22.6	2.1	6.0	53.9	100.0
Germany	DE40	95	144	14	37	339	629	15.1	22.9	2.2	5.9	53.9	100.0
Germany	DE50	18	31	2	11	83	145	12.4	21.4	1.4	7.6	57.2	100.0
Germany	DE60	42	67	6	25	193	333	12.6	20.1	1.8	7.5	58.0	100.0
Germany	DE71	146	191	11	38	426	812	18.0	23.5	1.4	4.7	52.5	100.0
Germany	DE72	41	55	3	12	126	237	17.3	23.2	1.3	5.1	53.2	100.0
Germany	DE73	52	69	4	15	161	301	17.3	22.9	1.3	5.0	53.5	100.0
Germany	DE80	44	87	9	31	239	410	10.7	21.2	2.2	7.6	58.3	100.0
Germany	DE91	65	86	6	22	211	390	16.7	22.1	1.5	5.6	54.1	100.0
Germany	DE92	79	111	8	32	280	510	15.5	21.8	1.6	6.3	54.9	100.0
Germany	DE93	50	78	6	28	215	377	13.3	20.7	1.6	7.4	57.0	100.0
Germany	DE94	59	117	8	39	296	519	11.4	22.5	1.5	7.5	57.0	100.0
Germany	DEA1	158	313	15	69	677	1,232	12.8	25.4	1.2	5.6	55.0	100.0
Germany	DEA2	123	236	11	50	508	928	13.3	25.4	1.2	5.4	54.7	100.0
Germany	DEA3	77	139	7	34	314	571	13.5	24.3	1.2	6.0	55.0	100.0
Germany	DEA4	67	100	6	26	240	439	15.3	22.8	1.4	5.9	54.7	100.0
Germany	DEA5	135	218	11	51	497	912	14.8	23.9	1.2	5.6	54.5	100.0
Germany	DEB1	56	91	5	19	199	370	15.1	24.6	1.4	5.1	53.8	100.0

Germany	DEB2	14	33	2	6	67	122	11.5	27.0	1.6	4.9	54.9	100.0
Germany	DEB3	75	118	6	22	245	466	16.1	25.3	1.3	4.7	52.6	100.0
Germany	DEC0	34	77	4	14	151	280	12.1	27.5	1.4	5.0	53.9	100.0
Germany	DED2	61	108	9	20	215	413	14.8	26.2	2.2	4.8	52.1	100.0
Germany	DED4	69	112	9	21	238	449	15.4	24.9	2.0	4.7	53.0	100.0
Germany	DED5	44	59	5	13	136	257	17.1	23.0	1.9	5.1	52.9	100.0
Germany	DEE0	110	145	12	36	353	656	16.8	22.1	1.8	5.5	53.8	100.0
Germany	DEF0	71	132	13	53	387	656	10.8	20.1	2.0	8.1	59.0	100.0
Germany	DEG0	100	136	10	29	314	589	17.0	23.1	1.7	4.9	53.3	100.0
Denmark	DK011	3	26	3	10	67	109	2.8	23.9	2.8	9.2	61.5	100.0
Denmark	DK012	3	24	3	9	64	103	2.9	23.3	2.9	8.7	62.1	100.0
Denmark	DK013	3	20	2	8	55	88	3.4	22.7	2.3	9.1	62.5	100.0
Denmark	DK014	0	3	0	1	7	11	0.0	27.3	0.0	9.1	63.6	100.0
Denmark	DK021	1	10	1	4	26	42	2.4	23.8	2.4	9.5	61.9	100.0
Denmark	DK022	4	34	4	12	85	139	2.9	24.5	2.9	8.6	61.2	100.0
Denmark	DK031	2	25	2	9	64	102	2.0	24.5	2.0	8.8	62.7	100.0
Denmark	DK032	3	34	3	13	88	141	2.1	24.1	2.1	9.2	62.4	100.0
Denmark	DK041	2	17	2	7	50	78	2.6	21.8	2.6	9.0	64.1	100.0
Denmark	DK042	4	31	3	13	89	140	2.9	22.1	2.1	9.3	63.6	100.0
Denmark	DK050	2	23	2	11	74	112	1.8	20.5	1.8	9.8	66.1	100.0
Estonia	EE001	2	22	5	9	68	106	1.9	20.8	4.7	8.5	64.2	100.0
Estonia	EE004	1	8	2	3	22	36	2.8	22.2	5.6	8.3	61.1	100.0
Estonia	EE008	2	13	4	5	43	67	3.0	19.4	6.0	7.5	64.2	100.0
Estonia	EE009	1	6	2	2	19	30	3.3	20.0	6.7	6.7	63.3	100.0
Estonia	EE00A	1	8	3	3	25	40	2.5	20.0	7.5	7.5	62.5	100.0
Greece	EL301	11	25	18	13	79	146	7.5	17.1	12.3	8.9	54.1	100.0
Greece	EL302	9	21	15	11	65	121	7.4	17.4	12.4	9.1	53.7	100.0
Greece	EL303	25	55	40	31	170	321	7.8	17.1	12.5	9.7	53.0	100.0
Greece	EL304	11	25	18	15	75	144	7.6	17.4	12.5	10.4	52.1	100.0
Greece	EL305	8	19	13	10	57	107	7.5	17.8	12.1	9.3	53.3	100.0
Greece	EL306	3	6	4	3	20	36	8.3	16.7	11.1	8.3	55.6	100.0
Greece	EL307	14	26	19	15	83	157	8.9	16.6	12.1	9.6	52.9	100.0
Greece	EL411	1	5	6	4	18	34	2.9	14.7	17.6	11.8	52.9	100.0
Greece	EL412	1	2	3	2	8	16	6.3	12.5	18.8	12.5	50.0	100.0
Greece	EL413	1	2	3	2	8	16	6.3	12.5	18.8	12.5	50.0	100.0
Greece	EL421	2	5	11	9	22	49	4.1	10.2	22.4	18.4	44.9	100.0
Greece	EL422	2	5	5	6	15	33	6.1	15.2	15.2	18.2	45.5	100.0
Greece	EL431	4	12	11	11	38	76	5.3	15.8	14.5	14.5	50.0	100.0
Greece	EL432	1	4	4	4	12	25	4.0	16.0	16.0	16.0	48.0	100.0
Greece	EL433	1	3	3	3	10	20	5.0	15.0	15.0	15.0	50.0	100.0

Greece	EL434	2	8	6	6	22	44	4.5	18.2	13.6	13.6	50.0	100.0
Greece	EL511	1	9	7	3	25	45	2.2	20.0	15.6	6.7	55.6	100.0
Greece	EL512	1	5	3	1	14	24	4.2	20.8	12.5	4.2	58.3	100.0
Greece	EL513	1	7	4	2	17	31	3.2	22.6	12.9	6.5	54.8	100.0
Greece	EL514	2	6	3	2	18	31	6.5	19.4	9.7	6.5	58.1	100.0
Greece	EL515	2	9	5	3	24	43	4.7	20.9	11.6	7.0	55.8	100.0
Greece	EL521	4	9	4	2	23	42	9.5	21.4	9.5	4.8	54.8	100.0
Greece	EL522	24	53	27	14	144	262	9.2	20.2	10.3	5.3	55.0	100.0
Greece	EL523	2	6	3	1	16	28	7.1	21.4	10.7	3.6	57.1	100.0
Greece	EL524	4	9	4	2	23	42	9.5	21.4	9.5	4.8	54.8	100.0
Greece	EL525	3	8	4	2	20	37	8.1	21.6	10.8	5.4	54.1	100.0
Greece	EL526	4	13	6	3	37	63	6.3	20.6	9.5	4.8	58.7	100.0
Greece	EL527	2	6	3	2	16	29	6.9	20.7	10.3	6.9	55.2	100.0
Greece	EL531	4	11	5	3	29	52	7.7	21.2	9.6	5.8	55.8	100.0
Greece	EL532	1	3	1	1	8	14	7.1	21.4	7.1	7.1	57.1	100.0
Greece	EL533	1	3	2	1	9	16	6.3	18.8	12.5	6.3	56.3	100.0
Greece	EL541	3	9	3	4	22	41	7.3	22.0	7.3	9.8	53.7	100.0
Greece	EL542	1	3	1	2	7	14	7.1	21.4	7.1	14.3	50.0	100.0
Greece	EL543	2	10	4	3	26	45	4.4	22.2	8.9	6.7	57.8	100.0
Greece	EL611	7	16	7	5	43	78	9.0	20.5	9.0	6.4	55.1	100.0
Greece	EL612	6	14	7	4	40	71	8.5	19.7	9.9	5.6	56.3	100.0
Greece	EL613	4	11	6	4	31	56	7.1	19.6	10.7	7.1	55.4	100.0
Greece	EL621	1	3	1	3	6	14	7.1	21.4	7.1	21.4	42.9	100.0
Greece	EL622	2	10	3	5	18	38	5.3	26.3	7.9	13.2	47.4	100.0
Greece	EL623	1	3	1	2	6	13	7.7	23.1	7.7	15.4	46.2	100.0
Greece	EL624	0	2	1	1	5	9	0.0	22.2	11.1	11.1	55.6	100.0
Greece	EL631	4	14	5	7	35	65	6.2	21.5	7.7	10.8	53.8	100.0
Greece	EL632	4	18	7	9	46	84	4.8	21.4	8.3	10.7	54.8	100.0
Greece	EL633	2	11	4	7	26	50	4.0	22.0	8.0	14.0	52.0	100.0
Greece	EL641	2	6	4	2	18	32	6.3	18.8	12.5	6.3	56.3	100.0
Greece	EL642	3	12	8	5	33	61	4.9	19.7	13.1	8.2	54.1	100.0
Greece	EL643	0	1	0	0	3	4	0.0	25.0	0.0	0.0	75.0	100.0
Greece	EL644	3	10	5	3	26	47	6.4	21.3	10.6	6.4	55.3	100.0
Greece	EL645	1	2	1	1	6	11	9.1	18.2	9.1	9.1	54.5	100.0
Greece	EL651	4	11	6	5	32	58	6.9	19.0	10.3	8.6	55.2	100.0
Greece	EL652	3	8	5	4	25	45	6.7	17.8	11.1	8.9	55.6	100.0
Greece	EL653	5	17	8	10	46	86	5.8	19.8	9.3	11.6	53.5	100.0
Spain	ES111	23	19	2	32	172	248	9.3	7.7	0.8	12.9	69.4	100.0
Spain	ES112	13	8	1	11	71	104	12.5	7.7	1.0	10.6	68.3	100.0
Spain	ES113	13	8	1	8	65	95	13.7	8.4	1.1	8.4	68.4	100.0

Spain	ES114	23	15	1	22	123	184	12.5	8.2	0.5	12.0	66.8	100.0
Spain	ES120	30	25	2	29	173	259	11.6	9.7	0.8	11.2	66.8	100.0
Spain	ES130	16	14	1	13	80	124	12.9	11.3	0.8	10.5	64.5	100.0
Spain	ES211	10	7	1	5	36	59	16.9	11.9	1.7	8.5	61.0	100.0
Spain	ES212	16	21	2	14	89	142	11.3	14.8	1.4	9.9	62.7	100.0
Spain	ES213	32	31	3	23	151	240	13.3	12.9	1.3	9.6	62.9	100.0
Spain	ES220	17	18	2	11	78	126	13.5	14.3	1.6	8.7	61.9	100.0
Spain	ES230	12	8	1	5	43	69	17.4	11.6	1.4	7.2	62.3	100.0
Spain	ES241	9	8	1	6	38	62	14.5	12.9	1.6	9.7	61.3	100.0
Spain	ES242	6	4	1	4	25	40	15.0	10.0	2.5	10.0	62.5	100.0
Spain	ES243	39	27	4	19	130	219	17.8	12.3	1.8	8.7	59.4	100.0
Spain	ES300	229	95	27	72	740	1,163	19.7	8.2	2.3	6.2	63.6	100.0
Spain	ES411	7	4	1	3	34	49	14.3	8.2	2.0	6.1	69.4	100.0
Spain	ES412	13	8	1	6	56	84	15.5	9.5	1.2	7.1	66.7	100.0
Spain	ES413	20	10	2	8	94	134	14.9	7.5	1.5	6.0	70.1	100.0
Spain	ES414	7	4	1	3	32	47	14.9	8.5	2.1	6.4	68.1	100.0
Spain	ES415	11	8	2	6	62	89	12.4	9.0	2.2	6.7	69.7	100.0
Spain	ES416	6	3	1	2	27	39	15.4	7.7	2.6	5.1	69.2	100.0
Spain	ES417	4	3	1	2	18	28	14.3	10.7	3.6	7.1	64.3	100.0
Spain	ES418	17	9	2	7	77	112	15.2	8.0	1.8	6.3	68.8	100.0
Spain	ES419	8	6	1	4	43	62	12.9	9.7	1.6	6.5	69.4	100.0
Spain	ES421	13	10	3	10	55	91	14.3	11.0	3.3	11.0	60.4	100.0
Spain	ES422	19	12	5	11	86	133	14.3	9.0	3.8	8.3	64.7	100.0
Spain	ES423	9	6	2	6	38	61	14.8	9.8	3.3	9.8	62.3	100.0
Spain	ES424	9	4	1	4	33	51	17.6	7.8	2.0	7.8	64.7	100.0
Spain	ES425	25	13	4	10	96	148	16.9	8.8	2.7	6.8	64.9	100.0
Spain	ES431	19	19	5	14	99	156	12.2	12.2	3.2	9.0	63.5	100.0
Spain	ES432	14	11	3	8	69	105	13.3	10.5	2.9	7.6	65.7	100.0
Spain	ES511	143	208	24	174	590	1,139	12.6	18.3	2.1	15.3	51.8	100.0
Spain	ES512	17	29	3	18	82	149	11.4	19.5	2.0	12.1	55.0	100.0
Spain	ES513	14	14	2	10	57	97	14.4	14.4	2.1	10.3	58.8	100.0
Spain	ES514	20	28	4	24	88	164	12.2	17.1	2.4	14.6	53.7	100.0
Spain	ES521	45	60	16	63	215	399	11.3	15.0	4.0	15.8	53.9	100.0
Spain	ES522	18	19	3	20	69	129	14.0	14.7	2.3	15.5	53.5	100.0
Spain	ES523	81	82	16	93	296	568	14.3	14.4	2.8	16.4	52.1	100.0
Spain	ES531	2	5	1	4	12	24	8.3	20.8	4.2	16.7	50.0	100.0
Spain	ES532	14	33	6	23	89	165	8.5	20.0	3.6	13.9	53.9	100.0
Spain	ES533	1	4	0	3	8	16	6.3	25.0	0.0	18.8	50.0	100.0
Spain	ES611	16	16	7	21	74	134	11.9	11.9	5.2	15.7	55.2	100.0
Spain	ES612	28	32	14	41	135	250	11.2	12.8	5.6	16.4	54.0	100.0

Spain	ES613	31	18	8	21	115	193	16.1	9.3	4.1	10.9	59.6	100.0
Spain	ES614	28	17	11	24	131	211	13.3	8.1	5.2	11.4	62.1	100.0
Spain	ES615	13	13	5	14	62	107	12.1	12.1	4.7	13.1	57.9	100.0
Spain	ES616	26	14	7	16	102	165	15.8	8.5	4.2	9.7	61.8	100.0
Spain	ES617	40	38	20	55	186	339	11.8	11.2	5.9	16.2	54.9	100.0
Spain	ES618	67	40	18	47	225	397	16.9	10.1	4.5	11.8	56.7	100.0
Spain	ES620	35	38	12	43	150	278	12.6	13.7	4.3	15.5	54.0	100.0
Finland	FI193	2	6	2	3	33	46	4.3	13.0	4.3	6.5	71.7	100.0
Finland	FI194	1	5	1	2	24	33	3.0	15.2	3.0	6.1	72.7	100.0
Finland	FI195	1	4	1	2	21	29	3.4	13.8	3.4	6.9	72.4	100.0
Finland	FI196	2	8	2	4	32	48	4.2	16.7	4.2	8.3	66.7	100.0
Finland	FI197	4	12	3	6	55	80	5.0	15.0	3.8	7.5	68.8	100.0
Finland	FI1B1	10	37	10	17	142	216	4.6	17.1	4.6	7.9	65.7	100.0
Finland	FI1C1	3	16	3	8	58	88	3.4	18.2	3.4	9.1	65.9	100.0
Finland	FI1C2	2	5	1	2	21	31	6.5	16.1	3.2	6.5	67.7	100.0
Finland	FI1C3	2	6	2	3	25	38	5.3	15.8	5.3	7.9	65.8	100.0
Finland	FI1C4	2	6	2	3	26	39	5.1	15.4	5.1	7.7	66.7	100.0
Finland	FI1C5	1	4	2	2	19	28	3.6	14.3	7.1	7.1	67.9	100.0
Finland	FI1D1	2	5	2	2	24	35	5.7	14.3	5.7	5.7	68.6	100.0
Finland	FI1D2	2	5	2	3	31	43	4.7	11.6	4.7	7.0	72.1	100.0
Finland	FI1D3	1	3	2	2	22	30	3.3	10.0	6.7	6.7	73.3	100.0
Finland	FI1D5	0	1	0	1	7	9	0.0	11.1	0.0	11.1	77.8	100.0
Finland	FI1D7	1	3	1	1	23	29	3.4	10.3	3.4	3.4	79.3	100.0
Finland	FI1D8	1	2	1	1	11	16	6.3	12.5	6.3	6.3	68.8	100.0
Finland	FI1D9	2	5	2	3	37	49	4.1	10.2	4.1	6.1	75.5	100.0
Finland	FI200	0	1	0	1	3	5	0.0	20.0	0.0	20.0	60.0	100.0
France	FR101	58	52	4	23	209	346	16.8	15.0	1.2	6.6	60.4	100.0
France	FR102	27	24	2	10	94	157	17.2	15.3	1.3	6.4	59.9	100.0
France	FR103	26	23	2	11	94	156	16.7	14.7	1.3	7.1	60.3	100.0
France	FR104	25	22	2	10	90	149	16.8	14.8	1.3	6.7	60.4	100.0
France	FR105	34	31	2	14	123	204	16.7	15.2	1.0	6.9	60.3	100.0
France	FR106	26	24	2	11	93	156	16.7	15.4	1.3	7.1	59.6	100.0
France	FR107	32	28	2	13	113	188	17.0	14.9	1.1	6.9	60.1	100.0
France	FR108	24	22	2	10	87	145	16.6	15.2	1.4	6.9	60.0	100.0
France	FRB01	10	9	1	4	40	64	15.6	14.1	1.6	6.3	62.5	100.0
France	FRB02	11	10	1	5	45	72	15.3	13.9	1.4	6.9	62.5	100.0
France	FRB03	8	7	1	4	34	54	14.8	13.0	1.9	7.4	63.0	100.0
France	FRB04	17	15	1	8	71	112	15.2	13.4	0.9	7.1	63.4	100.0
France	FRB05	10	9	1	5	41	66	15.2	13.6	1.5	7.6	62.1	100.0
France	FRB06	19	17	1	8	70	115	16.5	14.8	0.9	7.0	60.9	100.0

France	FRC11	19	19	2	6	62	108	17.6	17.6	1.9	5.6	57.4	100.0
France	FRC12	9	8	1	3	33	54	16.7	14.8	1.9	5.6	61.1	100.0
France	FRC13	23	20	2	7	68	120	19.2	16.7	1.7	5.8	56.7	100.0
France	FRC14	12	12	1	5	45	75	16.0	16.0	1.3	6.7	60.0	100.0
France	FRC21	18	21	1	5	56	101	17.8	20.8	1.0	5.0	55.4	100.0
France	FRC22	9	9	1	3	27	49	18.4	18.4	2.0	6.1	55.1	100.0
France	FRC23	8	8	1	2	24	43	18.6	18.6	2.3	4.7	55.8	100.0
France	FRC24	6	7	0	2	18	33	18.2	21.2	0.0	6.1	54.5	100.0
France	FRD11	13	20	1	13	95	142	9.2	14.1	0.7	9.2	66.9	100.0
France	FRD12	8	14	1	10	70	103	7.8	13.6	1.0	9.7	68.0	100.0
France	FRD13	7	8	0	5	36	56	12.5	14.3	0.0	8.9	64.3	100.0
France	FRD21	11	12	1	6	51	81	13.6	14.8	1.2	7.4	63.0	100.0
France	FRD22	27	38	2	21	151	239	11.3	15.9	0.8	8.8	63.2	100.0
France	FRE11	48	96	5	35	274	458	10.5	21.0	1.1	7.6	59.8	100.0
France	FRE12	27	53	3	23	169	275	9.8	19.3	1.1	8.4	61.5	100.0
France	FRE21	15	19	1	7	60	102	14.7	18.6	1.0	6.9	58.8	100.0
France	FRE22	17	19	1	8	68	113	15.0	16.8	0.9	7.1	60.2	100.0
France	FRE23	14	21	1	10	72	118	11.9	17.8	0.8	8.5	61.0	100.0
France	FRF11	32	48	2	9	98	189	16.9	25.4	1.1	4.8	51.9	100.0
France	FRF12	25	33	2	7	75	142	17.6	23.2	1.4	4.9	52.8	100.0
France	FRF21	7	11	1	3	29	51	13.7	21.6	2.0	5.9	56.9	100.0
France	FRF22	10	10	1	4	34	59	16.9	16.9	1.7	6.8	57.6	100.0
France	FRF23	18	21	1	7	66	113	15.9	18.6	0.9	6.2	58.4	100.0
France	FRF24	6	7	0	2	22	37	16.2	18.9	0.0	5.4	59.5	100.0
France	FRF31	23	30	2	8	80	143	16.1	21.0	1.4	5.6	55.9	100.0
France	FRF32	6	7	0	2	20	35	17.1	20.0	0.0	5.7	57.1	100.0
France	FRF33	30	47	3	10	110	200	15.0	23.5	1.5	5.0	55.0	100.0
France	FRF34	14	17	1	4	45	81	17.3	21.0	1.2	4.9	55.6	100.0
France	FRG01	29	26	2	22	155	234	12.4	11.1	0.9	9.4	66.2	100.0
France	FRG02	20	17	1	12	91	141	14.2	12.1	0.7	8.5	64.5	100.0
France	FRG03	7	6	0	4	34	51	13.7	11.8	0.0	7.8	66.7	100.0
France	FRG04	15	14	1	8	68	106	14.2	13.2	0.9	7.5	64.2	100.0
France	FRG05	17	15	1	13	86	132	12.9	11.4	0.8	9.8	65.2	100.0
France	FRH01	11	15	1	14	92	133	8.3	11.3	0.8	10.5	69.2	100.0
France	FRH02	14	20	2	24	148	208	6.7	9.6	1.0	11.5	71.2	100.0
France	FRH03	19	20	2	16	114	171	11.1	11.7	1.2	9.4	66.7	100.0
France	FRH04	15	17	1	16	110	159	9.4	10.7	0.6	10.1	69.2	100.0
France	FRI11	16	13	1	7	61	98	16.3	13.3	1.0	7.1	62.2	100.0
France	FRI12	43	37	3	25	183	291	14.8	12.7	1.0	8.6	62.9	100.0
France	FRI13	10	11	1	7	50	79	12.7	13.9	1.3	8.9	63.3	100.0

France	FRI14	12	10	1	6	47	76	15.8	13.2	1.3	7.9	61.8	100.0
France	FRI15	19	22	2	12	94	149	12.8	14.8	1.3	8.1	63.1	100.0
France	FRI21	9	9	1	4	39	62	14.5	14.5	1.6	6.5	62.9	100.0
France	FRI22	5	5	0	2	21	33	15.2	15.2	0.0	6.1	63.6	100.0
France	FRI23	14	13	1	7	56	91	15.4	14.3	1.1	7.7	61.5	100.0
France	FRI31	12	10	1	6	50	79	15.2	12.7	1.3	7.6	63.3	100.0
France	FRI32	19	18	2	14	97	150	12.7	12.0	1.3	9.3	64.7	100.0
France	FRI33	10	9	1	6	46	72	13.9	12.5	1.4	8.3	63.9	100.0
France	FRI34	13	11	1	7	56	88	14.8	12.5	1.1	8.0	63.6	100.0
France	FRJ11	14	15	1	9	52	91	15.4	16.5	1.1	9.9	57.1	100.0
France	FRJ12	27	29	3	14	89	162	16.7	17.9	1.9	8.6	54.9	100.0
France	FRJ13	38	46	4	27	149	264	14.4	17.4	1.5	10.2	56.4	100.0
France	FRJ14	3	4	0	1	13	21	14.3	19.0	0.0	4.8	61.9	100.0
France	FRJ15	17	23	2	13	75	130	13.1	17.7	1.5	10.0	57.7	100.0
France	FRJ21	5	5	1	3	22	36	13.9	13.9	2.8	8.3	61.1	100.0
France	FRJ22	10	12	1	5	44	72	13.9	16.7	1.4	6.9	61.1	100.0
France	FRJ23	38	32	3	19	129	221	17.2	14.5	1.4	8.6	58.4	100.0
France	FRJ24	6	5	0	3	24	38	15.8	13.2	0.0	7.9	63.2	100.0
France	FRJ25	6	6	1	3	26	42	14.3	14.3	2.4	7.1	61.9	100.0
France	FRJ26	8	9	1	4	37	59	13.6	15.3	1.7	6.8	62.7	100.0
France	FRJ27	13	14	1	7	50	85	15.3	16.5	1.2	8.2	58.8	100.0
France	FRJ28	8	7	1	4	30	50	16.0	14.0	2.0	8.0	60.0	100.0
France	FRK11	14	12	1	5	51	83	16.9	14.5	1.2	6.0	61.4	100.0
France	FRK12	6	7	1	3	27	44	13.6	15.9	2.3	6.8	61.4	100.0
France	FRK13	7	8	1	3	29	48	14.6	16.7	2.1	6.3	60.4	100.0
France	FRK14	21	20	2	8	82	133	15.8	15.0	1.5	6.0	61.7	100.0
France	FRK21	19	16	1	5	50	91	20.9	17.6	1.1	5.5	54.9	100.0
France	FRK22	15	13	1	5	42	76	19.7	17.1	1.3	6.6	55.3	100.0
France	FRK23	22	18	2	7	54	103	21.4	17.5	1.9	6.8	52.4	100.0
France	FRK24	39	33	3	11	106	192	20.3	17.2	1.6	5.7	55.2	100.0
France	FRK25	28	25	3	10	94	160	17.5	15.6	1.9	6.3	58.8	100.0
France	FRK26	64	49	5	18	167	303	21.1	16.2	1.7	5.9	55.1	100.0
France	FRK27	15	15	1	4	48	83	18.1	18.1	1.2	4.8	57.8	100.0
France	FRK28	21	26	2	7	74	130	16.2	20.0	1.5	5.4	56.9	100.0
France	FRL01	6	9	1	3	25	44	13.6	20.5	2.3	6.8	56.8	100.0
France	FRL02	4	6	1	2	23	36	11.1	16.7	2.8	5.6	63.9	100.0
France	FRL03	36	79	7	41	158	321	11.2	24.6	2.2	12.8	49.2	100.0
France	FRL04	75	96	9	48	238	466	16.1	20.6	1.9	10.3	51.1	100.0
France	FRL05	39	65	6	32	148	290	13.4	22.4	2.1	11.0	51.0	100.0
France	FRL06	26	27	3	11	73	140	18.6	19.3	2.1	7.9	52.1	100.0

France	FRM01	4	8	1	5	19	37	10.8	21.6	2.7	13.5	51.4	100.0
France	FRM02	4	11	1	6	22	44	9.1	25.0	2.3	13.6	50.0	100.0
Croatia	HR	62	411	67	71	620	1,231	5.0	33.4	5.4	5.8	50.4	100.0
Hungary	HU110	39	160	25	19	253	496	7.9	32.3	5.0	3.8	51.0	100.0
Hungary	HU120	25	104	17	12	165	323	7.7	32.2	5.3	3.7	51.1	100.0
Hungary	HU211	8	39	6	5	61	119	6.7	32.8	5.0	4.2	51.3	100.0
Hungary	HU212	5	31	4	4	46	90	5.6	34.4	4.4	4.4	51.1	100.0
Hungary	HU213	6	38	5	4	54	107	5.6	35.5	4.7	3.7	50.5	100.0
Hungary	HU221	5	43	5	5	58	116	4.3	37.1	4.3	4.3	50.0	100.0
Hungary	HU222	3	28	3	3	37	74	4.1	37.8	4.1	4.1	50.0	100.0
Hungary	HU223	4	30	4	4	41	83	4.8	36.1	4.8	4.8	49.4	100.0
Hungary	HU231	6	37	6	5	57	111	5.4	33.3	5.4	4.5	51.4	100.0
Hungary	HU232	5	34	5	4	50	98	5.1	34.7	5.1	4.1	51.0	100.0
Hungary	HU233	4	22	4	3	35	68	5.9	32.4	5.9	4.4	51.5	100.0
Hungary	HU311	11	63	12	7	103	196	5.6	32.1	6.1	3.6	52.6	100.0
Hungary	HU312	6	29	5	4	48	92	6.5	31.5	5.4	4.3	52.2	100.0
Hungary	HU313	4	21	3	2	33	63	6.3	33.3	4.8	3.2	52.4	100.0
Hungary	HU321	7	40	9	5	71	132	5.3	30.3	6.8	3.8	53.8	100.0
Hungary	HU322	8	37	7	5	62	119	6.7	31.1	5.9	4.2	52.1	100.0
Hungary	HU323	6	41	11	5	74	137	4.4	29.9	8.0	3.6	54.0	100.0
Hungary	HU331	10	48	9	6	77	150	6.7	32.0	6.0	4.0	51.3	100.0
Hungary	HU332	6	36	8	4	62	116	5.2	31.0	6.9	3.4	53.4	100.0
Hungary	HU333	6	38	8	5	61	118	5.1	32.2	6.8	4.2	51.7	100.0
Ireland	IE041	1	4	0	2	38	45	2.2	8.9	0.0	4.4	84.4	100.0
Ireland	IE042	1	4	0	2	43	50	2.0	8.0	0.0	4.0	86.0	100.0
Ireland	IE051	2	5	0	3	46	56	3.6	8.9	0.0	5.4	82.1	100.0
Ireland	IE052	2	5	0	3	39	49	4.1	10.2	0.0	6.1	79.6	100.0
Ireland	IE053	2	7	1	5	69	84	2.4	8.3	1.2	6.0	82.1	100.0
Ireland	IE061	6	14	1	8	104	133	4.5	10.5	0.8	6.0	78.2	100.0
Ireland	IE062	3	6	0	3	45	57	5.3	10.5	0.0	5.3	78.9	100.0
Ireland	IE063	1	3	0	2	25	31	3.2	9.7	0.0	6.5	80.6	100.0
Iceland	IS001	0	1	0	1	20	22	0.0	4.5	0.0	4.5	90.9	100.0
Iceland	IS002	0	1	0	1	14	16	0.0	6.3	0.0	6.3	87.5	100.0
Italy	ITC11	183	113	11	36	309	652	28.1	17.3	1.7	5.5	47.4	100.0
Italy	ITC12	16	12	1	4	28	61	26.2	19.7	1.6	6.6	45.9	100.0
Italy	ITC13	16	11	1	3	31	62	25.8	17.7	1.6	4.8	50.0	100.0
Italy	ITC14	12	11	1	3	27	54	22.2	20.4	1.9	5.6	50.0	100.0
Italy	ITC15	26	20	2	6	47	101	25.7	19.8	2.0	5.9	46.5	100.0
Italy	ITC16	40	32	3	11	88	174	23.0	18.4	1.7	6.3	50.6	100.0
Italy	ITC17	18	14	1	6	35	74	24.3	18.9	1.4	8.1	47.3	100.0

Italy	ITC18	33	34	3	16	74	160	20.6	21.3	1.9	10.0	46.3	100.0
Italy	ITC20	4	7	1	2	25	39	10.3	17.9	2.6	5.1	64.1	100.0
Italy	ITC31	11	18	2	10	38	79	13.9	22.8	2.5	12.7	48.1	100.0
Italy	ITC32	17	23	2	13	50	105	16.2	21.9	1.9	12.4	47.6	100.0
Italy	ITC33	49	74	8	45	148	324	15.1	22.8	2.5	13.9	45.7	100.0
Italy	ITC34	11	18	2	10	36	77	14.3	23.4	2.6	13.0	46.8	100.0
Italy	ITC41	60	48	5	14	110	237	25.3	20.3	2.1	5.9	46.4	100.0
Italy	ITC42	42	33	3	9	75	162	25.9	20.4	1.9	5.6	46.3	100.0
Italy	ITC43	24	17	2	5	41	89	27.0	19.1	2.2	5.6	46.1	100.0
Italy	ITC44	9	10	1	2	29	51	17.6	19.6	2.0	3.9	56.9	100.0
Italy	ITC46	73	51	6	15	122	267	27.3	19.1	2.2	5.6	45.7	100.0
Italy	ITC47	72	58	7	16	133	286	25.2	20.3	2.4	5.6	46.5	100.0
Italy	ITC48	40	35	4	13	79	171	23.4	20.5	2.3	7.6	46.2	100.0
Italy	ITC49	13	11	1	4	25	54	24.1	20.4	1.9	7.4	46.3	100.0
Italy	ITC4A	23	20	2	6	44	95	24.2	21.1	2.1	6.3	46.3	100.0
Italy	ITC4B	25	23	3	7	49	107	23.4	21.5	2.8	6.5	45.8	100.0
Italy	ITC4C	209	164	17	55	373	818	25.6	20.0	2.1	6.7	45.6	100.0
Italy	ITC4D	55	41	4	12	94	206	26.7	19.9	1.9	5.8	45.6	100.0
Italy	ITF11	12	19	4	8	50	93	12.9	20.4	4.3	8.6	53.8	100.0
Italy	ITF12	11	19	4	8	44	86	12.8	22.1	4.7	9.3	51.2	100.0
Italy	ITF13	12	19	4	8	46	89	13.5	21.3	4.5	9.0	51.7	100.0
Italy	ITF14	16	26	6	12	61	121	13.2	21.5	5.0	9.9	50.4	100.0
Italy	ITF21	4	6	1	3	15	29	13.8	20.7	3.4	10.3	51.7	100.0
Italy	ITF22	9	15	4	7	36	71	12.7	21.1	5.6	9.9	50.7	100.0
Italy	ITF31	32	42	9	27	103	213	15.0	19.7	4.2	12.7	48.4	100.0
Italy	ITF32	14	18	4	9	43	88	15.9	20.5	4.5	10.2	48.9	100.0
Italy	ITF33	109	139	31	95	340	714	15.3	19.5	4.3	13.3	47.6	100.0
Italy	ITF34	20	25	6	14	62	127	15.7	19.7	4.7	11.0	48.8	100.0
Italy	ITF35	40	56	14	36	141	287	13.9	19.5	4.9	12.5	49.1	100.0
Italy	ITF43	19	34	10	17	76	156	12.2	21.8	6.4	10.9	48.7	100.0
Italy	ITF44	11	25	7	13	53	109	10.1	22.9	6.4	11.9	48.6	100.0
Italy	ITF45	23	53	16	30	113	235	9.8	22.6	6.8	12.8	48.1	100.0
Italy	ITF46	20	36	9	17	81	163	12.3	22.1	5.5	10.4	49.7	100.0
Italy	ITF47	34	68	19	35	149	305	11.1	22.3	6.2	11.5	48.9	100.0
Italy	ITF48	10	19	5	9	43	86	11.6	22.1	5.8	10.5	50.0	100.0
Italy	ITF51	13	22	6	11	58	110	11.8	20.0	5.5	10.0	52.7	100.0
Italy	ITF52	7	11	3	5	29	55	12.7	20.0	5.5	9.1	52.7	100.0
Italy	ITF61	21	37	11	23	101	193	10.9	19.2	5.7	11.9	52.3	100.0
Italy	ITF62	5	8	2	5	22	42	11.9	19.0	4.8	11.9	52.4	100.0
Italy	ITF63	10	17	5	13	47	92	10.9	18.5	5.4	14.1	51.1	100.0

Italy	ITF64	5	8	2	7	21	43	11.6	18.6	4.7	16.3	48.8	100.0
Italy	ITF65	16	27	8	23	72	146	11.0	18.5	5.5	15.8	49.3	100.0
Italy	ITG11	12	25	7	20	62	126	9.5	19.8	5.6	15.9	49.2	100.0
Italy	ITG12	33	61	17	48	162	321	10.3	19.0	5.3	15.0	50.5	100.0
Italy	ITG13	21	36	10	32	96	195	10.8	18.5	5.1	16.4	49.2	100.0
Italy	ITG14	13	25	7	20	68	133	9.8	18.8	5.3	15.0	51.1	100.0
Italy	ITG15	8	14	4	12	41	79	10.1	17.7	5.1	15.2	51.9	100.0
Italy	ITG16	6	10	3	7	30	56	10.7	17.9	5.4	12.5	53.6	100.0
Italy	ITG17	32	48	15	39	148	282	11.3	17.0	5.3	13.8	52.5	100.0
Italy	ITG18	8	14	5	15	39	81	9.9	17.3	6.2	18.5	48.1	100.0
Italy	ITG19	13	19	6	18	55	111	11.7	17.1	5.4	16.2	49.5	100.0
Italy	ITG2D	11	30	4	16	63	124	8.9	24.2	3.2	12.9	50.8	100.0
Italy	ITG2E	5	14	2	7	32	60	8.3	23.3	3.3	11.7	53.3	100.0
Italy	ITG2F	9	22	5	13	50	99	9.1	22.2	5.1	13.1	50.5	100.0
Italy	ITG2G	4	11	2	5	24	46	8.7	23.9	4.3	10.9	52.2	100.0
Italy	ITG2H	9	23	4	13	51	100	9.0	23.0	4.0	13.0	51.0	100.0
Italy	ITH10	14	22	2	4	58	100	14.0	22.0	2.0	4.0	58.0	100.0
Italy	ITH20	24	26	3	6	64	123	19.5	21.1	2.4	4.9	52.0	100.0
Italy	ITH31	52	49	6	13	103	223	23.3	22.0	2.7	5.8	46.2	100.0
Italy	ITH32	44	44	5	11	91	195	22.6	22.6	2.6	5.6	46.7	100.0
Italy	ITH33	12	15	2	3	33	65	18.5	23.1	3.1	4.6	50.8	100.0
Italy	ITH34	39	46	5	12	91	193	20.2	23.8	2.6	6.2	47.2	100.0
Italy	ITH35	40	54	7	17	104	222	18.0	24.3	3.2	7.7	46.8	100.0
Italy	ITH36	45	50	6	14	100	215	20.9	23.3	2.8	6.5	46.5	100.0
Italy	ITH37	13	17	2	5	34	71	18.3	23.9	2.8	7.0	47.9	100.0
Italy	ITH41	13	19	2	5	36	75	17.3	25.3	2.7	6.7	48.0	100.0
Italy	ITH42	22	41	5	10	74	152	14.5	27.0	3.3	6.6	48.7	100.0
Italy	ITH43	6	13	1	3	22	45	13.3	28.9	2.2	6.7	48.9	100.0
Italy	ITH44	8	25	3	6	40	82	9.8	30.5	3.7	7.3	48.8	100.0
Italy	ITH51	20	18	2	6	41	87	23.0	20.7	2.3	6.9	47.1	100.0
Italy	ITH52	28	27	3	9	59	126	22.2	21.4	2.4	7.1	46.8	100.0
Italy	ITH53	29	29	3	10	62	133	21.8	21.8	2.3	7.5	46.6	100.0
Italy	ITH54	38	39	5	13	84	179	21.2	21.8	2.8	7.3	46.9	100.0
Italy	ITH55	55	65	9	23	138	290	19.0	22.4	3.1	7.9	47.6	100.0
Italy	ITH56	22	28	4	10	57	121	18.2	23.1	3.3	8.3	47.1	100.0
Italy	ITH57	18	28	4	11	56	117	15.4	23.9	3.4	9.4	47.9	100.0
Italy	ITH58	15	25	4	10	52	106	14.2	23.6	3.8	9.4	49.1	100.0
Italy	ITH59	11	20	3	8	41	83	13.3	24.1	3.6	9.6	49.4	100.0
Italy	ITI11	10	15	2	8	31	66	15.2	22.7	3.0	12.1	47.0	100.0
Italy	ITI12	19	29	4	15	60	127	15.0	22.8	3.1	11.8	47.2	100.0

Italy	ITI13	13	19	2	9	40	83	15.7	22.9	2.4	10.8	48.2	100.0
Italy	ITI14	46	65	9	30	139	289	15.9	22.5	3.1	10.4	48.1	100.0
Italy	ITI15	10	15	2	6	30	63	15.9	23.8	3.2	9.5	47.6	100.0
Italy	ITI16	14	27	3	17	53	114	12.3	23.7	2.6	14.9	46.5	100.0
Italy	ITI17	17	27	3	15	55	117	14.5	23.1	2.6	12.8	47.0	100.0
Italy	ITI18	13	22	3	9	49	96	13.5	22.9	3.1	9.4	51.0	100.0
Italy	ITI19	11	19	3	9	43	85	12.9	22.4	3.5	10.6	50.6	100.0
Italy	ITI1A	8	17	3	10	36	74	10.8	23.0	4.1	13.5	48.6	100.0
Italy	ITI21	24	41	7	17	94	183	13.1	22.4	3.8	9.3	51.4	100.0
Italy	ITI22	10	15	3	7	38	73	13.7	20.5	4.1	9.6	52.1	100.0
Italy	ITI31	12	24	4	9	49	98	12.2	24.5	4.1	9.2	50.0	100.0
Italy	ITI32	17	34	6	15	71	143	11.9	23.8	4.2	10.5	49.7	100.0
Italy	ITI33	12	23	4	9	49	97	12.4	23.7	4.1	9.3	50.5	100.0
Italy	ITI34	8	15	3	6	32	64	12.5	23.4	4.7	9.4	50.0	100.0
Italy	ITI35	6	12	2	6	27	53	11.3	22.6	3.8	11.3	50.9	100.0
Italy	ITI41	12	20	4	11	47	94	12.8	21.3	4.3	11.7	50.0	100.0
Italy	ITI42	8	11	2	5	27	53	15.1	20.8	3.8	9.4	50.9	100.0
Italy	ITI43	164	216	42	135	534	1,091	15.0	19.8	3.8	12.4	48.9	100.0
Italy	ITI44	19	27	6	19	67	138	13.8	19.6	4.3	13.8	48.6	100.0
Italy	ITI45	21	27	6	15	74	143	14.7	18.9	4.2	10.5	51.7	100.0
Liechtenstein	LI000	0	2	0	0	3	5	0.0	40.0	0.0	0.0	60.0	100.0
Lithuania	LT011	7	39	17	11	110	184	3.8	21.2	9.2	6.0	59.8	100.0
Lithuania	LT021	1	10	4	3	26	44	2.3	22.7	9.1	6.8	59.1	100.0
Lithuania	LT022	6	34	12	10	90	152	3.9	22.4	7.9	6.6	59.2	100.0
Lithuania	LT023	2	21	5	8	51	87	2.3	24.1	5.7	9.2	58.6	100.0
Lithuania	LT024	1	10	3	3	25	42	2.4	23.8	7.1	7.1	59.5	100.0
Lithuania	LT025	3	14	5	4	40	66	4.5	21.2	7.6	6.1	60.6	100.0
Lithuania	LT026	3	18	5	6	47	79	3.8	22.8	6.3	7.6	59.5	100.0
Lithuania	LT027	1	7	2	2	18	30	3.3	23.3	6.7	6.7	60.0	100.0
Lithuania	LT028	1	9	2	3	21	36	2.8	25.0	5.6	8.3	58.3	100.0
Lithuania	LT029	2	10	4	3	28	47	4.3	21.3	8.5	6.4	59.6	100.0
Luxembourg	LU000	1	30	1	4	46	82	1.2	36.6	1.2	4.9	56.1	100.0
Latvia	LV003	1	18	4	7	44	74	1.4	24.3	5.4	9.5	59.5	100.0
Latvia	LV005	2	16	7	5	48	78	2.6	20.5	9.0	6.4	61.5	100.0
Latvia	LV006	6	37	10	13	102	168	3.6	22.0	6.0	7.7	60.7	100.0
Latvia	LV007	3	19	5	7	54	88	3.4	21.6	5.7	8.0	61.4	100.0
Latvia	LV008	2	12	4	4	39	61	3.3	19.7	6.6	6.6	63.9	100.0
Latvia	LV009	2	12	4	4	34	56	3.6	21.4	7.1	7.1	60.7	100.0
Montenegro	ME000	3	43	12	9	87	154	1.9	27.9	7.8	5.8	56.5	100.0
Malta	MT	1	23	5	21	37	87	1.1	26.4	5.7	24.1	42.5	100.0

Netherlands	NL11	5	29	2	9	65	110	4.5	26.4	1.8	8.2	59.1	100.0
Netherlands	NL12	6	32	2	10	75	125	4.8	25.6	1.6	8.0	60.0	100.0
Netherlands	NL13	6	28	1	8	59	102	5.9	27.5	1.0	7.8	57.8	100.0
Netherlands	NL21	13	56	2	14	111	196	6.6	28.6	1.0	7.1	56.6	100.0
Netherlands	NL22	26	101	4	23	203	357	7.3	28.3	1.1	6.4	56.9	100.0
Netherlands	NL23	3	13	1	4	28	49	6.1	26.5	2.0	8.2	57.1	100.0
Netherlands	NL31	13	50	2	13	112	190	6.8	26.3	1.1	6.8	58.9	100.0
Netherlands	NL32	24	122	6	37	283	472	5.1	25.8	1.3	7.8	60.0	100.0
Netherlands	NL33	29	147	7	43	344	570	5.1	25.8	1.2	7.5	60.4	100.0
Netherlands	NL34	2	20	1	5	45	73	2.7	27.4	1.4	6.8	61.6	100.0
Netherlands	NL41	26	132	5	28	251	442	5.9	29.9	1.1	6.3	56.8	100.0
Netherlands	NL42	10	80	3	14	131	238	4.2	33.6	1.3	5.9	55.0	100.0
Norway	NO020	3	8	1	4	49	65	4.6	12.3	1.5	6.2	75.4	100.0
Norway	NO060	3	6	1	4	46	60	5.0	10.0	1.7	6.7	76.7	100.0
Norway	NO071	1	3	1	2	30	37	2.7	8.1	2.7	5.4	81.1	100.0
Norway	NO074	1	2	1	2	25	31	3.2	6.5	3.2	6.5	80.6	100.0
Norway	NO081	4	9	1	5	47	66	6.1	13.6	1.5	7.6	71.2	100.0
Norway	NO082	8	22	3	11	107	151	5.3	14.6	2.0	7.3	70.9	100.0
Norway	NO091	3	10	1	5	46	65	4.6	15.4	1.5	7.7	70.8	100.0
Norway	NO092	1	7	1	4	31	44	2.3	15.9	2.3	9.1	70.5	100.0
Norway	NO0A1	2	9	1	5	40	57	3.5	15.8	1.8	8.8	70.2	100.0
Norway	NO0A2	2	5	1	4	33	45	4.4	11.1	2.2	8.9	73.3	100.0
Norway	NO0A3	1	4	1	3	31	40	2.5	10.0	2.5	7.5	77.5	100.0
Poland	PL213	23	40	7	6	80	156	14.7	25.6	4.5	3.8	51.3	100.0
Poland	PL214	20	36	7	5	71	139	14.4	25.9	5.0	3.6	51.1	100.0
Poland	PL217	12	21	5	3	43	84	14.3	25.0	6.0	3.6	51.2	100.0
Poland	PL218	11	25	5	4	48	93	11.8	26.9	5.4	4.3	51.6	100.0
Poland	PL219	7	18	3	3	34	65	10.8	27.7	4.6	4.6	52.3	100.0
Poland	PL21A	16	33	5	5	62	121	13.2	27.3	4.1	4.1	51.2	100.0
Poland	PL224	18	34	6	6	68	132	13.6	25.8	4.5	4.5	51.5	100.0
Poland	PL225	16	44	6	6	75	147	10.9	29.9	4.1	4.1	51.0	100.0
Poland	PL227	15	39	5	5	66	130	11.5	30.0	3.8	3.8	50.8	100.0
Poland	PL228	14	29	4	4	54	105	13.3	27.6	3.8	3.8	51.4	100.0
Poland	PL229	14	29	4	4	52	103	13.6	28.2	3.9	3.9	50.5	100.0
Poland	PL22A	27	54	8	8	101	198	13.6	27.3	4.0	4.0	51.0	100.0
Poland	PL22B	25	48	8	7	93	181	13.8	26.5	4.4	3.9	51.4	100.0
Poland	PL22C	10	21	3	3	39	76	13.2	27.6	3.9	3.9	51.3	100.0
Poland	PL411	9	19	3	5	45	81	11.1	23.5	3.7	6.2	55.6	100.0
Poland	PL414	17	33	5	7	71	133	12.8	24.8	3.8	5.3	53.4	100.0
Poland	PL415	14	31	4	7	65	121	11.6	25.6	3.3	5.8	53.7	100.0

Poland	PL416	17	37	5	7	75	141	12.1	26.2	3.5	5.0	53.2	100.0
Poland	PL417	11	28	3	5	55	102	10.8	27.5	2.9	4.9	53.9	100.0
Poland	PL418	12	27	3	6	56	104	11.5	26.0	2.9	5.8	53.8	100.0
Poland	PL424	7	24	2	7	52	92	7.6	26.1	2.2	7.6	56.5	100.0
Poland	PL426	5	17	2	6	42	72	6.9	23.6	2.8	8.3	58.3	100.0
Poland	PL427	8	23	3	6	52	92	8.7	25.0	3.3	6.5	56.5	100.0
Poland	PL428	7	26	3	7	55	98	7.1	26.5	3.1	7.1	56.1	100.0
Poland	PL431	7	22	2	5	42	78	9.0	28.2	2.6	6.4	53.8	100.0
Poland	PL432	11	39	3	7	68	128	8.6	30.5	2.3	5.5	53.1	100.0
Poland	PL514	15	41	4	6	73	139	10.8	29.5	2.9	4.3	52.5	100.0
Poland	PL515	10	46	4	6	72	138	7.2	33.3	2.9	4.3	52.2	100.0
Poland	PL516	9	27	3	4	48	91	9.9	29.7	3.3	4.4	52.7	100.0
Poland	PL517	14	56	5	8	91	174	8.0	32.2	2.9	4.6	52.3	100.0
Poland	PL518	12	33	4	5	59	113	10.6	29.2	3.5	4.4	52.2	100.0
Poland	PL523	9	27	3	4	47	90	10.0	30.0	3.3	4.4	52.2	100.0
Poland	PL524	16	36	5	6	66	129	12.4	27.9	3.9	4.7	51.2	100.0
Poland	PL613	19	33	6	9	82	149	12.8	22.1	4.0	6.0	55.0	100.0
Poland	PL616	10	17	4	5	44	80	12.5	21.3	5.0	6.3	55.0	100.0
Poland	PL617	9	17	3	4	40	73	12.3	23.3	4.1	5.5	54.8	100.0
Poland	PL618	4	8	1	2	20	35	11.4	22.9	2.9	5.7	57.1	100.0
Poland	PL619	11	18	3	4	45	81	13.6	22.2	3.7	4.9	55.6	100.0
Poland	PL621	12	22	5	7	60	106	11.3	20.8	4.7	6.6	56.6	100.0
Poland	PL622	13	23	7	8	67	118	11.0	19.5	5.9	6.8	56.8	100.0
Poland	PL623	5	10	4	3	31	53	9.4	18.9	7.5	5.7	58.5	100.0
Poland	PL633	14	35	7	14	94	164	8.5	21.3	4.3	8.5	57.3	100.0
Poland	PL634	7	18	4	7	48	84	8.3	21.4	4.8	8.3	57.1	100.0
Poland	PL636	5	14	2	5	37	63	7.9	22.2	3.2	7.9	58.7	100.0
Poland	PL637	4	9	1	3	22	39	10.3	23.1	2.6	7.7	56.4	100.0
Poland	PL638	9	17	3	6	46	81	11.1	21.0	3.7	7.4	56.8	100.0
Poland	PL711	30	49	9	10	109	207	14.5	23.7	4.3	4.8	52.7	100.0
Poland	PL712	14	22	4	4	49	93	15.1	23.7	4.3	4.3	52.7	100.0
Poland	PL713	21	33	6	6	72	138	15.2	23.9	4.3	4.3	52.2	100.0
Poland	PL714	15	27	5	5	57	109	13.8	24.8	4.6	4.6	52.3	100.0
Poland	PL715	13	20	4	4	48	89	14.6	22.5	4.5	4.5	53.9	100.0
Poland	PL721	26	39	9	7	90	171	15.2	22.8	5.3	4.1	52.6	100.0
Poland	PL722	18	28	7	5	62	120	15.0	23.3	5.8	4.2	51.7	100.0
Poland	PL811	7	12	5	3	35	62	11.3	19.4	8.1	4.8	56.5	100.0
Poland	PL812	16	29	14	6	79	144	11.1	20.1	9.7	4.2	54.9	100.0
Poland	PL814	19	28	11	6	77	141	13.5	19.9	7.8	4.3	54.6	100.0
Poland	PL815	15	22	8	5	58	108	13.9	20.4	7.4	4.6	53.7	100.0

Poland	PL821	10	22	7	4	48	91	11.0	24.2	7.7	4.4	52.7	100.0
Poland	PL822	9	17	7	3	41	77	11.7	22.1	9.1	3.9	53.2	100.0
Poland	PL823	15	26	8	4	58	111	13.5	23.4	7.2	3.6	52.3	100.0
Poland	PL824	16	25	7	4	58	110	14.5	22.7	6.4	3.6	52.7	100.0
Poland	PL841	10	17	8	5	54	94	10.6	18.1	8.5	5.3	57.4	100.0
Poland	PL842	10	17	7	5	51	90	11.1	18.9	7.8	5.6	56.7	100.0
Poland	PL843	5	10	4	3	30	52	9.6	19.2	7.7	5.8	57.7	100.0
Poland	PL911	59	83	22	19	213	396	14.9	21.0	5.6	4.8	53.8	100.0
Poland	PL912	17	23	7	6	62	115	14.8	20.0	6.1	5.2	53.9	100.0
Poland	PL913	17	24	6	6	62	115	14.8	20.9	5.2	5.2	53.9	100.0
Poland	PL921	20	29	8	6	72	135	14.8	21.5	5.9	4.4	53.3	100.0
Poland	PL922	10	15	4	4	42	75	13.3	20.0	5.3	5.3	56.0	100.0
Poland	PL923	10	15	3	4	39	71	14.1	21.1	4.2	5.6	54.9	100.0
Poland	PL924	10	15	5	4	45	79	12.7	19.0	6.3	5.1	57.0	100.0
Poland	PL925	12	17	6	4	48	87	13.8	19.5	6.9	4.6	55.2	100.0
Poland	PL926	9	13	3	3	32	60	15.0	21.7	5.0	5.0	53.3	100.0
Portugal	PT111	3	9	1	7	39	59	5.1	15.3	1.7	11.9	66.1	100.0
Portugal	PT112	6	9	1	7	40	63	9.5	14.3	1.6	11.1	63.5	100.0
Portugal	PT119	8	10	1	7	43	69	11.6	14.5	1.4	10.1	62.3	100.0
Portugal	PT11A	37	44	3	40	200	324	11.4	13.6	0.9	12.3	61.7	100.0
Portugal	PT11B	2	4	0	2	19	27	7.4	14.8	0.0	7.4	70.4	100.0
Portugal	PT11C	11	9	1	7	44	72	15.3	12.5	1.4	9.7	61.1	100.0
Portugal	PT11D	7	8	1	4	35	55	12.7	14.5	1.8	7.3	63.6	100.0
Portugal	PT11E	2	6	1	2	25	36	5.6	16.7	2.8	5.6	69.4	100.0
Portugal	PT150	12	17	4	19	74	126	9.5	13.5	3.2	15.1	58.7	100.0
Portugal	PT16B	7	11	1	13	56	88	8.0	12.5	1.1	14.8	63.6	100.0
Portugal	PT16D	9	10	1	10	46	76	11.8	13.2	1.3	13.2	60.5	100.0
Portugal	PT16E	15	15	1	15	71	117	12.8	12.8	0.9	12.8	60.7	100.0
Portugal	PT16F	7	9	1	10	41	68	10.3	13.2	1.5	14.7	60.3	100.0
Portugal	PT16G	11	9	1	6	45	72	15.3	12.5	1.4	8.3	62.5	100.0
Portugal	PT16H	4	4	1	3	20	32	12.5	12.5	3.1	9.4	62.5	100.0
Portugal	PT16I	9	9	1	9	44	72	12.5	12.5	1.4	12.5	61.1	100.0
Portugal	PT16J	10	11	1	6	51	79	12.7	13.9	1.3	7.6	64.6	100.0
Portugal	PT170	63	68	8	80	371	590	10.7	11.5	1.4	13.6	62.9	100.0
Portugal	PT181	4	3	1	4	17	29	13.8	10.3	3.4	13.8	58.6	100.0
Portugal	PT184	5	6	1	5	27	44	11.4	13.6	2.3	11.4	61.4	100.0
Portugal	PT185	8	8	1	8	42	67	11.9	11.9	1.5	11.9	62.7	100.0
Portugal	PT186	5	6	1	4	26	42	11.9	14.3	2.4	9.5	61.9	100.0
Portugal	PT187	6	6	1	5	31	49	12.2	12.2	2.0	10.2	63.3	100.0
Romania	RO111	12	40	12	6	84	154	7.8	26.0	7.8	3.9	54.5	100.0

Romania	RO112	6	14	7	2	41	70	8.6	20.0	10.0	2.9	58.6	100.0
Romania	RO113	16	35	14	6	93	164	9.8	21.3	8.5	3.7	56.7	100.0
Romania	RO114	8	26	12	4	68	118	6.8	22.0	10.2	3.4	57.6	100.0
Romania	RO115	7	21	8	3	48	87	8.0	24.1	9.2	3.4	55.2	100.0
Romania	RO116	5	14	5	2	33	59	8.5	23.7	8.5	3.4	55.9	100.0
Romania	RO121	9	19	8	3	52	91	9.9	20.9	8.8	3.3	57.1	100.0
Romania	RO122	13	23	14	5	76	131	9.9	17.6	10.7	3.8	58.0	100.0
Romania	RO123	5	9	6	2	30	52	9.6	17.3	11.5	3.8	57.7	100.0
Romania	RO124	6	14	8	3	46	77	7.8	18.2	10.4	3.9	59.7	100.0
Romania	RO125	15	26	14	5	81	141	10.6	18.4	9.9	3.5	57.4	100.0
Romania	RO126	10	18	9	3	54	94	10.6	19.1	9.6	3.2	57.4	100.0
Romania	RO211	14	29	22	6	96	167	8.4	17.4	13.2	3.6	57.5	100.0
Romania	RO212	6	21	16	4	63	110	5.5	19.1	14.5	3.6	57.3	100.0
Romania	RO213	11	29	26	6	99	171	6.4	17.0	15.2	3.5	57.9	100.0
Romania	RO214	10	25	17	5	78	135	7.4	18.5	12.6	3.7	57.8	100.0
Romania	RO215	10	30	20	6	89	155	6.5	19.4	12.9	3.9	57.4	100.0
Romania	RO216	7	18	17	4	62	108	6.5	16.7	15.7	3.7	57.4	100.0
Romania	RO221	8	15	16	4	56	99	8.1	15.2	16.2	4.0	56.6	100.0
Romania	RO222	13	23	19	5	79	139	9.4	16.5	13.7	3.6	56.8	100.0
Romania	RO223	12	26	30	11	101	180	6.7	14.4	16.7	6.1	56.1	100.0
Romania	RO224	10	22	22	6	79	139	7.2	15.8	15.8	4.3	56.8	100.0
Romania	RO225	4	9	11	3	36	63	6.3	14.3	17.5	4.8	57.1	100.0
Romania	RO226	8	16	13	4	55	96	8.3	16.7	13.5	4.2	57.3	100.0
Romania	RO311	20	28	17	6	91	162	12.3	17.3	10.5	3.7	56.2	100.0
Romania	RO312	8	15	13	4	51	91	8.8	16.5	14.3	4.4	56.0	100.0
Romania	RO313	17	22	15	5	74	133	12.8	16.5	11.3	3.8	55.6	100.0
Romania	RO314	10	15	11	3	48	87	11.5	17.2	12.6	3.4	55.2	100.0
Romania	RO315	7	13	12	3	44	79	8.9	16.5	15.2	3.8	55.7	100.0
Romania	RO316	25	36	26	8	122	217	11.5	16.6	12.0	3.7	56.2	100.0
Romania	RO317	14	26	16	5	73	134	10.4	19.4	11.9	3.7	54.5	100.0
Romania	RO321	62	81	62	18	266	489	12.7	16.6	12.7	3.7	54.4	100.0
Romania	RO322	11	15	11	3	47	87	12.6	17.2	12.6	3.4	54.0	100.0
Romania	RO411	23	41	20	7	108	199	11.6	20.6	10.1	3.5	54.3	100.0
Romania	RO412	13	17	8	3	48	89	14.6	19.1	9.0	3.4	53.9	100.0
Romania	RO413	9	18	8	3	44	82	11.0	22.0	9.8	3.7	53.7	100.0
Romania	RO414	15	24	14	5	71	129	11.6	18.6	10.9	3.9	55.0	100.0
Romania	RO415	11	15	9	3	47	85	12.9	17.6	10.6	3.5	55.3	100.0
Romania	RO421	11	35	10	5	70	131	8.4	26.7	7.6	3.8	53.4	100.0
Romania	RO422	8	23	8	3	50	92	8.7	25.0	8.7	3.3	54.3	100.0
Romania	RO423	14	26	11	4	71	126	11.1	20.6	8.7	3.2	56.3	100.0

Romania	RO424	13	45	12	6	88	164	7.9	27.4	7.3	3.7	53.7	100.0
Serbia	RS110	51	131	36	18	242	478	10.7	27.4	7.5	3.8	50.6	100.0
Serbia	RS121	4	21	4	3	33	65	6.2	32.3	6.2	4.6	50.8	100.0
Serbia	RS122	8	26	7	3	47	91	8.8	28.6	7.7	3.3	51.6	100.0
Serbia	RS123	15	50	12	7	86	170	8.8	29.4	7.1	4.1	50.6	100.0
Serbia	RS124	3	16	3	2	27	51	5.9	31.4	5.9	3.9	52.9	100.0
Serbia	RS125	4	20	4	2	31	61	6.6	32.8	6.6	3.3	50.8	100.0
Serbia	RS126	5	18	4	2	31	60	8.3	30.0	6.7	3.3	51.7	100.0
Serbia	RS127	10	27	7	4	48	96	10.4	28.1	7.3	4.2	50.0	100.0
Serbia	RS211	5	22	7	4	48	86	5.8	25.6	8.1	4.7	55.8	100.0
Serbia	RS212	5	16	5	3	31	60	8.3	26.7	8.3	5.0	51.7	100.0
Serbia	RS213	7	27	9	4	50	97	7.2	27.8	9.3	4.1	51.5	100.0
Serbia	RS214	6	18	5	3	37	69	8.7	26.1	7.2	4.3	53.6	100.0
Serbia	RS215	7	21	6	3	41	78	9.0	26.9	7.7	3.8	52.6	100.0
Serbia	RS216	7	22	7	3	45	84	8.3	26.2	8.3	3.6	53.6	100.0
Serbia	RS217	5	21	7	3	45	81	6.2	25.9	8.6	3.7	55.6	100.0
Serbia	RS218	8	23	7	3	45	86	9.3	26.7	8.1	3.5	52.3	100.0
Serbia	RS221	2	14	4	2	25	47	4.3	29.8	8.5	4.3	53.2	100.0
Serbia	RS222	6	19	6	3	36	70	8.6	27.1	8.6	4.3	51.4	100.0
Serbia	RS223	3	14	4	2	27	50	6.0	28.0	8.0	4.0	54.0	100.0
Serbia	RS224	5	19	7	3	40	74	6.8	25.7	9.5	4.1	54.1	100.0
Serbia	RS225	9	31	11	5	65	121	7.4	25.6	9.1	4.1	53.7	100.0
Serbia	RS226	1	9	3	1	20	34	2.9	26.5	8.8	2.9	58.8	100.0
Serbia	RS227	6	16	5	2	31	60	10.0	26.7	8.3	3.3	51.7	100.0
Serbia	RS228	3	15	6	2	33	59	5.1	25.4	10.2	3.4	55.9	100.0
Serbia	RS229	2	9	3	1	19	34	5.9	26.5	8.8	2.9	55.9	100.0
Sweden	SE110	14	48	9	24	189	284	4.9	16.9	3.2	8.5	66.5	100.0
Sweden	SE121	2	7	1	4	32	46	4.3	15.2	2.2	8.7	69.6	100.0
Sweden	SE122	2	8	1	4	34	49	4.1	16.3	2.0	8.2	69.4	100.0
Sweden	SE123	3	12	2	6	49	72	4.2	16.7	2.8	8.3	68.1	100.0
Sweden	SE124	2	8	1	4	33	48	4.2	16.7	2.1	8.3	68.8	100.0
Sweden	SE125	2	7	1	3	30	43	4.7	16.3	2.3	7.0	69.8	100.0
Sweden	SE211	2	11	2	5	39	59	3.4	18.6	3.4	8.5	66.1	100.0
Sweden	SE212	1	7	1	3	21	33	3.0	21.2	3.0	9.1	63.6	100.0
Sweden	SE213	2	10	2	5	32	51	3.9	19.6	3.9	9.8	62.7	100.0
Sweden	SE214	0	2	0	1	8	11	0.0	18.2	0.0	9.1	72.7	100.0
Sweden	SE221	1	8	1	3	21	34	2.9	23.5	2.9	8.8	61.8	100.0
Sweden	SE224	5	55	6	20	140	226	2.2	24.3	2.7	8.8	61.9	100.0
Sweden	SE231	1	12	1	5	35	54	1.9	22.2	1.9	9.3	64.8	100.0
Sweden	SE232	9	51	6	23	179	268	3.4	19.0	2.2	8.6	66.8	100.0

Sweden	SE311	2	9	1	4	38	54	3.7	16.7	1.9	7.4	70.4	100.0
Sweden	SE312	2	7	1	3	35	48	4.2	14.6	2.1	6.3	72.9	100.0
Sweden	SE313	2	7	1	4	37	51	3.9	13.7	2.0	7.8	72.5	100.0
Sweden	SE321	2	6	1	3	33	45	4.4	13.3	2.2	6.7	73.3	100.0
Sweden	SE322	1	3	1	1	18	24	4.2	12.5	4.2	4.2	75.0	100.0
Sweden	SE331	1	4	1	3	29	38	2.6	10.5	2.6	7.9	76.3	100.0
Sweden	SE332	2	4	1	2	32	41	4.9	9.8	2.4	4.9	78.0	100.0
Slovenia	SI	24	163	16	25	226	454	5.3	35.9	3.5	5.5	49.8	100.0
Slovakia	SK010	6	50	5	5	65	131	4.6	38.2	3.8	3.8	49.6	100.0
Slovakia	SK021	6	46	5	5	63	125	4.8	36.8	4.0	4.0	50.4	100.0
Slovakia	SK022	6	47	6	5	67	131	4.6	35.9	4.6	3.8	51.1	100.0
Slovakia	SK023	8	62	8	7	88	173	4.6	35.8	4.6	4.0	50.9	100.0
Slovakia	SK031	6	51	7	6	78	148	4.1	34.5	4.7	4.1	52.7	100.0
Slovakia	SK032	7	53	8	6	82	156	4.5	34.0	5.1	3.8	52.6	100.0
Slovakia	SK041	6	49	10	6	79	150	4.0	32.7	6.7	4.0	52.7	100.0
Slovakia	SK042	6	51	11	6	83	157	3.8	32.5	7.0	3.8	52.9	100.0
United Kingdom	UKC1	18	25	2	16	160	221	8.1	11.3	0.9	7.2	72.4	100.0
United Kingdom	UKC2	22	29	3	20	198	272	8.1	10.7	1.1	7.4	72.8	100.0
United Kingdom	UKD1	7	11	1	7	75	101	6.9	10.9	1.0	6.9	74.3	100.0
United Kingdom	UKD3	41	50	5	29	316	441	9.3	11.3	1.1	6.6	71.7	100.0
United Kingdom	UKD4	22	30	3	18	195	268	8.2	11.2	1.1	6.7	72.8	100.0
United Kingdom	UKD6	14	18	2	10	114	158	8.9	11.4	1.3	6.3	72.2	100.0
United Kingdom	UKD7	22	31	3	19	206	281	7.8	11.0	1.1	6.8	73.3	100.0
United Kingdom	UKE1	17	21	2	13	118	171	9.9	12.3	1.2	7.6	69.0	100.0
United Kingdom	UKE2	14	17	2	10	103	146	9.6	11.6	1.4	6.8	70.5	100.0
United Kingdom	UKE3	25	26	2	15	163	231	10.8	11.3	0.9	6.5	70.6	100.0
United Kingdom	UKE4	34	39	4	23	253	353	9.6	11.0	1.1	6.5	71.7	100.0
United Kingdom	UKF1	41	42	4	24	255	366	11.2	11.5	1.1	6.6	69.7	100.0
United Kingdom	UKF2	32	33	3	18	185	271	11.8	12.2	1.1	6.6	68.3	100.0
United Kingdom	UKF3	17	18	2	10	99	146	11.6	12.3	1.4	6.8	67.8	100.0
United Kingdom	UKG1	25	27	2	16	166	236	10.6	11.4	0.8	6.8	70.3	100.0
United Kingdom	UKG2	28	31	3	18	198	278	10.1	11.2	1.1	6.5	71.2	100.0
United Kingdom	UKG3	46	51	4	29	310	440	10.5	11.6	0.9	6.6	70.5	100.0
United Kingdom	UKH1	56	68	5	34	301	464	12.1	14.7	1.1	7.3	64.9	100.0
United Kingdom	UKH2	33	36	3	18	178	268	12.3	13.4	1.1	6.7	66.4	100.0
United Kingdom	UKH3	41	48	3	24	217	333	12.3	14.4	0.9	7.2	65.2	100.0
United Kingdom	UKI3	13	15	1	8	71	108	12.0	13.9	0.9	7.4	65.7	100.0
United Kingdom	UKI4	24	27	2	14	130	197	12.2	13.7	1.0	7.1	66.0	100.0
United Kingdom	UKI5	29	34	2	17	157	239	12.1	14.2	0.8	7.1	65.7	100.0
United Kingdom	UKI6	20	23	2	12	110	167	12.0	13.8	1.2	7.2	65.9	100.0

United Kingdom	UKI7	28	32	2	16	152	230	12.2	13.9	0.9	7.0	66.1	100.0
United Kingdom	UKJ1	37	42	3	23	217	322	11.5	13.0	0.9	7.1	67.4	100.0
United Kingdom	UKJ2	57	76	5	42	347	527	10.8	14.4	0.9	8.0	65.8	100.0
United Kingdom	UKJ3	32	43	3	27	224	329	9.7	13.1	0.9	8.2	68.1	100.0
United Kingdom	UKJ4	35	48	3	26	209	321	10.9	15.0	0.9	8.1	65.1	100.0
United Kingdom	UKK1	39	46	3	29	280	397	9.8	11.6	0.8	7.3	70.5	100.0
United Kingdom	UKK2	22	34	2	23	193	274	8.0	12.4	0.7	8.4	70.4	100.0
United Kingdom	UKK3	5	12	1	11	90	119	4.2	10.1	0.8	9.2	75.6	100.0
United Kingdom	UKK4	15	27	2	21	178	243	6.2	11.1	0.8	8.6	73.3	100.0
United Kingdom	UKL1	28	42	3	30	297	400	7.0	10.5	0.8	7.5	74.3	100.0
United Kingdom	UKL2	16	21	2	14	143	196	8.2	10.7	1.0	7.1	73.0	100.0
United Kingdom	UKM5	5	7	1	6	61	80	6.3	8.8	1.3	7.5	76.3	100.0
United Kingdom	UKM6	5	7	1	6	74	93	5.4	7.5	1.1	6.5	79.6	100.0
United Kingdom	UKM7	24	31	3	23	264	345	7.0	9.0	0.9	6.7	76.5	100.0
United Kingdom	UKM8	18	24	3	17	218	280	6.4	8.6	1.1	6.1	77.9	100.0
United Kingdom	UKM9	13	18	2	12	148	193	6.7	9.3	1.0	6.2	76.7	100.0
United Kingdom	UKN0	15	24	2	15	210	266	5.6	9.0	0.8	5.6	78.9	100.0

Table S6. Average annual O_3 attributable mortality rate according to O_3 emission sources in European regions, 2015-2017

Country	NUTS ID	Population	Number of deaths per million population					
			National	34 European countries	Other countries	Ocean and sea	Outside study domain	Overall (95% eCI)
Albania	AL011	126,667	2.63	15.79	5.26	2.63	34.21	60.53 (42.11 to 81.58)
Albania	AL012	283,161	3.53	14.13	4.71	4.71	28.25	55.33 (37.67 to 74.16)
Albania	AL013	80,169	0.00	12.47	8.32	4.16	29.11	54.05 (37.42 to 74.84)
Albania	AL014	129,477	2.57	15.45	5.15	5.15	33.47	61.79 (41.19 to 82.38)
Albania	AL015	209,059	3.19	19.13	6.38	4.78	38.27	71.75 (47.83 to 95.67)
Albania	AL021	285,161	3.51	15.20	4.68	3.51	32.73	59.62 (40.91 to 80.66)
Albania	AL022	855,872	3.51	12.46	3.89	3.89	26.09	49.85 (33.49 to 66.6)
Albania	AL031	133,295	5.00	17.51	5.00	5.00	37.51	70.02 (47.51 to 95.03)
Albania	AL032	303,443	3.30	17.58	5.49	6.59	34.05	67.01 (43.94 to 86.78)
Albania	AL033	66,617	5.00	25.02	5.00	10.01	55.04	100.07 (65.05 to 130.1)
Albania	AL034	215,530	3.09	20.11	7.73	4.64	47.94	83.52 (55.68 to 111.35)
Albania	AL035	188,627	3.53	19.44	5.30	7.07	37.11	72.45 (49.48 to 98.96)
Austria	AT111	37,682	8.85	35.38	0.00	0.00	44.23	88.46 (61.92 to 123.84)
Austria	AT112	156,005	6.41	27.78	2.14	2.14	38.46	76.92 (53.42 to 104.7)
Austria	AT113	97,309	6.85	30.83	3.43	3.43	41.11	85.64 (58.23 to 113.04)
Austria	AT121	244,710	6.81	23.16	1.36	2.72	34.05	68.11 (46.31 to 92.63)
Austria	AT122	258,284	6.45	27.10	2.58	2.58	40.01	78.72 (54.2 to 107.12)
Austria	AT123	153,041	6.53	23.96	2.18	2.18	34.85	69.7 (45.74 to 91.48)
Austria	AT124	218,756	6.10	28.95	1.52	3.05	42.67	82.28 (54.86 to 109.71)
Austria	AT125	124,414	5.36	32.15	2.68	2.68	45.55	88.41 (58.94 to 117.89)
Austria	AT126	321,920	6.21	22.78	2.07	2.07	32.10	65.23 (43.49 to 86.98)
Austria	AT127	335,598	6.95	23.84	1.99	2.98	34.76	70.52 (46.68 to 93.37)
Austria	AT130	1,848,480	6.13	21.28	1.98	2.34	31.02	62.75 (42.2 to 83.67)
Austria	AT211	284,360	4.69	26.96	2.34	3.52	41.03	78.54 (52.75 to 105.5)
Austria	AT212	125,044	2.67	29.32	2.67	5.33	45.32	85.3 (55.98 to 111.96)
Austria	AT213	150,621	4.43	26.56	2.21	4.43	42.05	79.67 (55.33 to 108.44)
Austria	AT221	431,149	5.41	20.87	2.32	2.32	32.47	63.4 (42.52 to 84.27)
Austria	AT222	79,882	4.17	25.04	0.00	4.17	41.73	75.11 (54.25 to 104.32)
Austria	AT223	161,085	6.21	31.04	2.07	4.14	49.66	93.12 (62.08 to 124.16)
Austria	AT224	265,901	5.01	23.82	2.51	2.51	36.35	70.2 (47.64 to 95.27)
Austria	AT225	193,668	6.88	27.54	1.72	3.44	39.59	79.17 (53.36 to 106.71)
Austria	AT226	101,089	6.59	29.68	3.30	3.30	49.46	92.33 (59.35 to 118.71)
Austria	AT311	282,719	4.72	22.40	1.18	2.36	33.01	63.67 (43.62 to 86.07)
Austria	AT312	578,010	6.92	21.34	1.73	2.31	32.87	65.17 (43.25 to 85.93)
Austria	AT313	207,265	6.43	20.91	1.61	1.61	32.16	62.72 (41.81 to 83.63)
Austria	AT314	154,421	6.48	23.74	2.16	2.16	34.54	69.08 (45.33 to 92.82)

Austria	AT315	235,041	5.67	22.69	1.42	2.84	35.45	68.07 (45.38 to 89.35)
Austria	AT321	20,444	0.00	16.30	0.00	0.00	48.91	65.22 (48.91 to 97.83)
Austria	AT322	166,162	2.01	20.06	2.01	2.01	36.11	62.19 (42.13 to 84.26)
Austria	AT323	359,953	3.70	21.30	0.93	1.85	30.56	58.34 (38.89 to 77.79)
Austria	AT331	32,165	0.00	20.73	0.00	0.00	31.09	51.82 (41.45 to 82.91)
Austria	AT332	305,705	2.18	18.54	1.09	2.18	35.98	59.97 (40.34 to 80.69)
Austria	AT333	48,909	0.00	20.45	0.00	0.00	40.89	61.34 (47.71 to 95.42)
Austria	AT334	102,776	3.24	19.46	0.00	3.24	35.68	61.62 (38.92 to 81.08)
Austria	AT335	251,761	2.65	19.86	1.32	2.65	33.10	59.58 (39.72 to 79.44)
Austria	AT341	90,188	3.70	22.18	0.00	3.70	33.26	62.83 (44.35 to 85.01)
Austria	AT342	295,620	1.13	21.42	1.13	2.26	28.19	54.12 (36.08 to 72.16)
Belgium	BE10	1,197,493	3.34	15.03	0.56	3.62	30.34	52.89 (35.35 to 70.42)
Belgium	BE21	1,833,587	3.82	17.82	0.73	4.00	35.81	62.17 (41.45 to 82.53)
Belgium	BE22	867,688	3.84	18.06	0.77	3.46	32.27	58.39 (39.18 to 77.6)
Belgium	BE23	1,493,212	3.35	16.74	0.67	4.24	36.39	61.39 (41.07 to 81.7)
Belgium	BE24	1,126,595	4.14	17.75	0.59	4.14	35.51	62.13 (41.42 to 82.25)
Belgium	BE25	1,186,509	2.53	18.82	0.84	5.62	41.86	69.67 (46.64 to 92.71)
Belgium	BE31	398,333	3.35	16.74	0.84	4.18	33.47	58.58 (39.33 to 78.66)
Belgium	BE32	1,341,214	3.48	20.88	0.75	4.97	42.00	72.07 (48.22 to 95.93)
Belgium	BE33	1,104,201	4.53	23.24	0.91	4.23	40.45	73.36 (48.9 to 97.51)
Belgium	BE34	283,286	2.35	21.18	1.18	3.53	36.48	64.72 (43.54 to 87.07)
Belgium	BE35	492,559	3.38	20.98	0.68	4.06	39.25	68.35 (46.02 to 91.36)
Bulgaria	BG311	90,098	7.40	44.40	14.80	7.40	85.09	159.09 (103.59 to 210.88)
Bulgaria	BG312	135,855	9.81	39.26	14.72	4.91	80.97	149.67 (98.14 to 196.29)
Bulgaria	BG313	170,387	9.78	33.26	13.69	5.87	74.34	136.94 (91.95 to 181.94)
Bulgaria	BG314	250,021	9.33	30.66	13.33	5.33	70.66	129.32 (86.66 to 171.99)
Bulgaria	BG315	130,297	10.23	33.26	15.35	5.12	79.31	143.26 (97.21 to 191.87)
Bulgaria	BG321	243,545	8.21	26.00	13.69	5.47	64.33	117.71 (79.38 to 157.4)
Bulgaria	BG322	113,303	11.77	29.42	14.71	5.88	79.43	141.21 (94.14 to 188.29)
Bulgaria	BG323	224,546	7.42	26.72	14.84	4.45	63.83	117.27 (77.19 to 154.39)
Bulgaria	BG324	116,351	5.73	22.92	14.32	5.73	63.03	111.73 (74.49 to 151.84)
Bulgaria	BG325	112,609	5.92	23.68	17.76	5.92	62.16	115.44 (76.96 to 150.96)
Bulgaria	BG331	472,876	4.23	16.21	15.51	6.34	52.87	95.16 (62.74 to 124.77)
Bulgaria	BG332	179,498	3.71	20.43	18.57	7.43	65.00	115.14 (78 to 154.13)
Bulgaria	BG333	175,022	5.71	20.95	15.24	5.71	60.94	108.56 (72.37 to 144.74)
Bulgaria	BG334	114,450	5.82	23.30	14.56	5.82	64.07	113.59 (75.72 to 151.45)
Bulgaria	BG341	413,083	4.84	16.95	15.33	6.46	55.68	99.25 (66.17 to 132.34)
Bulgaria	BG342	190,513	8.75	21.00	15.75	5.25	62.99	113.73 (75.24 to 150.47)
Bulgaria	BG343	123,073	8.13	21.67	18.96	8.13	73.13	130 (86.67 to 170.63)
Bulgaria	BG344	322,523	10.34	23.77	14.47	5.17	66.15	119.89 (80.61 to 159.16)

Bulgaria	BG411	1,321,357	7.57	19.17	8.07	3.28	49.70	87.79 (58.78 to 116.8)
Bulgaria	BG412	235,222	9.92	29.76	12.75	5.67	75.11	133.21 (89.28 to 177.14)
Bulgaria	BG413	311,653	5.35	20.32	9.63	4.28	59.90	99.47 (66.31 to 132.63)
Bulgaria	BG414	124,674	10.69	32.08	13.37	5.35	80.21	141.7 (93.58 to 187.15)
Bulgaria	BG415	124,628	10.70	34.77	13.37	5.35	90.94	155.13 (101.64 to 203.27)
Bulgaria	BG421	672,560	11.89	20.82	11.40	4.46	58.98	107.55 (71.86 to 142.74)
Bulgaria	BG422	234,685	11.36	22.73	14.20	5.68	68.18	122.15 (82.38 to 163.34)
Bulgaria	BG423	262,240	11.44	22.88	11.44	5.08	64.83	115.67 (77.54 to 153.8)
Bulgaria	BG424	110,573	9.04	24.12	12.06	6.03	69.34	120.58 (81.39 to 159.77)
Bulgaria	BG425	151,331	8.81	17.62	11.01	4.41	52.86	94.72 (63.88 to 127.76)
Switzerland	CH011	778,201	3.86	15.85	0.86	2.57	29.13	52.26 (35.12 to 69.82)
Switzerland	CH012	337,025	2.97	16.81	0.99	2.97	40.55	64.29 (42.53 to 85.06)
Switzerland	CH013	486,724	2.74	16.44	0.68	2.74	28.76	51.36 (34.24 to 67.8)
Switzerland	CH021	1,021,135	5.88	19.59	0.98	2.94	36.56	65.94 (44.4 to 88.14)
Switzerland	CH022	309,457	4.31	14.00	1.08	2.15	25.85	47.39 (31.24 to 61.4)
Switzerland	CH023	267,753	4.98	19.92	1.24	2.49	33.61	62.25 (41.08 to 83.41)
Switzerland	CH024	177,991	3.75	20.60	1.87	3.75	37.46	67.42 (44.95 to 88.02)
Switzerland	CH025	72,901	4.57	22.86	0.00	4.57	36.58	68.59 (41.15 to 86.88)
Switzerland	CH031	192,344	3.47	29.46	1.73	3.47	43.33	81.45 (55.46 to 109.18)
Switzerland	CH032	284,295	3.52	21.10	1.17	2.34	32.83	60.97 (41.04 to 82.07)
Switzerland	CH033	658,351	5.06	16.20	0.51	2.53	26.83	51.14 (34.43 to 68.35)
Switzerland	CH040	1,476,273	5.87	16.26	0.68	2.26	26.87	51.93 (35 to 69.32)
Switzerland	CH051	40,080	8.32	24.95	0.00	0.00	41.58	74.85 (49.9 to 99.8)
Switzerland	CH052	80,343	4.15	20.74	0.00	4.15	29.04	58.08 (37.34 to 78.83)
Switzerland	CH053	54,685	6.10	18.29	0.00	0.00	36.57	60.96 (42.67 to 85.34)
Switzerland	CH054	15,984	0.00	20.85	0.00	0.00	20.85	41.71 (41.71 to 83.42)
Switzerland	CH055	500,532	5.99	17.98	0.67	2.66	29.97	57.27 (38.63 to 75.92)
Switzerland	CH056	196,984	3.38	22.00	1.69	3.38	40.61	71.07 (47.38 to 93.07)
Switzerland	CH057	268,918	4.96	16.11	1.24	2.48	26.03	50.82 (33.47 to 66.93)
Switzerland	CH061	400,817	5.82	15.80	0.83	2.49	28.28	53.22 (35.76 to 70.69)
Switzerland	CH062	36,106	9.23	27.70	0.00	0.00	46.16	83.09 (55.39 to 110.78)
Switzerland	CH063	155,004	4.30	17.20	0.00	2.15	27.96	51.61 (36.56 to 70.97)
Switzerland	CH064	37,216	8.96	17.91	0.00	0.00	26.87	53.74 (35.83 to 71.65)
Switzerland	CH065	42,506	7.84	15.68	0.00	0.00	31.37	54.89 (39.21 to 78.42)
Switzerland	CH066	122,898	5.42	13.56	0.00	2.71	24.41	46.11 (29.84 to 59.67)
Switzerland	CH070	352,598	2.84	31.20	1.89	3.78	37.81	77.52 (51.05 to 102.1)
Cyprus	CY000	853,591	1.17	6.64	10.93	8.20	31.63	58.58 (39.44 to 78.1)
Czechia	CZ010	1,275,387	6.01	23.26	1.57	2.87	36.33	70.04 (47.04 to 93.31)
Czechia	CZ020	1,333,488	5.75	23.25	1.75	3.00	36.00	69.74 (46.49 to 92.24)
Czechia	CZ031	638,528	3.65	26.62	2.09	3.13	39.67	75.17 (50.12 to 99.19)

Czechia	CZ032	577,796	4.04	26.54	1.73	2.88	40.38	75.57 (50.77 to 100.38)
Czechia	CZ041	297,389	3.36	26.90	1.12	3.36	42.59	77.34 (51.56 to 103.12)
Czechia	CZ042	822,314	5.68	26.75	1.62	3.65	40.94	78.64 (52.7 to 104.58)
Czechia	CZ051	440,107	5.30	25.75	1.51	3.03	38.63	74.22 (49.99 to 99.22)
Czechia	CZ052	551,226	5.44	25.40	2.42	3.02	38.10	74.38 (49.59 to 99.17)
Czechia	CZ053	516,986	5.80	24.50	2.58	3.22	36.75	72.86 (48.36 to 96.07)
Czechia	CZ063	509,310	5.24	24.87	1.96	3.27	37.31	72.65 (48.43 to 96.21)
Czechia	CZ064	1,177,474	5.38	25.76	2.55	2.83	37.65	74.17 (49.54 to 98.52)
Czechia	CZ071	634,383	5.78	26.80	2.63	3.15	39.93	78.29 (52.54 to 104.04)
Czechia	CZ072	584,173	5.14	26.82	2.85	2.85	39.37	77.03 (51.93 to 102.71)
Czechia	CZ080	1,211,688	4.68	28.61	3.30	3.30	41.26	81.15 (54.19 to 107.84)
Germany	DE11	4,075,697	12.19	16.85	0.98	3.03	35.74	68.78 (45.96 to 91.27)
Germany	DE12	2,764,585	11.94	18.33	0.96	3.26	37.02	71.5 (47.87 to 95.13)
Germany	DE13	2,227,641	8.08	20.65	0.90	3.29	37.11	70.03 (46.84 to 93.07)
Germany	DE14	1,824,972	9.50	18.63	1.10	2.92	35.80	67.95 (45.48 to 90.23)
Germany	DE21	4,597,945	10.95	16.82	1.09	2.54	33.42	64.81 (43.35 to 86.2)
Germany	DE22	1,214,778	12.62	20.31	1.37	3.02	39.51	76.83 (51.31 to 102.08)
Germany	DE23	1,094,471	12.79	18.58	1.52	3.05	40.20	76.14 (50.86 to 101.11)
Germany	DE24	1,061,137	14.45	20.10	1.57	3.77	45.55	85.44 (57.17 to 114.03)
Germany	DE25	1,740,896	13.98	18.19	1.34	3.26	40.21	76.97 (51.51 to 102.25)
Germany	DE26	1,306,870	13.77	17.60	1.02	3.32	39.28	74.99 (50.25 to 99.98)
Germany	DE27	1,849,663	10.99	18.92	1.08	2.88	36.94	70.82 (47.4 to 94.07)
Germany	DE30	3,544,551	10.34	15.23	1.41	4.04	36.30	67.33 (45.05 to 89.53)
Germany	DE40	2,485,347	12.74	19.31	1.88	4.96	45.47	84.36 (56.33 to 112.12)
Germany	DE50	673,291	8.91	15.35	0.99	5.45	41.09	71.79 (48.02 to 96.05)
Germany	DE60	1,797,805	7.79	12.42	1.11	4.64	35.78	61.74 (41.35 to 82.14)
Germany	DE71	3,928,868	12.39	16.20	0.93	3.22	36.14	68.89 (46.07 to 91.54)
Germany	DE72	1,039,640	13.15	17.63	0.96	3.85	40.40	75.99 (50.98 to 101.32)
Germany	DE73	1,213,095	14.29	18.96	1.10	4.12	44.24	82.71 (55.51 to 110.19)
Germany	DE80	1,608,323	9.12	18.03	1.87	6.42	49.53	84.97 (57 to 113.37)
Germany	DE91	1,592,315	13.61	18.00	1.26	4.61	44.17	81.64 (54.64 to 108.65)
Germany	DE92	2,131,200	12.36	17.36	1.25	5.01	43.79	79.77 (53.33 to 105.89)
Germany	DE93	1,697,023	9.82	15.32	1.18	5.50	42.23	74.05 (49.5 to 98.6)
Germany	DE94	2,494,911	7.88	15.63	1.07	5.21	39.55	69.34 (46.23 to 92.05)
Germany	DEA1	5,167,881	10.19	20.19	0.97	4.45	43.67	79.47 (53.15 to 105.65)
Germany	DEA2	4,419,435	9.28	17.80	0.83	3.77	38.32	69.99 (46.84 to 93.15)
Germany	DEA3	2,608,856	9.84	17.76	0.89	4.34	40.12	72.96 (48.81 to 96.98)
Germany	DEA4	2,049,048	10.90	16.27	0.98	4.23	39.04	71.42 (47.83 to 95.17)
Germany	DEA5	3,581,243	12.57	20.29	1.02	4.75	46.26	84.89 (56.78 to 112.9)
Germany	DEB1	1,487,688	12.55	20.39	1.12	4.26	44.59	82.9 (55.57 to 110.24)

Germany	DEB2	528,226	8.83	20.82	1.26	3.79	42.28	76.99 (51.75 to 102.86)
Germany	DEB3	2,035,116	12.28	19.33	0.98	3.60	40.13	76.33 (50.94 to 101.22)
Germany	DEC0	993,868	11.40	25.83	1.34	4.70	50.64	93.91 (62.72 to 124.43)
Germany	DED2	1,598,562	12.72	22.52	1.88	4.17	44.83	86.12 (57.55 to 114.27)
Germany	DED4	1,457,013	15.79	25.62	2.06	4.80	54.45	102.72 (68.63 to 136.35)
Germany	DED5	1,020,230	14.38	19.28	1.63	4.25	44.43	83.97 (56.2 to 111.41)
Germany	DEE0	2,235,088	16.41	21.62	1.79	5.37	52.65	97.83 (65.32 to 129.9)
Germany	DEF0	2,865,331	8.26	15.36	1.51	6.17	45.02	76.31 (51.07 to 101.56)
Germany	DEG0	2,159,202	15.44	21.00	1.54	4.48	48.47	90.93 (60.82 to 120.88)
Denmark	DK011	758,198	1.32	11.43	1.32	4.40	29.46	47.92 (31.65 to 63.31)
Denmark	DK012	540,814	1.85	14.79	1.85	5.55	39.45	63.48 (42.53 to 85.06)
Denmark	DK013	458,016	2.18	14.56	1.46	5.82	40.03	64.04 (42.94 to 85.15)
Denmark	DK014	39,814	0.00	25.12	0.00	8.37	58.61	92.09 (66.98 to 133.96)
Denmark	DK021	245,234	1.36	13.59	1.36	5.44	35.34	57.09 (38.06 to 76.12)
Denmark	DK022	583,655	2.28	19.42	2.28	6.85	48.54	79.38 (53.11 to 105.08)
Denmark	DK031	492,586	1.35	16.92	1.35	6.09	43.31	69.02 (46.02 to 92.03)
Denmark	DK032	721,285	1.39	15.71	1.39	6.01	40.67	65.16 (43.44 to 85.96)
Denmark	DK041	428,150	1.56	13.24	1.56	5.45	38.93	60.73 (40.48 to 80.97)
Denmark	DK042	870,327	1.53	11.87	1.15	4.98	34.09	53.62 (36 to 71.62)
Denmark	DK050	586,154	1.14	13.08	1.14	6.26	42.08	63.69 (42.65 to 84.73)
Estonia	EE001	580,732	1.15	12.63	2.87	5.17	39.03	60.84 (40.75 to 80.93)
Estonia	EE004	150,120	2.22	17.76	4.44	6.66	48.85	79.94 (53.29 to 104.36)
Estonia	EE008	316,948	2.10	13.67	4.21	5.26	45.22	70.46 (47.33 to 94.65)
Estonia	EE009	124,272	2.68	16.09	5.36	5.36	50.96	80.47 (53.65 to 104.61)
Estonia	EE00A	144,324	2.31	18.48	6.93	6.93	57.74	92.38 (62.36 to 122.41)
Greece	EL301	589,551	6.22	14.14	10.18	7.35	44.67	82.55 (55.97 to 110.82)
Greece	EL302	483,511	6.20	14.48	10.34	7.58	44.81	83.42 (55.84 to 110.99)
Greece	EL303	936,487	8.90	19.58	14.24	11.03	60.51	114.26 (76.53 to 151.63)
Greece	EL304	530,847	6.91	15.70	11.30	9.42	47.09	90.42 (60.28 to 120.56)
Greece	EL305	557,026	4.79	11.37	7.78	5.98	34.11	64.03 (43.09 to 84.98)
Greece	EL306	177,597	5.63	11.26	7.51	5.63	37.54	67.57 (45.05 to 90.09)
Greece	EL307	508,515	9.18	17.04	12.45	9.83	54.41	102.91 (68.83 to 137)
Greece	EL411	104,541	3.19	15.94	19.13	12.75	57.39	108.41 (73.34 to 143.48)
Greece	EL412	43,540	7.66	15.31	22.97	15.31	61.25	122.49 (84.21 to 168.43)
Greece	EL413	54,216	6.15	12.30	18.44	12.30	49.19	98.37 (67.63 to 135.26)
Greece	EL421	210,211	3.17	7.93	17.44	14.27	34.89	77.7 (50.74 to 101.49)
Greece	EL422	127,017	5.25	13.12	13.12	15.75	39.36	86.6 (57.74 to 115.47)
Greece	EL431	311,509	4.28	12.84	11.77	11.77	40.66	81.32 (54.57 to 109.15)
Greece	EL432	74,590	4.47	17.88	17.88	17.88	53.63	111.72 (75.97 to 147.47)
Greece	EL433	87,351	3.82	11.45	11.45	11.45	38.16	76.32 (49.61 to 99.22)

Greece	EL434	158,928	4.19	16.78	12.58	12.58	46.14	92.28 (62.92 to 123.75)
Greece	EL511	147,727	2.26	20.31	15.79	6.77	56.41	101.54 (67.69 to 135.38)
Greece	EL512	112,201	2.97	14.85	8.91	2.97	41.59	71.3 (47.53 to 98.04)
Greece	EL513	111,834	2.98	20.86	11.92	5.96	50.67	92.4 (62.59 to 125.19)
Greece	EL514	97,026	6.87	20.61	10.31	6.87	61.84	106.5 (72.15 to 140.86)
Greece	EL515	134,954	4.94	22.23	12.35	7.41	59.28	106.21 (69.16 to 135.85)
Greece	EL521	141,605	9.42	21.19	9.42	4.71	54.14	98.87 (65.91 to 129.47)
Greece	EL522	1,110,203	7.21	15.91	8.11	4.20	43.24	78.66 (52.54 to 104.19)
Greece	EL523	80,532	8.28	24.83	12.42	4.14	66.23	115.9 (78.64 to 157.29)
Greece	EL524	138,845	9.60	21.61	9.60	4.80	55.22	100.83 (67.22 to 136.84)
Greece	EL525	131,347	7.61	20.30	10.15	5.08	50.76	93.9 (60.91 to 121.81)
Greece	EL526	170,331	7.83	25.44	11.74	5.87	72.41	123.29 (84.15 to 166.34)
Greece	EL527	110,472	6.03	18.10	9.05	6.03	48.28	87.5 (60.35 to 117.68)
Greece	EL531	174,426	7.64	21.02	9.56	5.73	55.42	99.37 (64.98 to 129.95)
Greece	EL532	47,982	6.95	20.84	6.95	6.95	55.58	97.26 (62.52 to 131.99)
Greece	EL533	50,337	6.62	19.87	13.24	6.62	59.60	105.95 (66.22 to 132.44)
Greece	EL541	123,195	8.12	24.35	8.12	10.82	59.53	110.94 (75.76 to 148.82)
Greece	EL542	45,134	7.39	22.16	7.39	14.77	51.70	103.4 (66.47 to 132.94)
Greece	EL543	168,062	3.97	19.83	7.93	5.95	51.57	89.25 (61.49 to 122.97)
Greece	EL611	239,425	9.75	22.28	9.75	6.96	59.87	108.59 (72.4 to 144.79)
Greece	EL612	283,357	7.06	16.47	8.23	4.71	47.05	83.52 (56.47 to 111.76)
Greece	EL613	204,979	6.50	17.89	9.76	6.50	50.41	91.07 (60.17 to 120.34)
Greece	EL621	40,024	8.33	24.99	8.33	24.99	49.97	116.6 (74.96 to 149.91)
Greece	EL622	102,840	6.48	32.41	9.72	16.21	58.34	123.17 (81.03 to 162.06)
Greece	EL623	38,985	8.55	25.65	8.55	17.10	51.30	111.15 (76.95 to 153.91)
Greece	EL624	23,949	0.00	27.84	13.92	13.92	69.59	125.27 (97.43 to 180.94)
Greece	EL631	205,006	6.50	22.76	8.13	11.38	56.91	105.69 (69.92 to 139.83)
Greece	EL632	304,241	4.38	19.72	7.67	9.86	50.40	92.03 (62.45 to 124.9)
Greece	EL633	156,994	4.25	23.36	8.49	14.86	55.20	106.16 (70.07 to 138.01)
Greece	EL641	120,866	5.52	16.55	11.03	5.52	49.64	88.25 (60.67 to 118.59)
Greece	EL642	213,190	4.69	18.76	12.51	7.82	51.60	95.38 (64.11 to 126.65)
Greece	EL643	19,388	0.00	17.19	0.00	0.00	51.58	68.77 (68.77 to 120.35)
Greece	EL644	160,459	6.23	20.77	10.39	6.23	54.01	97.64 (66.48 to 130.87)
Greece	EL645	42,339	7.87	15.75	7.87	7.87	47.24	86.6 (62.98 to 118.09)
Greece	EL651	181,112	7.36	20.25	11.04	9.20	58.90	106.75 (69.94 to 139.88)
Greece	EL652	148,549	6.73	17.95	11.22	8.98	56.10	100.98 (67.32 to 132.39)
Greece	EL653	250,436	6.66	22.63	10.65	13.31	61.23	114.47 (75.87 to 150.4)
Spain	ES111	1,123,999	6.82	5.63	0.59	9.49	51.01	73.55 (49.23 to 97.86)
Spain	ES112	335,040	12.93	7.96	0.99	10.94	70.64	103.47 (68.65 to 136.3)
Spain	ES113	313,574	13.82	8.50	1.06	8.50	69.10	100.99 (68.03 to 135)

Spain	ES114	944,419	8.12	5.29	0.35	7.76	43.41	64.94 (43.41 to 86.47)
Spain	ES120	1,038,182	9.63	8.03	0.64	9.31	55.55	83.16 (55.87 to 111.09)
Spain	ES130	582,662	9.15	8.01	0.57	7.44	45.77	70.94 (47.48 to 94.39)
Spain	ES211	323,006	10.32	7.22	1.03	5.16	37.15	60.89 (40.25 to 80.49)
Spain	ES212	708,620	7.53	9.88	0.94	6.59	41.87	66.8 (44.69 to 88.91)
Spain	ES213	1,135,213	9.40	9.10	0.88	6.75	44.34	70.47 (47.27 to 93.96)
Spain	ES220	639,527	8.86	9.38	1.04	5.73	40.66	65.67 (43.78 to 87.56)
Spain	ES230	312,972	12.78	8.52	1.07	5.33	45.80	73.49 (48.99 to 95.86)
Spain	ES241	220,381	13.61	12.10	1.51	9.08	57.48	93.78 (62.01 to 124.03)
Spain	ES242	135,621	14.75	9.83	2.46	9.83	61.45	98.31 (68.82 to 135.18)
Spain	ES243	962,543	13.51	9.35	1.39	6.58	45.02	75.84 (50.56 to 100.77)
Spain	ES300	6,458,983	11.82	4.90	1.39	3.72	38.19	60.02 (40.15 to 79.73)
Spain	ES411	162,795	14.33	8.19	2.05	6.14	69.62	100.33 (67.57 to 135.14)
Spain	ES412	359,123	12.07	7.43	0.93	5.57	51.98	77.97 (52.91 to 104.89)
Spain	ES413	472,919	14.10	7.05	1.41	5.64	66.26	94.45 (63.44 to 125.46)
Spain	ES414	163,716	14.25	8.14	2.04	6.11	65.15	95.69 (63.12 to 126.23)
Spain	ES415	337,576	10.86	7.90	1.97	5.92	61.22	87.88 (59.25 to 116.52)
Spain	ES416	155,999	12.82	6.41	2.14	4.27	57.69	83.33 (57.69 to 113.25)
Spain	ES417	90,570	14.72	11.04	3.68	7.36	66.25	103.05 (66.25 to 132.49)
Spain	ES418	524,067	10.81	5.72	1.27	4.45	48.98	71.24 (47.7 to 94.77)
Spain	ES419	179,996	14.82	11.11	1.85	7.41	79.63	114.82 (77.78 to 153.71)
Spain	ES421	392,451	11.04	8.49	2.55	8.49	46.71	77.29 (51.81 to 103.62)
Spain	ES422	506,307	12.51	7.90	3.29	7.24	56.62	87.56 (57.94 to 115.21)
Spain	ES423	203,309	14.76	9.84	3.28	9.84	62.30	100.01 (67.22 to 132.8)
Spain	ES424	255,051	11.76	5.23	1.31	5.23	43.13	66.65 (45.74 to 90.18)
Spain	ES425	689,193	12.09	6.29	1.93	4.84	46.43	71.58 (47.88 to 95.28)
Spain	ES431	680,518	9.31	9.31	2.45	6.86	48.49	76.41 (50.94 to 101.88)
Spain	ES432	400,625	11.65	9.15	2.50	6.66	57.41	87.36 (58.24 to 116.48)
Spain	ES511	5,467,017	8.72	12.68	1.46	10.61	35.97	69.45 (46.46 to 92.31)
Spain	ES512	744,224	7.61	12.99	1.34	8.06	36.73	66.74 (44.79 to 88.68)
Spain	ES513	429,471	10.87	10.87	1.55	7.76	44.24	75.29 (50.45 to 100.12)
Spain	ES514	793,109	8.41	11.77	1.68	10.09	36.99	68.93 (45.81 to 90.78)
Spain	ES521	1,843,177	8.14	10.85	2.89	11.39	38.88	72.16 (48.11 to 95.49)
Spain	ES522	571,361	10.50	11.08	1.75	11.67	40.25	75.26 (50.76 to 100.93)
Spain	ES523	2,523,822	10.70	10.83	2.11	12.28	39.09	75.02 (50.19 to 99.72)
Spain	ES531	160,593	4.15	10.38	2.08	8.30	24.91	49.82 (33.21 to 66.42)
Spain	ES532	890,299	5.24	12.36	2.25	8.61	33.32	61.78 (41.18 to 81.99)
Spain	ES533	93,705	3.56	14.23	0.00	10.67	28.46	56.92 (35.57 to 74.7)
Spain	ES611	697,114	7.65	7.65	3.35	10.04	35.38	64.07 (43.03 to 85.11)
Spain	ES612	1,248,760	7.47	8.54	3.74	10.94	36.04	66.73 (44.58 to 88.62)

Spain	ES613	791,158	13.06	7.58	3.37	8.85	48.45	81.32 (54.35 to 108.28)
Spain	ES614	917,784	10.17	6.17	4.00	8.72	47.58	76.63 (51.57 to 102.06)
Spain	ES615	521,846	8.30	8.30	3.19	8.94	39.60	68.35 (45.35 to 90.7)
Spain	ES616	644,261	13.45	7.24	3.62	8.28	52.77	85.37 (57.43 to 113.83)
Spain	ES617	1,642,553	8.12	7.71	4.06	11.16	37.75	68.8 (46.07 to 91.32)
Spain	ES618	1,942,140	11.50	6.87	3.09	8.07	38.62	68.14 (45.65 to 90.79)
Spain	ES620	1,469,702	7.94	8.62	2.72	9.75	34.02	63.05 (41.96 to 83.46)
Finland	FI193	275,842	2.42	7.25	2.42	3.63	39.88	55.59 (37.46 to 73.71)
Finland	FI194	192,189	1.73	8.67	1.73	3.47	41.63	57.24 (38.16 to 78.05)
Finland	FI195	181,305	1.84	7.35	1.84	3.68	38.61	53.32 (34.93 to 69.86)
Finland	FI196	222,270	3.00	12.00	3.00	6.00	47.99	71.98 (47.99 to 94.48)
Finland	FI197	507,733	2.63	7.88	1.97	3.94	36.11	52.52 (35.45 to 70.25)
Finland	FI1B1	1,629,392	2.05	7.57	2.05	3.48	29.05	44.19 (29.46 to 58.71)
Finland	FI1C1	475,067	2.10	11.23	2.10	5.61	40.70	61.75 (41.4 to 82.09)
Finland	FI1C2	174,140	3.83	9.57	1.91	3.83	40.20	59.34 (40.2 to 80.4)
Finland	FI1C3	201,634	3.31	9.92	3.31	4.96	41.33	62.82 (41.33 to 82.66)
Finland	FI1C4	177,929	3.75	11.24	3.75	5.62	48.71	73.06 (48.71 to 97.42)
Finland	FI1C5	130,823	2.55	10.19	5.10	5.10	48.41	71.34 (45.86 to 94.27)
Finland	FI1D1	149,509	4.46	11.15	4.46	4.46	53.51	78.03 (51.28 to 102.56)
Finland	FI1D2	247,741	2.69	6.73	2.69	4.04	41.71	57.86 (39.02 to 78.04)
Finland	FI1D3	164,271	2.03	6.09	4.06	4.06	44.64	60.88 (40.58 to 83.2)
Finland	FI1D5	68,918	0.00	4.84	0.00	4.84	33.86	43.53 (29.02 to 58.04)
Finland	FI1D7	180,509	1.85	5.54	1.85	1.85	42.47	53.55 (36.93 to 72.02)
Finland	FI1D8	75,051	4.44	8.88	4.44	4.44	48.86	71.06 (44.41 to 88.83)
Finland	FI1D9	410,399	1.62	4.06	1.62	2.44	30.05	39.8 (26.8 to 53.61)
Finland	FI200	29,151	0.00	11.43	0.00	11.43	34.30	57.17 (34.3 to 80.04)
France	FR101	2,189,986	8.83	7.91	0.61	3.50	31.81	52.66 (35.31 to 70.17)
France	FR102	1,401,075	6.42	5.71	0.48	2.38	22.36	37.35 (24.98 to 49.72)
France	FR103	1,434,691	6.04	5.34	0.46	2.56	21.84	36.24 (24.16 to 48.09)
France	FR104	1,289,084	6.46	5.69	0.52	2.59	23.27	38.53 (25.86 to 51.46)
France	FR105	1,608,316	7.05	6.42	0.41	2.90	25.49	42.28 (28.19 to 56.17)
France	FR106	1,613,778	5.37	4.96	0.41	2.27	19.21	32.22 (21.48 to 42.55)
France	FR107	1,383,845	7.71	6.74	0.48	3.13	27.22	45.28 (30.35 to 60.22)
France	FR108	1,226,128	6.52	5.98	0.54	2.72	23.65	39.42 (26.37 to 52.2)
France	FRB01	305,942	10.90	9.81	1.09	4.36	43.58	69.73 (46.85 to 93.7)
France	FRB02	433,299	8.46	7.69	0.77	3.85	34.62	55.39 (36.93 to 73.85)
France	FRB03	222,633	11.98	10.48	1.50	5.99	50.91	80.85 (53.9 to 107.8)
France	FRB04	606,365	9.35	8.25	0.55	4.40	39.03	61.57 (41.23 to 82.46)
France	FRB05	331,996	10.04	9.04	1.00	5.02	41.17	66.27 (44.18 to 88.35)
France	FRB06	676,157	9.37	8.38	0.49	3.94	34.51	56.69 (37.47 to 74.93)

France	FRC11	533,350	11.87	11.87	1.25	3.75	38.75	67.5 (45 to 89.37)
France	FRC12	208,480	14.39	12.79	1.60	4.80	52.76	86.34 (57.56 to 115.12)
France	FRC13	554,053	13.84	12.03	1.20	4.21	40.91	72.2 (48.13 to 95.66)
France	FRC14	339,311	11.79	11.79	0.98	4.91	44.21	73.68 (49.12 to 97.26)
France	FRC21	539,007	11.13	12.99	0.62	3.09	34.63	62.46 (42.05 to 83.49)
France	FRC22	260,260	11.53	11.53	1.28	3.84	34.58	62.76 (42.27 to 83.25)
France	FRC23	236,906	11.26	11.26	1.41	2.81	33.77	60.5 (40.8 to 80.2)
France	FRC24	143,262	13.96	16.29	0.00	4.65	41.88	76.78 (51.19 to 100.05)
France	FRD11	693,829	6.25	9.61	0.48	6.25	45.64	68.22 (45.64 to 90.8)
France	FRD12	497,629	5.36	9.38	0.67	6.70	46.89	68.99 (46.22 to 92.44)
France	FRD13	284,223	8.21	9.38	0.00	5.86	42.22	65.68 (43.39 to 86.79)
France	FRD21	601,645	6.09	6.65	0.55	3.32	28.26	44.88 (30.47 to 60.39)
France	FRD22	1,255,929	7.17	10.09	0.53	5.57	40.08	63.43 (42.47 to 84.4)
France	FRE11	2,604,889	6.14	12.28	0.64	4.48	35.06	58.61 (39.03 to 77.67)
France	FRE12	1,469,534	6.12	12.02	0.68	5.22	38.33	62.38 (41.51 to 82.79)
France	FRE21	535,650	9.33	11.82	0.62	4.36	37.34	63.47 (42.94 to 84.63)
France	FRE22	824,188	6.88	7.68	0.40	3.24	27.50	45.7 (30.74 to 61.07)
France	FRE23	571,932	8.16	12.24	0.58	5.83	41.96	68.77 (46.04 to 90.92)
France	FRF11	1,124,294	9.49	14.23	0.59	2.67	29.06	56.04 (37.65 to 74.71)
France	FRF12	763,590	10.91	14.41	0.87	3.06	32.74	61.99 (41.47 to 82.07)
France	FRF21	274,637	8.50	13.35	1.21	3.64	35.20	61.9 (40.05 to 81.32)
France	FRF22	309,502	10.77	10.77	1.08	4.31	36.62	63.54 (42 to 82.93)
France	FRF23	569,883	10.53	12.28	0.58	4.09	38.60	66.1 (44.45 to 88.32)
France	FRF24	176,737	11.32	13.20	0.00	3.77	41.49	69.78 (47.15 to 96.19)
France	FRF31	733,794	10.45	13.63	0.91	3.63	36.34	64.96 (43.15 to 85.86)
France	FRF32	188,056	10.64	12.41	0.00	3.55	35.45	62.04 (42.54 to 85.08)
France	FRF33	1,044,201	9.58	15.00	0.96	3.19	35.11	63.84 (42.78 to 84.91)
France	FRF34	368,861	12.65	15.36	0.90	3.61	40.67	73.2 (48.8 to 96.69)
France	FRG01	1,388,373	6.96	6.24	0.48	5.28	37.21	56.18 (37.69 to 74.91)
France	FRG02	812,624	8.20	6.97	0.41	4.92	37.33	57.84 (38.56 to 76.71)
France	FRG03	307,539	7.59	6.50	0.00	4.34	36.85	55.28 (37.94 to 75.87)
France	FRG04	567,119	8.82	8.23	0.59	4.70	39.97	62.3 (42.32 to 83.46)
France	FRG05	673,137	8.42	7.43	0.50	6.44	42.59	65.37 (43.58 to 86.66)
France	FRH01	598,927	6.12	8.35	0.56	7.79	51.20	74.02 (49.53 to 97.95)
France	FRH02	909,202	5.13	7.33	0.73	8.80	54.26	76.26 (50.59 to 100.82)
France	FRH03	1,056,023	6.00	6.31	0.63	5.05	35.98	53.98 (35.98 to 71.65)
France	FRH04	749,523	6.67	7.56	0.44	7.12	48.92	70.71 (47.59 to 94.28)
France	FRI11	414,308	12.87	10.46	0.80	5.63	49.08	78.85 (52.3 to 104.59)
France	FRI12	1,575,097	9.10	7.83	0.63	5.29	38.73	61.58 (41.27 to 82.11)
France	FRI13	406,511	8.20	9.02	0.82	5.74	41.00	64.78 (42.64 to 84.46)

France	FRI14	332,766	12.02	10.02	1.00	6.01	47.08	76.13 (51.09 to 101.17)
France	FRI15	675,284	9.38	10.86	0.99	5.92	46.40	73.55 (49.36 to 98.23)
France	FRI21	241,363	12.43	12.43	1.38	5.52	53.86	85.62 (58 to 116.01)
France	FRI22	119,002	14.01	14.01	0.00	5.60	58.82	92.44 (61.62 to 123.25)
France	FRI23	374,600	12.46	11.57	0.89	6.23	49.83	80.98 (53.39 to 106.78)
France	FRI31	352,754	11.34	9.45	0.94	5.67	47.25	74.65 (50.08 to 100.16)
France	FRI32	643,341	9.84	9.33	1.04	7.25	50.26	77.72 (51.81 to 103.11)
France	FRI33	374,582	8.90	8.01	0.89	5.34	40.93	64.07 (42.71 to 84.54)
France	FRI34	436,355	9.93	8.40	0.76	5.35	42.78	67.22 (45.07 to 89.38)
France	FRJ11	369,512	12.63	13.53	0.90	8.12	46.91	82.09 (55.93 to 110.06)
France	FRJ12	742,458	12.12	13.02	1.35	6.29	39.96	72.73 (48.94 to 96.98)
France	FRJ13	1,139,196	11.12	13.46	1.17	7.90	43.60	77.25 (51.79 to 102.7)
France	FRJ14	76,463	13.08	17.44	0.00	4.36	56.67	91.55 (61.03 to 122.06)
France	FRJ15	474,054	11.95	16.17	1.41	9.14	52.74	91.41 (61.17 to 121.65)
France	FRJ21	152,946	10.90	10.90	2.18	6.54	47.95	78.46 (50.13 to 102.43)
France	FRJ22	279,087	11.94	14.33	1.19	5.97	52.55	85.99 (57.33 to 114.66)
France	FRJ23	1,356,658	9.34	7.86	0.74	4.67	31.70	54.3 (36.12 to 71.99)
France	FRJ24	190,993	10.47	8.73	0.00	5.24	41.89	66.32 (45.38 to 92.5)
France	FRJ25	173,626	11.52	11.52	1.92	5.76	49.92	80.63 (53.76 to 107.51)
France	FRJ26	228,533	11.67	13.13	1.46	5.83	53.97	86.06 (56.88 to 113.77)
France	FRJ27	387,369	11.19	12.05	0.86	6.02	43.03	73.14 (49.05 to 98.1)
France	FRJ28	257,411	10.36	9.06	1.29	5.18	38.85	64.75 (42.73 to 85.47)
France	FRK11	339,039	13.76	11.80	0.98	4.92	50.14	81.6 (55.06 to 109.13)
France	FRK12	145,524	13.74	16.03	2.29	6.87	61.85	100.79 (64.14 to 130.56)
France	FRK13	227,302	10.27	11.73	1.47	4.40	42.53	70.39 (46.93 to 93.85)
France	FRK14	652,748	10.72	10.21	1.02	4.09	41.87	67.92 (45.45 to 90.9)
France	FRK21	640,322	9.89	8.33	0.52	2.60	26.03	47.37 (31.75 to 63.51)
France	FRK22	325,421	15.36	13.32	1.02	5.12	43.02	77.85 (52.24 to 102.43)
France	FRK23	509,732	14.39	11.77	1.31	4.58	35.31	67.36 (45.12 to 89.59)
France	FRK24	1,256,564	10.35	8.75	0.80	2.92	28.12	50.93 (34.22 to 67.91)
France	FRK25	761,948	12.25	10.94	1.31	4.37	41.12	70 (46.81 to 92.74)
France	FRK26	1,840,185	11.59	8.88	0.91	3.26	30.25	54.89 (36.59 to 72.82)
France	FRK27	430,696	11.61	11.61	0.77	3.10	37.15	64.24 (42.57 to 85.13)
France	FRK28	804,853	8.70	10.77	0.83	2.90	30.65	53.84 (35.62 to 71.23)
France	FRL01	163,087	12.26	18.40	2.04	6.13	51.10	89.93 (59.27 to 118.55)
France	FRL02	141,001	9.46	14.18	2.36	4.73	54.37	85.11 (56.74 to 113.47)
France	FRL03	1,083,918	11.07	24.29	2.15	12.61	48.59	98.72 (66.12 to 131.31)
France	FRL04	2,023,715	12.35	15.81	1.48	7.91	39.20	76.76 (51.56 to 102.29)
France	FRL05	1,057,728	12.29	20.48	1.89	10.08	46.64	91.39 (61.14 to 121.33)
France	FRL06	558,959	15.51	16.10	1.79	6.56	43.53	83.49 (55.46 to 110.32)

France	FRM01	155,534	8.57	17.15	2.14	10.72	40.72	79.3 (53.58 to 105.01)
France	FRM02	177,274	7.52	20.68	1.88	11.28	41.37	82.73 (54.53 to 107.18)
Croatia	HR	4,168,923	4.96	32.86	5.36	5.68	49.57	98.43 (65.8 to 130.81)
Hungary	HU110	1,754,866	7.41	30.39	4.75	3.61	48.06	94.21 (63.25 to 125.56)
Hungary	HU120	1,242,473	6.71	27.90	4.56	3.22	44.27	86.66 (57.95 to 115.09)
Hungary	HU211	417,261	6.39	31.16	4.79	3.99	48.73	95.06 (63.11 to 126.22)
Hungary	HU212	297,965	5.59	34.68	4.47	4.47	51.46	100.68 (68.24 to 134.24)
Hungary	HU213	343,719	5.82	36.85	4.85	3.88	52.37	103.77 (69.82 to 137.71)
Hungary	HU221	456,679	3.65	31.39	3.65	3.65	42.33	84.67 (56.2 to 111.68)
Hungary	HU222	253,525	3.94	36.81	3.94	3.94	48.65	97.29 (64.42 to 128.85)
Hungary	HU223	273,937	4.87	36.50	4.87	4.87	49.89	101 (66.93 to 133.85)
Hungary	HU231	367,173	5.45	33.59	5.45	4.54	51.75	100.77 (68.09 to 134.36)
Hungary	HU232	307,925	5.41	36.81	5.41	4.33	54.13	106.09 (71.45 to 141.81)
Hungary	HU233	222,668	5.99	32.93	5.99	4.49	52.39	101.8 (68.86 to 136.23)
Hungary	HU311	657,690	5.58	31.93	6.08	3.55	52.20	99.34 (66.39 to 132.28)
Hungary	HU312	298,309	6.70	32.40	5.59	4.47	53.64	102.8 (68.16 to 136.32)
Hungary	HU313	193,345	6.90	36.20	5.17	3.45	56.89	108.61 (72.41 to 144.82)
Hungary	HU321	533,776	4.37	24.98	5.62	3.12	44.34	82.43 (54.95 to 109.28)
Hungary	HU322	375,283	7.11	32.86	6.22	4.44	55.07	105.7 (70.17 to 139.45)
Hungary	HU323	561,463	3.56	24.34	6.53	2.97	43.93	81.34 (54.62 to 108.64)
Hungary	HU331	509,681	6.54	31.39	5.89	3.92	50.36	98.1 (65.4 to 130.15)
Hungary	HU332	344,667	5.80	34.82	7.74	3.87	59.96	112.19 (75.44 to 148.94)
Hungary	HU333	403,093	4.96	31.42	6.62	4.13	50.44	97.58 (65.33 to 129)
Ireland	IE041	395,752	0.84	3.37	0.00	1.68	32.01	37.9 (26.11 to 52.22)
Ireland	IE042	448,666	0.74	2.97	0.00	1.49	31.95	37.15 (25.26 to 50.52)
Ireland	IE051	475,790	1.40	3.50	0.00	2.10	32.23	39.23 (26.62 to 52.54)
Ireland	IE052	421,843	1.58	3.95	0.00	2.37	30.82	38.72 (26.08 to 52.15)
Ireland	IE053	687,191	0.97	3.40	0.49	2.43	33.47	40.75 (26.68 to 53.36)
Ireland	IE061	1,337,275	1.50	3.49	0.25	1.99	25.92	33.15 (21.94 to 43.87)
Ireland	IE062	693,131	1.44	2.89	0.00	1.44	21.64	27.41 (18.76 to 37.03)
Ireland	IE063	295,026	1.13	3.39	0.00	2.26	28.25	35.03 (23.73 to 46.32)
Iceland	IS001	216,066	0.00	1.54	0.00	1.54	30.85	33.94 (23.14 to 46.28)
Iceland	IS002	121,041	0.00	2.75	0.00	2.75	38.55	44.06 (27.54 to 55.08)
Italy	ITC11	2,280,223	26.75	16.52	1.61	5.26	45.17	95.31 (63.88 to 126.74)
Italy	ITC12	174,300	30.60	22.95	1.91	7.65	53.55	116.66 (78.41 to 152.99)
Italy	ITC13	179,098	29.78	20.47	1.86	5.58	57.70	115.39 (78.17 to 156.34)
Italy	ITC14	159,955	25.01	22.92	2.08	6.25	56.27	112.53 (75.02 to 147.96)
Italy	ITC15	370,420	23.40	18.00	1.80	5.40	42.29	90.89 (61.19 to 121.48)
Italy	ITC16	590,037	22.60	18.08	1.69	6.21	49.71	98.3 (66.1 to 131.07)
Italy	ITC17	217,357	27.60	21.47	1.53	9.20	53.68	113.48 (76.68 to 153.36)

Italy	ITC18	427,886	25.71	26.49	2.34	12.46	57.65	124.64 (83.36 to 165.93)
Italy	ITC20	127,178	10.48	18.35	2.62	5.24	65.52	102.22 (68.15 to 133.67)
Italy	ITC31	215,374	17.02	27.86	3.10	15.48	58.81	122.27 (80.48 to 160.96)
Italy	ITC32	280,133	20.23	27.37	2.38	15.47	59.50	124.94 (83.29 to 165.4)
Italy	ITC33	852,826	19.15	28.92	3.13	17.59	57.85	126.64 (84.82 to 168.07)
Italy	ITC34	220,818	16.60	27.17	3.02	15.10	54.34	116.23 (76.99 to 153.97)
Italy	ITC41	890,224	22.47	17.97	1.87	5.24	41.19	88.74 (59.16 to 117.57)
Italy	ITC42	599,763	23.34	18.34	1.67	5.00	41.68	90.04 (60.58 to 120.05)
Italy	ITC43	339,532	23.56	16.69	1.96	4.91	40.25	87.38 (57.92 to 114.86)
Italy	ITC44	181,660	16.51	18.35	1.83	3.67	53.21	93.58 (62.39 to 122.94)
Italy	ITC46	1,109,530	21.93	15.32	1.80	4.51	36.65	80.21 (53.18 to 105.75)
Italy	ITC47	1,263,566	18.99	15.30	1.85	4.22	35.09	75.45 (50.39 to 99.98)
Italy	ITC48	547,427	24.36	21.31	2.44	7.92	48.10	104.12 (69.42 to 138.22)
Italy	ITC49	229,523	18.88	15.98	1.45	5.81	36.31	78.42 (52.28 to 104.56)
Italy	ITC4A	359,989	21.30	18.52	1.85	5.56	40.74	87.97 (59.26 to 117.6)
Italy	ITC4B	413,040	20.18	18.56	2.42	5.65	39.54	86.35 (57.3 to 113.79)
Italy	ITC4C	3,214,548	21.67	17.01	1.76	5.70	38.68	84.82 (56.72 to 112.61)
Italy	ITC4D	867,798	21.13	15.75	1.54	4.61	36.11	79.13 (53.01 to 104.86)
Italy	ITF11	302,609	13.22	20.93	4.41	8.81	55.08	102.44 (68.29 to 135.49)
Italy	ITF12	309,913	11.83	20.44	4.30	8.60	47.33	92.5 (62.38 to 123.69)
Italy	ITF13	321,357	12.45	19.71	4.15	8.30	47.71	92.32 (62.24 to 122.4)
Italy	ITF14	390,004	13.68	22.22	5.13	10.26	52.14	103.42 (69.23 to 137.61)
Italy	ITF21	86,069	15.49	23.24	3.87	11.62	58.09	112.31 (73.58 to 143.3)
Italy	ITF22	225,011	13.33	22.22	5.93	10.37	53.33	105.18 (69.63 to 139.25)
Italy	ITF31	924,160	11.54	15.15	3.25	9.74	37.15	76.83 (51.58 to 102.44)
Italy	ITF32	280,458	16.64	21.39	4.75	10.70	51.11	104.59 (70.12 to 139.06)
Italy	ITF33	3,110,014	11.68	14.90	3.32	10.18	36.44	76.53 (51.23 to 101.82)
Italy	ITF34	424,573	15.70	19.63	4.71	10.99	48.68	99.71 (66.73 to 132.68)
Italy	ITF35	1,105,377	12.06	16.89	4.22	10.86	42.52	86.55 (58.2 to 115.19)
Italy	ITF43	584,557	10.83	19.39	5.70	9.69	43.34	88.96 (59.3 to 118.04)
Italy	ITF44	397,861	9.22	20.95	5.86	10.89	44.40	91.32 (62 to 123.16)
Italy	ITF45	802,906	9.55	22.00	6.64	12.45	46.91	97.56 (65.6 to 129.94)
Italy	ITF46	629,639	10.59	19.06	4.76	9.00	42.88	86.29 (57.71 to 114.35)
Italy	ITF47	1,261,965	8.98	17.96	5.02	9.24	39.36	80.56 (53.88 to 107.24)
Italy	ITF48	392,923	8.48	16.12	4.24	7.64	36.48	72.96 (48.36 to 96.71)
Italy	ITF51	371,836	11.65	19.72	5.38	9.86	51.99	98.61 (66.34 to 131.78)
Italy	ITF52	200,114	11.66	18.32	5.00	8.33	48.31	91.61 (61.63 to 121.6)
Italy	ITF61	713,094	9.82	17.30	5.14	10.75	47.21	90.22 (60.77 to 120.6)
Italy	ITF62	174,917	9.53	15.25	3.81	9.53	41.92	80.04 (53.36 to 106.72)
Italy	ITF63	362,483	9.20	15.63	4.60	11.95	43.22	84.6 (57.01 to 113.11)

Italy	ITF64	162,023	10.29	16.46	4.11	14.40	43.20	88.46 (59.66 to 117.27)
Italy	ITF65	554,726	9.61	16.22	4.81	13.82	43.26	87.73 (58.89 to 117.17)
Italy	ITG11	434,734	9.20	19.17	5.37	15.34	47.54	96.61 (65.17 to 128.81)
Italy	ITG12	1,269,085	8.67	16.02	4.47	12.61	42.55	84.31 (56.47 to 112.42)
Italy	ITG13	638,480	10.96	18.79	5.22	16.71	50.12	101.8 (68.39 to 135.74)
Italy	ITG14	443,298	9.78	18.80	5.26	15.04	51.13	100.01 (66.92 to 133.09)
Italy	ITG15	270,480	9.86	17.25	4.93	14.79	50.53	97.36 (65.32 to 130.63)
Italy	ITG16	168,821	11.85	19.74	5.92	13.82	59.23	110.57 (73.06 to 146.11)
Italy	ITG17	1,113,911	9.58	14.36	4.49	11.67	44.29	84.39 (56.56 to 112.52)
Italy	ITG18	320,485	8.32	14.56	5.20	15.60	40.56	84.25 (57.2 to 113.37)
Italy	ITG19	403,200	10.75	15.71	4.96	14.88	45.47	91.77 (61.18 to 121.53)
Italy	ITG2D	493,892	7.42	20.25	2.70	10.80	42.52	83.69 (56.02 to 110.69)
Italy	ITG2E	561,010	2.97	8.32	1.19	4.16	19.01	35.65 (23.77 to 47.53)
Italy	ITG2F	161,255	18.60	45.48	10.34	26.87	103.36	204.64 (136.43 to 272.86)
Italy	ITG2G	213,886	6.23	17.14	3.12	7.79	37.40	71.69 (48.31 to 95.07)
Italy	ITG2H	225,641	13.30	33.98	5.91	19.20	75.34	147.73 (98.98 to 196.48)
Italy	ITH10	522,854	8.93	14.03	1.28	2.55	36.98	63.75 (43.35 to 86.07)
Italy	ITH20	538,535	14.86	16.09	1.86	3.71	39.61	76.13 (50.75 to 100.27)
Italy	ITH31	922,606	18.79	17.70	2.17	4.70	37.21	80.57 (53.83 to 106.94)
Italy	ITH32	866,330	16.93	16.93	1.92	4.23	35.01	75.03 (50.02 to 99.65)
Italy	ITH33	206,358	19.38	24.23	3.23	4.85	53.31	105 (69.46 to 138.92)
Italy	ITH34	886,533	14.66	17.30	1.88	4.51	34.22	72.57 (48.88 to 96.63)
Italy	ITH35	855,430	15.59	21.04	2.73	6.62	40.53	86.51 (57.67 to 114.56)
Italy	ITH36	937,049	16.01	17.79	2.13	4.98	35.57	76.48 (51.22 to 101.74)
Italy	ITH37	239,515	18.09	23.66	2.78	6.96	47.32	98.81 (66.8 to 133.6)
Italy	ITH41	312,724	13.86	20.25	2.13	5.33	38.37	79.94 (53.3 to 105.52)
Italy	ITH42	532,577	13.77	25.66	3.13	6.26	46.32	95.13 (63.84 to 126.43)
Italy	ITH43	140,069	14.28	30.94	2.38	7.14	52.36	107.09 (71.39 to 140.41)
Italy	ITH44	235,067	11.34	35.45	4.25	8.51	56.72	116.28 (77.99 to 154.57)
Italy	ITH51	287,137	23.22	20.90	2.32	6.97	47.60	101 (68.49 to 134.66)
Italy	ITH52	448,082	20.83	20.09	2.23	6.70	43.89	93.73 (62.49 to 124.23)
Italy	ITH53	532,795	18.14	18.14	1.88	6.26	38.79	83.21 (55.06 to 110.11)
Italy	ITH54	701,691	18.05	18.53	2.38	6.18	39.90	85.03 (56.53 to 112.59)
Italy	ITH55	1,007,664	18.19	21.50	2.98	7.61	45.65	95.93 (64.17 to 127.69)
Italy	ITH56	350,212	20.94	26.65	3.81	9.52	54.25	115.17 (77.1 to 152.29)
Italy	ITH57	391,570	15.32	23.84	3.41	9.36	47.67	99.6 (66.4 to 131.1)
Italy	ITH58	394,688	12.67	21.11	3.38	8.45	43.92	89.52 (59.96 to 119.93)
Italy	ITH59	336,193	10.91	19.83	2.97	7.93	40.65	82.29 (55.52 to 110.06)
Italy	ITI11	197,389	16.89	25.33	3.38	13.51	52.35	111.46 (74.3 to 146.92)
Italy	ITI12	391,011	16.20	24.72	3.41	12.79	51.15	108.27 (71.61 to 142.37)

Italy	ITI13	292,051	14.84	21.69	2.28	10.27	45.65	94.73 (65.06 to 127.83)
Italy	ITI14	1,013,303	15.13	21.38	2.96	9.87	45.73	95.07 (63.49 to 125.66)
Italy	ITI15	254,197	13.11	19.67	2.62	7.87	39.34	82.61 (55.08 to 110.15)
Italy	ITI16	337,643	13.82	26.66	2.96	16.78	52.32	112.54 (76.02 to 151.05)
Italy	ITI17	421,333	13.45	21.36	2.37	11.87	43.51	92.56 (61.71 to 123.42)
Italy	ITI18	344,844	12.57	21.27	2.90	8.70	47.36	92.8 (62.83 to 124.69)
Italy	ITI19	269,006	13.63	23.54	3.72	11.15	53.28	105.33 (70.63 to 138.78)
Italy	ITI1A	223,338	11.94	25.37	4.48	14.93	53.73	110.45 (73.13 to 146.27)
Italy	ITI21	661,185	12.10	20.67	3.53	8.57	47.39	92.26 (62.01 to 122.51)
Italy	ITI22	228,688	14.58	21.86	4.37	10.20	55.39	106.4 (71.42 to 141.39)
Italy	ITI31	361,438	11.07	22.13	3.69	8.30	45.19	90.38 (59.95 to 119.89)
Italy	ITI32	475,203	11.92	23.85	4.21	10.52	49.80	100.31 (66.64 to 132.57)
Italy	ITI33	319,361	12.53	24.01	4.18	9.39	51.14	101.24 (67.84 to 133.6)
Italy	ITI34	209,790	12.71	23.83	4.77	9.53	50.84	101.69 (68.32 to 135.06)
Italy	ITI35	175,298	11.41	22.82	3.80	11.41	51.34	100.78 (68.45 to 135.01)
Italy	ITI41	319,862	12.51	20.84	4.17	11.46	48.98	97.96 (65.65 to 130.26)
Italy	ITI42	157,856	16.89	23.23	4.22	10.56	57.01	111.92 (73.91 to 147.81)
Italy	ITI43	4,347,996	12.57	16.56	3.22	10.35	40.94	83.64 (55.89 to 111.01)
Italy	ITI44	574,292	11.03	15.67	3.48	11.03	38.89	80.1 (53.4 to 105.64)
Italy	ITI45	493,924	14.17	18.22	4.05	10.12	49.94	96.51 (64.11 to 127.55)
Liechtenstein	LI000	37,728	0.00	17.67	0.00	0.00	26.51	44.18 (35.34 to 70.68)
Lithuania	LT011	805,861	2.90	16.13	7.03	4.55	45.50	76.11 (50.46 to 100.93)
Lithuania	LT021	143,157	2.33	23.28	9.31	6.99	60.54	102.45 (67.52 to 132.72)
Lithuania	LT022	573,348	3.49	19.77	6.98	5.81	52.32	88.37 (58.72 to 116.86)
Lithuania	LT023	322,419	2.07	21.71	5.17	8.27	52.73	89.95 (59.96 to 118.89)
Lithuania	LT024	146,879	2.27	22.69	6.81	6.81	56.74	95.32 (65.81 to 129.36)
Lithuania	LT025	227,539	4.39	20.51	7.32	5.86	58.60	96.69 (64.46 to 127.45)
Lithuania	LT026	273,478	3.66	21.94	6.09	7.31	57.29	96.29 (63.38 to 125.54)
Lithuania	LT027	99,677	3.34	23.41	6.69	6.69	60.19	100.32 (66.88 to 133.77)
Lithuania	LT028	139,178	2.40	21.56	4.79	7.19	50.30	86.22 (57.48 to 114.96)
Lithuania	LT029	135,121	4.93	24.67	9.87	7.40	69.07	115.95 (76.47 to 152.95)
Luxembourg	LU000	582,970	0.57	17.15	0.57	2.29	26.30	46.89 (31.45 to 62.32)
Latvia	LV003	248,790	1.34	24.12	5.36	9.38	58.95	99.15 (65.65 to 131.3)
Latvia	LV005	273,297	2.44	19.51	8.54	6.10	58.54	95.13 (63.42 to 125.63)
Latvia	LV006	640,008	3.12	19.27	5.21	6.77	53.12	87.5 (58.33 to 116.14)
Latvia	LV007	366,544	2.73	17.28	4.55	6.37	49.11	80.03 (53.65 to 106.4)
Latvia	LV008	193,828	3.44	20.64	6.88	6.88	67.07	104.9 (70.51 to 139.3)
Latvia	LV009	237,421	2.81	16.85	5.62	5.62	47.74	78.62 (51.95 to 103.89)
Montenegro	ME000	622,266	1.61	23.03	6.43	4.82	46.60	82.49 (55.71 to 110.89)
Malta	MT	456,526	0.73	16.79	3.65	15.33	27.02	63.52 (42.35 to 83.97)

Netherlands	NL11	583,547	2.86	16.57	1.14	5.14	37.13	62.83 (41.7 to 82.83)
Netherlands	NL12	646,610	3.09	16.50	1.03	5.16	38.66	64.44 (42.79 to 85.57)
Netherlands	NL13	490,274	4.08	19.04	0.68	5.44	40.11	69.35 (46.23 to 91.79)
Netherlands	NL21	1,146,030	3.78	16.29	0.58	4.07	32.29	57.01 (38.39 to 76.21)
Netherlands	NL22	2,042,483	4.24	16.48	0.65	3.75	33.13	58.26 (39 to 77.52)
Netherlands	NL23	406,337	2.46	10.66	0.82	3.28	22.97	40.2 (26.25 to 51.68)
Netherlands	NL31	1,279,293	3.39	13.03	0.52	3.39	29.18	49.51 (33.09 to 65.92)
Netherlands	NL32	2,796,862	2.86	14.54	0.72	4.41	33.73	56.25 (37.66 to 74.85)
Netherlands	NL33	3,638,395	2.66	13.47	0.64	3.94	31.52	52.22 (34.91 to 69.44)
Netherlands	NL34	381,463	1.75	17.48	0.87	4.37	39.32	63.79 (42.82 to 84.76)
Netherlands	NL41	2,507,079	3.46	17.55	0.66	3.72	33.37	58.77 (39.36 to 78.31)
Netherlands	NL42	1,117,236	2.98	23.87	0.90	4.18	39.08	71.01 (47.44 to 93.98)
Norway	NO020	385,178	2.60	6.92	0.87	3.46	42.40	56.25 (38.08 to 76.16)
Norway	NO060	452,158	2.21	4.42	0.74	2.95	33.91	44.23 (29.49 to 58.24)
Norway	NO071	242,430	1.37	4.12	1.37	2.75	41.25	50.87 (33 to 67.37)
Norway	NO074	240,853	1.38	2.77	1.38	2.77	34.60	42.9 (27.68 to 55.36)
Norway	NO081	661,395	2.02	4.54	0.50	2.52	23.69	33.26 (22.18 to 44.85)
Norway	NO082	1,169,209	2.28	6.27	0.86	3.14	30.50	43.05 (28.79 to 57.3)
Norway	NO091	418,699	2.39	7.96	0.80	3.98	36.62	51.75 (35.03 to 69.26)
Norway	NO092	299,662	1.11	7.79	1.11	4.45	34.48	48.94 (32.26 to 65.63)
Norway	NO0A1	470,414	1.42	6.38	0.71	3.54	28.34	40.39 (26.93 to 53.85)
Norway	NO0A2	627,286	1.06	2.66	0.53	2.13	17.54	23.91 (15.94 to 31.35)
Norway	NO0A3	265,505	1.26	5.02	1.26	3.77	38.92	50.22 (33.9 to 67.8)
Poland	PL213	756,456	10.13	17.63	3.08	2.64	35.25	68.74 (46.27 to 92.1)
Poland	PL214	712,955	9.35	16.83	3.27	2.34	33.20	64.99 (43.48 to 86.49)
Poland	PL217	452,781	8.83	15.46	3.68	2.21	31.66	61.84 (41.23 to 81.72)
Poland	PL218	530,484	6.91	15.71	3.14	2.51	30.16	58.44 (38.96 to 77.92)
Poland	PL219	334,664	6.97	17.93	2.99	2.99	33.86	64.74 (43.83 to 86.65)
Poland	PL21A	548,638	9.72	20.05	3.04	3.04	37.67	73.52 (49.21 to 97.21)
Poland	PL224	515,588	11.64	21.98	3.88	3.88	43.96	85.34 (56.89 to 112.49)
Poland	PL225	659,138	8.09	22.25	3.03	3.03	37.93	74.34 (49.56 to 98.11)
Poland	PL227	623,800	8.02	20.84	2.67	2.67	35.27	69.47 (46.49 to 92.44)
Poland	PL228	434,639	10.74	22.24	3.07	3.07	41.41	80.53 (54.45 to 107.37)
Poland	PL229	467,845	9.97	20.66	2.85	2.85	37.05	73.39 (49.16 to 97.61)
Poland	PL22A	741,280	12.14	24.28	3.60	3.60	45.42	89.04 (59.81 to 118.71)
Poland	PL22B	685,165	12.16	23.35	3.89	3.41	45.24	88.06 (59.35 to 117.73)
Poland	PL22C	389,462	8.56	17.97	2.57	2.57	33.38	65.05 (43.65 to 86.44)
Poland	PL411	407,502	7.36	15.54	2.45	4.09	36.81	66.26 (44.17 to 88.34)
Poland	PL414	654,157	8.66	16.82	2.55	3.57	36.18	67.77 (45.35 to 89.68)
Poland	PL415	540,463	8.63	19.12	2.47	4.32	40.09	74.63 (49.34 to 98.68)

Poland	PL416	666,561	8.50	18.50	2.50	3.50	37.51	70.51 (47.01 to 94.01)
Poland	PL417	551,520	6.65	16.92	1.81	3.02	33.24	61.65 (41.7 to 82.8)
Poland	PL418	635,230	6.30	14.17	1.57	3.15	29.39	54.57 (36.21 to 71.89)
Poland	PL424	403,381	5.78	19.83	1.65	5.78	42.97	76.02 (51.23 to 100.81)
Poland	PL426	351,498	4.74	16.12	1.90	5.69	39.83	68.28 (46.47 to 91.99)
Poland	PL427	423,521	6.30	18.10	2.36	4.72	40.93	72.41 (48.01 to 96.02)
Poland	PL428	504,757	4.62	17.17	1.98	4.62	36.32	64.72 (43.59 to 86.51)
Poland	PL431	380,800	6.13	19.26	1.75	4.38	36.76	68.28 (44.64 to 89.29)
Poland	PL432	624,606	5.87	20.81	1.60	3.74	36.29	68.31 (45.9 to 91.26)
Poland	PL514	632,405	7.91	21.61	2.11	3.16	38.48	73.27 (49.02 to 97.51)
Poland	PL515	559,248	5.96	27.42	2.38	3.58	42.91	82.25 (55.43 to 109.67)
Poland	PL516	445,209	6.74	20.22	2.25	2.99	35.94	68.13 (45.67 to 90.59)
Poland	PL517	652,640	7.15	28.60	2.55	4.09	46.48	88.87 (59.76 to 118.49)
Poland	PL518	576,634	6.94	19.08	2.31	2.89	34.11	65.32 (43.93 to 87.29)
Poland	PL523	367,241	8.17	24.51	2.72	3.63	42.66	81.69 (54.46 to 108.92)
Poland	PL524	585,006	9.12	20.51	2.85	3.42	37.61	73.5 (48.43 to 96.87)
Poland	PL613	769,124	8.23	14.30	2.60	3.90	35.54	64.58 (43.34 to 85.81)
Poland	PL616	385,657	8.64	14.69	3.46	4.32	38.03	69.15 (45.81 to 90.75)
Poland	PL617	361,881	8.29	15.66	2.76	3.68	36.84	67.24 (45.13 to 89.35)
Poland	PL618	186,516	7.15	14.30	1.79	3.57	35.74	62.55 (42.89 to 85.78)
Poland	PL619	358,867	10.22	16.72	2.79	3.72	41.80	75.24 (50.16 to 100.32)
Poland	PL621	521,976	7.66	14.05	3.19	4.47	38.32	67.69 (44.7 to 89.4)
Poland	PL622	608,978	7.12	12.59	3.83	4.38	36.67	64.59 (43.24 to 85.94)
Poland	PL623	282,129	5.91	11.81	4.73	3.54	36.63	62.62 (42.53 to 83.89)
Poland	PL633	742,156	6.29	15.72	3.14	6.29	42.22	73.66 (48.96 to 97.46)
Poland	PL634	563,208	4.14	10.65	2.37	4.14	28.41	49.72 (33.14 to 65.1)
Poland	PL636	329,251	5.06	14.17	2.02	5.06	37.46	63.78 (43.53 to 86.05)
Poland	PL637	221,707	6.01	13.53	1.50	4.51	33.08	58.64 (39.09 to 78.18)
Poland	PL638	426,014	7.04	13.30	2.35	4.69	35.99	63.38 (41.47 to 82.94)
Poland	PL711	696,954	14.35	23.44	4.30	4.78	52.13	99 (66.48 to 132)
Poland	PL712	383,953	12.15	19.10	3.47	3.47	42.54	80.74 (53.83 to 107.65)
Poland	PL713	586,028	11.94	18.77	3.41	3.41	40.95	78.49 (52.9 to 104.66)
Poland	PL714	446,113	11.21	20.17	3.74	3.74	42.59	81.44 (54.55 to 108.34)
Poland	PL715	362,769	11.95	18.38	3.68	3.68	44.11	81.78 (55.13 to 109.34)
Poland	PL721	760,527	11.40	17.09	3.94	3.07	39.45	74.95 (50.4 to 100.37)
Poland	PL722	479,318	12.52	19.47	4.87	3.48	43.12	83.45 (55.63 to 110.57)
Poland	PL811	301,395	7.74	13.27	5.53	3.32	38.71	68.57 (46.45 to 92.9)
Poland	PL812	625,874	8.52	15.45	7.46	3.20	42.07	76.69 (51.13 to 101.19)
Poland	PL814	708,790	8.94	13.17	5.17	2.82	36.21	66.31 (44.21 to 87.94)
Poland	PL815	479,708	10.42	15.29	5.56	3.47	40.30	75.05 (50.03 to 99.37)

Poland	PL821	473,345	7.04	15.49	4.93	2.82	33.80	64.08 (42.96 to 85.21)
Poland	PL822	387,310	7.75	14.63	6.02	2.58	35.29	66.27 (43.89 to 87.78)
Poland	PL823	622,818	8.03	13.92	4.28	2.14	31.04	59.41 (39.6 to 79.21)
Poland	PL824	601,116	8.87	13.86	3.88	2.22	32.16	61 (41.03 to 80.96)
Poland	PL841	503,271	6.62	11.26	5.30	3.31	35.77	62.26 (41.73 to 83.45)
Poland	PL842	389,480	8.56	14.55	5.99	4.28	43.65	77.03 (51.35 to 101.85)
Poland	PL843	265,529	6.28	12.55	5.02	3.77	37.66	65.28 (43.94 to 87.87)
Poland	PL911	1,745,765	11.27	15.85	4.20	3.63	40.67	75.61 (50.6 to 100.62)
Poland	PL912	623,448	9.09	12.30	3.74	3.21	33.15	61.49 (40.63 to 80.73)
Poland	PL913	622,615	9.10	12.85	3.21	3.21	33.19	61.57 (41.22 to 81.38)
Poland	PL921	611,056	10.91	15.82	4.36	3.27	39.28	73.64 (49.64 to 98.74)
Poland	PL922	340,945	9.78	14.67	3.91	3.91	41.06	73.33 (48.88 to 97.77)
Poland	PL923	328,465	10.15	15.22	3.04	4.06	39.58	72.05 (48.71 to 96.41)
Poland	PL924	383,413	8.69	13.04	4.35	3.48	39.12	68.68 (46.08 to 91.29)
Poland	PL925	418,866	9.55	13.53	4.77	3.18	38.20	69.23 (46.95 to 93.11)
Poland	PL926	259,017	11.58	16.73	3.86	3.86	41.18	77.22 (50.19 to 100.38)
Portugal	PT111	235,065	4.25	12.76	1.42	9.93	55.30	83.66 (55.3 to 109.19)
Portugal	PT112	405,642	4.93	7.40	0.82	5.75	32.87	51.77 (34.51 to 68.2)
Portugal	PT119	416,941	6.40	7.99	0.80	5.60	34.38	55.16 (36.78 to 72.75)
Portugal	PT11A	1,723,424	7.16	8.51	0.58	7.74	38.68	62.67 (41.78 to 83.17)
Portugal	PT11B	88,642	7.52	15.04	0.00	7.52	71.45	101.53 (67.69 to 139.14)
Portugal	PT11C	422,223	8.68	7.11	0.79	5.53	34.74	56.84 (37.89 to 75)
Portugal	PT11D	194,426	12.00	13.72	1.71	6.86	60.01	94.29 (61.72 to 123.44)
Portugal	PT11E	110,224	6.05	18.14	3.02	6.05	75.60	108.87 (72.58 to 145.16)
Portugal	PT150	441,121	9.07	12.85	3.02	14.36	55.92	95.21 (63.47 to 126.19)
Portugal	PT16B	358,193	6.51	10.24	0.93	12.10	52.11	81.89 (53.97 to 107.95)
Portugal	PT16D	363,904	8.24	9.16	0.92	9.16	42.14	69.62 (46.72 to 93.43)
Portugal	PT16E	440,584	11.35	11.35	0.76	11.35	53.72	88.52 (59.77 to 118.03)
Portugal	PT16F	288,183	8.10	10.41	1.16	11.57	47.42	78.65 (52.05 to 102.94)
Portugal	PT16G	257,697	14.23	11.64	1.29	7.76	58.21	93.13 (62.09 to 124.18)
Portugal	PT16H	83,159	16.03	16.03	4.01	12.03	80.17	128.27 (84.18 to 168.35)
Portugal	PT16I	236,981	12.66	12.66	1.41	12.66	61.89	101.27 (68.92 to 136.44)
Portugal	PT16J	220,208	15.14	16.65	1.51	9.08	77.20	119.58 (80.23 to 158.94)
Portugal	PT170	2,819,219	7.45	8.04	0.95	9.46	43.87	69.76 (46.7 to 92.82)
Portugal	PT181	94,721	14.08	10.56	3.52	14.08	59.82	102.05 (66.86 to 133.73)
Portugal	PT184	119,720	13.92	16.71	2.78	13.92	75.18	122.51 (83.53 to 164.27)
Portugal	PT185	240,930	11.07	11.07	1.38	11.07	58.11	92.7 (62.26 to 124.52)
Portugal	PT186	109,491	15.22	18.27	3.04	12.18	79.15	127.86 (85.24 to 167.44)
Portugal	PT187	157,088	12.73	12.73	2.12	10.61	65.78	103.98 (70.02 to 140.05)
Romania	RO111	567,862	7.04	23.48	7.04	3.52	49.31	90.4 (60.46 to 119.75)

Romania	RO112	281,788	7.10	16.56	8.28	2.37	48.50	82.8 (55.6 to 110.01)
Romania	RO113	702,328	7.59	16.61	6.64	2.85	44.14	77.84 (51.73 to 103.47)
Romania	RO114	466,933	5.71	18.56	8.57	2.86	48.54	84.24 (56.4 to 112.08)
Romania	RO115	337,319	6.92	20.75	7.91	2.96	47.43	85.97 (57.31 to 114.63)
Romania	RO116	216,494	7.70	21.56	7.70	3.08	50.81	90.84 (61.59 to 121.64)
Romania	RO121	332,147	9.03	19.07	8.03	3.01	52.19	91.33 (61.22 to 121.43)
Romania	RO122	550,975	7.86	13.91	8.47	3.02	45.98	79.25 (52.63 to 104.66)
Romania	RO123	205,567	8.11	14.59	9.73	3.24	48.65	84.32 (55.13 to 110.26)
Romania	RO124	306,648	6.52	15.22	8.70	3.26	50.00	83.7 (55.44 to 110.88)
Romania	RO125	542,265	9.22	15.98	8.61	3.07	49.79	86.67 (57.78 to 114.95)
Romania	RO126	399,966	8.33	15.00	7.50	2.50	45.00	78.34 (52.5 to 104.18)
Romania	RO211	597,822	7.81	16.17	12.27	3.35	53.53	93.12 (62.45 to 123.78)
Romania	RO212	392,372	5.10	17.84	13.59	3.40	53.52	93.45 (62.87 to 124.03)
Romania	RO213	789,230	4.65	12.25	10.98	2.53	41.81	72.22 (48.57 to 96.3)
Romania	RO214	453,441	7.35	18.38	12.50	3.68	57.34	99.24 (66.16 to 131.59)
Romania	RO215	629,130	5.30	15.89	10.60	3.18	47.16	82.12 (55.1 to 109.15)
Romania	RO216	385,157	6.06	15.58	14.71	3.46	53.66	93.47 (61.45 to 122.89)
Romania	RO221	301,240	8.85	16.60	17.70	4.43	61.97	109.55 (73.03 to 146.06)
Romania	RO222	428,287	10.12	17.90	14.79	3.89	61.49	108.18 (72.38 to 143.98)
Romania	RO223	679,694	5.89	12.75	14.71	5.39	49.53	88.28 (58.85 to 116.72)
Romania	RO224	517,695	6.44	14.17	14.17	3.86	50.87	89.5 (59.88 to 119.12)
Romania	RO225	201,894	6.60	14.86	18.16	4.95	59.44	104.01 (69.34 to 138.69)
Romania	RO226	329,329	8.10	16.19	13.16	4.05	55.67	97.17 (65.79 to 130.57)
Romania	RO311	593,066	11.24	15.74	9.55	3.37	51.15	91.05 (60.7 to 120.28)
Romania	RO312	293,771	9.08	17.02	14.75	4.54	57.87	103.26 (69.21 to 137.3)
Romania	RO313	503,059	11.26	14.58	9.94	3.31	49.03	88.13 (58.97 to 117.28)
Romania	RO314	273,616	12.18	18.27	13.40	3.65	58.48	105.99 (70.66 to 140.1)
Romania	RO315	263,312	8.86	16.46	15.19	3.80	55.70	100.01 (67.09 to 132.92)
Romania	RO316	736,620	11.31	16.29	11.77	3.62	55.21	98.2 (65.62 to 130.32)
Romania	RO317	351,969	13.26	24.62	15.15	4.74	69.13	126.91 (84.29 to 168.58)
Romania	RO321	1,838,235	11.24	14.69	11.24	3.26	48.23	88.67 (59.11 to 117.69)
Romania	RO322	452,241	8.11	11.06	8.11	2.21	34.64	64.13 (42.75 to 84.76)
Romania	RO411	638,538	12.01	21.40	10.44	3.65	56.38	103.88 (69.43 to 138.34)
Romania	RO412	325,621	13.31	17.40	8.19	3.07	49.14	91.11 (60.4 to 120.79)
Romania	RO413	250,757	11.96	23.93	10.63	3.99	58.49	109 (73.11 to 144.89)
Romania	RO414	409,859	12.20	19.52	11.39	4.07	57.74	104.91 (69.94 to 138.26)
Romania	RO415	358,236	10.24	13.96	8.37	2.79	43.73	79.09 (52.11 to 104.21)
Romania	RO421	422,904	8.67	27.59	7.88	3.94	55.17	103.25 (68.57 to 135.57)
Romania	RO422	280,690	9.50	27.31	9.50	3.56	59.38	109.25 (72.44 to 144.88)
Romania	RO423	396,074	11.78	21.88	9.26	3.37	59.75	106.04 (70.69 to 141.39)

Romania	RO424	698,022	6.21	21.49	5.73	2.87	42.02	78.32 (52.53 to 104.1)
Serbia	RS110	1,687,132	10.08	25.88	7.11	3.56	47.81	94.44 (63.22 to 125.66)
Serbia	RS121	175,348	7.60	39.92	7.60	5.70	62.73	123.56 (81.74 to 163.48)
Serbia	RS122	281,203	9.48	30.82	8.30	3.56	55.71	107.87 (72.31 to 144.62)
Serbia	RS123	617,951	8.09	26.97	6.47	3.78	46.39	91.7 (60.95 to 121.37)
Serbia	RS124	138,371	7.23	38.54	7.23	4.82	65.04	122.86 (84.31 to 166.22)
Serbia	RS125	180,349	7.39	36.97	7.39	3.70	57.30	112.74 (73.93 to 147.86)
Serbia	RS126	177,307	9.40	33.84	7.52	3.76	58.28	112.8 (77.08 to 152.28)
Serbia	RS127	300,988	11.07	29.90	7.75	4.43	53.16	106.32 (70.88 to 140.65)
Serbia	RS211	271,080	6.15	27.05	8.61	4.92	59.02	105.75 (70.09 to 140.18)
Serbia	RS212	165,274	10.08	32.27	10.08	6.05	62.52	121.01 (80.67 to 161.35)
Serbia	RS213	283,008	8.24	31.80	10.60	4.71	58.89	114.25 (76.56 to 150.76)
Serbia	RS214	202,027	9.90	29.70	8.25	4.95	61.05	113.85 (75.9 to 150.14)
Serbia	RS215	202,026	11.55	34.65	9.90	4.95	67.65	128.7 (85.8 to 171.6)
Serbia	RS216	226,807	10.29	32.33	10.29	4.41	66.14	123.45 (82.3 to 164.6)
Serbia	RS217	305,955	5.45	22.88	7.63	3.27	49.03	88.25 (58.83 to 117.66)
Serbia	RS218	284,958	9.36	26.90	8.19	3.51	52.64	100.6 (67.85 to 134.52)
Serbia	RS221	114,815	5.81	40.65	11.61	5.81	72.58	136.45 (92.9 to 182.9)
Serbia	RS222	170,208	11.75	37.21	11.75	5.88	70.50	137.09 (90.09 to 178.21)
Serbia	RS223	109,635	9.12	42.57	12.16	6.08	82.09	152.02 (100.33 to 200.67)
Serbia	RS224	203,253	8.20	31.16	11.48	4.92	65.60	121.36 (80.36 to 160.72)
Serbia	RS225	364,157	8.24	28.38	10.07	4.58	59.50	110.76 (74.14 to 146.46)
Serbia	RS226	85,964	3.88	34.90	11.63	3.88	77.55	131.84 (93.06 to 182.25)
Serbia	RS227	189,091	10.58	28.21	8.81	3.53	54.65	105.77 (70.51 to 141.03)
Serbia	RS228	198,671	5.03	25.17	10.07	3.36	55.37	98.99 (65.43 to 130.87)
Serbia	RS229	85,287	7.82	35.18	11.73	3.91	74.26	132.88 (89.89 to 179.79)
Sweden	SE110	2,251,672	2.07	7.11	1.33	3.55	27.98	42.04 (27.98 to 55.81)
Sweden	SE121	358,363	1.86	6.51	0.93	3.72	29.76	42.79 (28.83 to 57.67)
Sweden	SE122	285,954	2.33	9.33	1.17	4.66	39.63	57.12 (38.47 to 76.94)
Sweden	SE123	449,342	2.23	8.90	1.48	4.45	36.35	53.41 (35.61 to 71.22)
Sweden	SE124	293,253	2.27	9.09	1.14	4.55	37.51	54.56 (35.24 to 70.47)
Sweden	SE125	266,176	2.50	8.77	1.25	3.76	37.57	53.85 (36.32 to 72.63)
Sweden	SE211	350,518	1.90	10.46	1.90	4.75	37.09	56.11 (37.09 to 74.18)
Sweden	SE212	193,161	1.73	12.08	1.73	5.18	36.24	56.95 (37.96 to 75.93)
Sweden	SE213	239,779	2.78	13.90	2.78	6.95	44.49	70.9 (47.27 to 93.14)
Sweden	SE214	57,811	0.00	11.53	0.00	5.77	46.13	63.43 (46.13 to 92.25)
Sweden	SE221	157,059	2.12	16.98	2.12	6.37	44.57	72.16 (48.81 to 95.51)
Sweden	SE224	1,315,447	1.27	13.94	1.52	5.07	35.48	57.27 (38.52 to 76.27)
Sweden	SE231	317,652	1.05	12.59	1.05	5.25	36.73	56.67 (37.78 to 75.55)
Sweden	SE232	1,660,815	1.81	10.24	1.20	4.62	35.93	53.79 (36.13 to 71.85)

Sweden	SE311	277,582	2.40	10.81	1.20	4.80	45.63	64.85 (44.43 to 87.66)
Sweden	SE312	282,657	2.36	8.25	1.18	3.54	41.27	56.61 (37.74 to 76.65)
Sweden	SE313	283,007	2.36	8.24	1.18	4.71	43.58	60.07 (40.05 to 80.09)
Sweden	SE321	244,625	2.73	8.18	1.36	4.09	44.97	61.32 (40.88 to 81.76)
Sweden	SE322	128,155	2.60	7.80	2.60	2.60	46.82	62.42 (39.02 to 78.03)
Sweden	SE331	265,022	1.26	5.03	1.26	3.77	36.47	47.79 (31.44 to 64.15)
Sweden	SE332	250,396	2.66	5.32	1.33	2.66	42.60	54.58 (35.94 to 71.89)
Slovenia	SI	2,064,959	3.87	26.31	2.58	4.04	36.48	73.29 (49.07 to 97.34)
Slovakia	SK010	637,796	3.14	26.13	2.61	2.61	33.97	68.46 (45.99 to 90.94)
Slovakia	SK021	560,476	3.57	27.36	2.97	2.97	37.47	74.34 (49.36 to 98.13)
Slovakia	SK022	589,337	3.39	26.58	3.39	2.83	37.90	74.09 (49.21 to 98.42)
Slovakia	SK023	681,730	3.91	30.32	3.91	3.42	43.03	84.59 (56.23 to 111.97)
Slovakia	SK031	690,671	2.90	24.61	3.38	2.90	37.64	71.43 (47.78 to 94.59)
Slovakia	SK032	652,420	3.58	27.08	4.09	3.07	41.90	79.7 (53.14 to 105.25)
Slovakia	SK041	821,703	2.43	19.88	4.06	2.43	32.05	60.85 (40.57 to 80.32)
Slovakia	SK042	797,384	2.51	21.32	4.60	2.51	34.70	65.63 (43.89 to 87.37)
United Kingdom	UKC1	1,192,182	5.03	6.99	0.56	4.47	44.74	61.79 (41.38 to 82.2)
United Kingdom	UKC2	1,443,148	5.08	6.70	0.69	4.62	45.73	62.83 (42.04 to 83.61)
United Kingdom	UKD1	498,537	4.68	7.35	0.67	4.68	50.15	67.53 (45.47 to 90.26)
United Kingdom	UKD3	2,776,784	4.92	6.00	0.60	3.48	37.93	52.94 (35.29 to 70.35)
United Kingdom	UKD4	1,482,913	4.95	6.74	0.67	4.05	43.83	60.24 (40.24 to 80.02)
United Kingdom	UKD6	921,811	5.06	6.51	0.72	3.62	41.22	57.13 (37.97 to 75.94)
United Kingdom	UKD7	1,536,657	4.77	6.72	0.65	4.12	44.69	60.95 (40.56 to 80.91)
United Kingdom	UKE1	927,908	6.11	7.54	0.72	4.67	42.39	61.43 (40.95 to 81.55)
United Kingdom	UKE2	815,782	5.72	6.95	0.82	4.09	42.09	59.66 (39.63 to 78.45)
United Kingdom	UKE3	1,384,492	6.02	6.26	0.48	3.61	39.24	55.62 (37.08 to 73.91)
United Kingdom	UKE4	2,292,623	4.94	5.67	0.58	3.34	36.78	51.32 (34.46 to 68.48)
United Kingdom	UKF1	2,178,384	6.27	6.43	0.61	3.67	39.02	56 (37.34 to 74.37)
United Kingdom	UKF2	1,799,634	5.93	6.11	0.56	3.33	34.27	50.2 (33.34 to 66.5)
United Kingdom	UKF3	744,099	7.62	8.06	0.90	4.48	44.35	65.4 (43.9 to 87.35)
United Kingdom	UKG1	1,332,335	6.25	6.76	0.50	4.00	41.53	59.04 (39.53 to 78.56)
United Kingdom	UKG2	1,607,817	5.80	6.43	0.62	3.73	41.05	57.64 (38.35 to 76.29)
United Kingdom	UKG3	2,866,374	5.35	5.93	0.47	3.37	36.05	51.17 (34.31 to 68.15)
United Kingdom	UKH1	2,483,713	7.52	9.13	0.67	4.56	40.40	62.27 (41.74 to 82.94)
United Kingdom	UKH2	1,834,041	6.00	6.54	0.55	3.27	32.35	48.71 (32.53 to 64.7)
United Kingdom	UKH3	1,806,072	7.57	8.86	0.55	4.43	40.05	61.46 (41.16 to 81.76)
United Kingdom	UKI3	1,157,206	3.74	4.32	0.29	2.30	20.45	31.11 (20.74 to 41.48)
United Kingdom	UKI4	2,350,619	3.40	3.83	0.28	1.99	18.43	27.94 (18.58 to 37.01)
United Kingdom	UKI5	1,889,231	5.12	6.00	0.35	3.00	27.70	42.17 (28.23 to 55.93)
United Kingdom	UKI6	1,292,194	5.16	5.93	0.52	3.10	28.38	43.08 (28.89 to 57.27)

United Kingdom	UKI7	2,065,361	4.52	5.16	0.32	2.58	24.53	37.12 (24.85 to 49.39)
United Kingdom	UKJ1	2,375,944	5.19	5.89	0.42	3.23	30.44	45.18 (30.16 to 59.91)
United Kingdom	UKJ2	2,861,061	6.64	8.85	0.58	4.89	40.43	61.4 (41.01 to 81.55)
United Kingdom	UKJ3	1,966,614	5.42	7.29	0.51	4.58	37.97	55.76 (37.29 to 74.24)
United Kingdom	UKJ4	1,815,885	6.42	8.81	0.55	4.77	38.37	58.92 (39.47 to 78.57)
United Kingdom	UKK1	2,463,549	5.28	6.22	0.41	3.92	37.89	53.72 (35.99 to 71.58)
United Kingdom	UKK2	1,317,621	5.57	8.60	0.51	5.82	48.83	69.32 (46.3 to 92.09)
United Kingdom	UKK3	557,641	2.99	7.17	0.60	6.58	53.80	71.13 (47.82 to 95.04)
United Kingdom	UKK4	1,175,720	4.25	7.65	0.57	5.95	50.47	68.89 (45.93 to 91.58)
United Kingdom	UKL1	1,956,933	4.77	7.15	0.51	5.11	50.59	68.13 (45.65 to 90.62)
United Kingdom	UKL2	1,154,993	4.62	6.06	0.58	4.04	41.27	56.57 (37.81 to 75.33)
United Kingdom	UKM5	492,249	3.39	4.74	0.68	4.06	41.31	54.17 (35.89 to 71.78)
United Kingdom	UKM6	469,239	3.55	4.97	0.71	4.26	52.57	66.06 (44.04 to 87.38)
United Kingdom	UKM7	1,968,298	4.06	5.25	0.51	3.90	44.71	58.43 (38.95 to 77.56)
United Kingdom	UKM8	1,524,674	3.94	5.25	0.66	3.72	47.66	61.22 (40.88 to 81.55)
United Kingdom	UKM9	945,737	4.58	6.34	0.70	4.23	52.16	68.02 (45.82 to 90.93)
United Kingdom	UKN0	1,861,468	2.69	4.30	0.36	2.69	37.60	47.63 (31.87 to 63.57)

Table S7. Statistics for O_3 concentrations for all the tagged countries for the weeks 18-39 of 2015-2017

All rural background stations below 1000m above sea level were used in the evaluation. The metrics are mean bias (MB), normalized mean Bias (NMB), root mean squared error (RMSE), normalized root mean squared error (NRMSE) and Pearson correlation coefficient (PCC). Also shown are the number of observations (N), the mean of the observations (MO) and the modelled mean (MM).

**Country**	**MB** (µg/m^3)	**NMB** (%)	**RMSE** (µg/m^3)	**NRMSE** (%)	**PCC**	**N**	**MO** (µg/m^3)	**MM** (µg/m^3)
Albania	nan	nan	nan	nan	nan	0	nan	nan
Austria	-1.41	-4.7	9.40	19.23	0.61	14383	48.73	47.32
Belgium	3.52	8.3	8.54	20.80	0.81	8412	41.03	44.56
Bulgaria	nan	nan	nan	nan	nan	0	nan	nan
Switzerland	-0.44	-2.2	9.54	19.17	0.61	3329	49.73	49.28
Cyprus	3.25	6.1	6.12	11.00	0.56	425	55.65	58.90
Czechia	-2.45	-6	9.03	18.57	0.70	9695	48.38	45.93
Germany	0.26	-0.9	8.91	19.77	0.75	33036	44.99	45.25
Denmark	3.32	6.4	6.93	17.23	0.64	873	40.22	43.54
Estonia	3.39	10.1	7.34	19.37	0.70	1363	37.93	41.31
Greece	0.41	2.3	2.20	4.10	0.15	136	17.88	18.29
Spain	4.00	7.4	8.79	17.70	0.62	22989	49.73	53.73
Finland	1.73	7.5	6.42	19.40	0.68	4226	33.21	34.95
France	1.19	1.2	7.88	17.60	0.75	22898	44.85	46.04
Croatia	0.14	5.6	9.52	19.53	0.57	1472	48.77	48.92
Hungary	-2.11	-6.3	9.91	20.17	0.55	1008	49.18	47.07
Ireland	6.46	23.6	8.53	26.07	0.74	1274	32.72	39.18
Iceland	nan	nan	nan	nan	nan	0	nan	nan
Italy	0.23	0.6	10.70	19.63	0.53	26480	54.54	54.77
Liechtenstein	nan	nan	nan	nan	nan	0	nan	nan
Lithuania	2.92	6.8	7.68	19.87	0.65	1884	38.81	41.73
Luxembourg	-1.39	-3.4	9.03	19.87	0.79	1352	45.52	44.12
Latvia	4.40	12.2	6.69	19.60	0.73	447	34.16	38.56
Montenegro	nan	nan	nan	nan	nan	0	nan	nan
Malta	5.99	14.8	8.87	16.20	0.48	436	54.96	60.95
Netherlands	5.59	14.7	9.02	23.27	0.77	7720	38.79	44.38
Norway	5.07	14.3	8.26	23.57	0.57	3507	35.01	40.08
Poland	-0.34	-4.5	8.12	18.17	0.72	8342	44.54	44.20
Portugal	7.48	17.6	11.07	25.27	0.70	4731	43.86	51.34
Romania	9.44	25.1	15.94	43.20	0.26	2690	37.05	46.49
Serbia	-6.32	-13	10.13	18.40	0.51	412	54.97	48.65
Sweden	1.98	7.9	6.64	18.33	0.66	4794	36.25	38.23
Slovenia	-2.71	-4.3	8.45	16.30	0.67	1344	51.71	49.01
Slovakia	0.71	1	8.98	19.53	0.49	1703	45.90	46.61

United Kingdom	6.02	16.4	8.40	23.60	0.72	8833	35.64	41.66
Mean (SD)	2.05 (3.59)	5.49 (9.25)	8.77 (1.94)	20.26 (5.46)	0.64 (0.13)	-	44.16 (7.15)	46.21 (5.92)

Table S8. EEA stations by country and year with Modelling Quality Indicator (MQI) ≤ 1
100% of the stations feature a Modelling Quality Indicator (MQI) ≤ 1

Country	2015	2016	2017
Albania	nan	nan	nan
Austria	32/32	32/32	32/32
Belgium	19/19	19/19	19/19
Bulgaria	nan	nan	nan
Switzerland	7/7	7/7	8/8
Cyprus	1/1	1/1	1/1
Czechia	22/22	22/22	23/23
Germany	74/74	74/74	75/75
Denmark	2/2	2/2	2/2
Estonia	3/3	3/3	3/3
Greece	1/1	1/1	nan
Spain	52/52	53/53	53/53
Finland	11/11	10/10	8/8
France	52/52	51/51	52/52
Croatia	4/4	4/4	4/4
Hungary	2/2	2/2	4/4
Ireland	1/1	1/1	1/1
Iceland	nan	nan	nan
Italy	61/61	62/62	65/65
Liechtenstein	nan	nan	nan
Lithuania	5/5	5/5	5/5
Luxembourg	3/3	3/3	3/3
Latvia	1/1	1/1	1/1
Montenegro	nan	nan	nan
Malta	1/1	1/1	1/1
Netherlands	18/18	18/18	18/18
Norway	8/8	8/8	8/8
Poland	18/18	19/19	20/20
Portugal	11/11	12/12	10/10
Romania	7/7	6/6	9/9
Serbia	1/1	1/1	1/1
Sweden	10/10	11/11	11/11
Slovenia	3/3	3/3	3/3
Slovakia	4/4	4/4	4/4
United Kingdom	21/21	22/22	20/20

Total	100% (455/455)	100% (460/460)	100% (467/467)
