# Supplementary material for: Geographic sources of ozone air pollution and mortality burden in Europe
Source: Nat Med. 2024 Jun 3;30(6):1732–8. doi: 10.1038/s41591-024-02976-x (PMC11186783; doi:10.1038/s41591-024-02976-x)
Supplement: Supplementary file 2 — Reporting Summary [file 41591_2024_2976_MOESM2_ESM.pdf]

nature portfolio

Corresponding author(s): Hicham Achebak

Last updated by author(s): Mar 26, 2024

Reporting Summary

Nature Portfolio wishes to improve the reproducibility of the work that we publish. This form provides structure for consistency and transparency in reporting. For further information on Nature Portfolio policies, see our Editorial Policies and the Editorial Policy Checklist.

Statistics

For all statistical analyses, confirm that the following items are present in the figure legend, table legend, main text, or Methods section.

n/a	Confirmed	
☐	☒	The exact sample size (*n*) for each experimental group/condition, given as a discrete number and unit of measurement
☒	☐	A statement on whether measurements were taken from distinct samples or whether the same sample was measured repeatedly
☒	☐	The statistical test(s) used AND whether they are one- or two-sided<br>*Only common tests should be described solely by name; describe more complex techniques in the Methods section.*
☒	☐	A description of all covariates tested
☒	☐	A description of any assumptions or corrections, such as tests of normality and adjustment for multiple comparisons
☐	☒	A full description of the statistical parameters including central tendency (e.g. means) or other basic estimates (e.g. regression coefficient) AND variation (e.g. standard deviation) or associated estimates of uncertainty (e.g. confidence intervals)
☒	☐	For null hypothesis testing, the test statistic (e.g. *F*, *t*, *r*) with confidence intervals, effect sizes, degrees of freedom and *P* value noted<br>*Give P values as exact values whenever suitable.*
☒	☐	For Bayesian analysis, information on the choice of priors and Markov chain Monte Carlo settings
☒	☐	For hierarchical and complex designs, identification of the appropriate level for tests and full reporting of outcomes
☐	☒	Estimates of effect sizes (e.g. Cohen's *d*, Pearson's *r*), indicating how they were calculated

Our web collection on statistics for biologists contains articles on many of the points above.

Software and code

Policy information about availability of computer code

Data collection	No software was used.
Data analysis	The CMAQ code is available at https://github.com/USEPA/CMAQ; the WRF-ARW code at https://github.com/wrf-model/WRF; the HERMESv3 code at https://earth.bsc.es/gitlab/es/hermesv3_gr; and the MEGAN code at https://bai.ess.uci.edu/megan/data-and-code/.

For manuscripts utilizing custom algorithms or software that are central to the research but not yet described in published literature, software must be made available to editors and reviewers. We strongly encourage code deposition in a community repository (e.g. GitHub). See the Nature Portfolio guidelines for submitting code & software for further information.

Data

Policy information about availability of data

All manuscripts must include a data availability statement. This statement should provide the following information, where applicable:

- Accession codes, unique identifiers, or web links for publicly available datasets
- A description of any restrictions on data availability
- For clinical datasets or third party data, please ensure that the statement adheres to our policy

Weekly deaths and population numbers can be freely downloaded from Eurostat (https://ec.europa.eu/eurostat/cache/metadata/en/demomwk_esms.htm; https://ec.europa.eu/eurostat/cache/metadata/en/demo_r_gind3_esms.htm), and O3 simulated values from Zenodo repository (https://doi.org/10.5281/zenodo.10606147)

Human research participants

Policy information about studies involving human research participants and Sex and Gender in Research.

Reporting on sex and gender	Sex-based analysis was not performed because sex-specific exposure-response association between O3 and mortality was not available.
Population characteristics	Not applicable.
Recruitment	Not applicable.
Ethics oversight	Not applicable.

Note that full information on the approval of the study protocol must also be provided in the manuscript.

Field-specific reporting

Please select the one below that is the best fit for your research. If you are not sure, read the appropriate sections before making your selection.

☒ Life sciences ☐ Behavioural & social sciences ☐ Ecological, evolutionary & environmental sciences

For a reference copy of the document with all sections, see nature.com/documents/nr-reporting-summary-flat.pdf

Life sciences study design

All studies must disclose on these points even when the disclosure is negative.

Sample size	Weekly time series of all-cause mortality counts for the warm season (weeks 18-39 [ISO8601], approximately corresponding to the months from May to September) were obtained for the period 2015-2017 from Eurostat. The mortality dataset included 6 291 008 counts of deaths from 813 contiguous regions (NUTS1-3 [Nomenclature of Territorial Units for Statistics]) representing about 530 million people in 35 countries.
Data exclusions	No data exclusions.
Replication	Not applicable dor this study, as it is observational.
Randomization	Not applicable for this study, as aggregate population secondary data are used.
Blinding	Not applicable for this study, as aggregate population secondary data are used.

Reporting for specific materials, systems and methods

We require information from authors about some types of materials, experimental systems and methods used in many studies. Here, indicate whether each material, system or method listed is relevant to your study. If you are not sure if a list item applies to your research, read the appropriate section before selecting a response.

Materials & experimental systems

n/a	Involved in the study	
☒	☐	Antibodies
☒	☐	Eukaryotic cell lines
☒	☐	Palaeontology and archaeology
☒	☐	Animals and other organisms
☒	☐	Clinical data
☒	☐	Dual use research of concern

Methods

n/a	Involved in the study	
☒	☐	ChIP-seq
☒	☐	Flow cytometry
☒	☐	MRI-based neuroimaging
